# Discovery of synthetic small molecules targeting the central regulator of *Salmonella* pathogenicity

**DOI:** 10.1126/sciadv.adr5235

**Published:** 2025-04-11

**Authors:** Abdelhakim Boudrioua, Joe D. Joiner, Iwan Grin, Thales Kronenberger, Vadim S. Korotkov, Wieland Steinchen, Alexander Kohler, Sophie Schminke, Julia-Christina Schulte, Michael Pietsch, Arun Naini, Simon Kalverkamp, Sven-Kevin Hotop, Travis Coyle, Claudio Piselli, Murray Coles, Katharina Rox, Matthias Marschal, Gert Bange, Antje Flieger, Antti Poso, Mark Brönstrup, Marcus D. Hartmann, Samuel Wagner

**Affiliations:** ^1^ Section of Cellular and Molecular Microbiology, Interfaculty Institute of Microbiology and Infection Medicine (IMIT), University of Tübingen, Elfriede-Aulhorn-Str. 6, 72076 Tübingen, Germany.; ^2^ German Center for Infection Research (DZIF), partner-site Tübingen, 72076 Tübingen, Germany.; ^3^ Department of Protein Evolution, Max Planck Institute for Biology Tübingen, Tübingen, Germany.; ^4^ Institute of Medical Microbiology and Hygiene, Interfaculty Institute of Microbiology and Infection Medicine (IMIT), University of Tübingen, Elfriede-Aulhorn-Str. 6, 72076 Tübingen, Germany.; ^5^ School of Pharmacy, Faculty of Health Sciences, University of Eastern Finland, Kuopio 70211, Finland.; ^6^ Department of Chemical Biology, Helmholtz Centre for Infection Research (HZI), 38124 Braunschweig, Germany.; ^7^ Center for Synthetic Microbiology, Philipps University of Marburg, Karl-von-Frisch-Str. 14, 35043 Marburg, Germany.; ^8^ Department of Chemistry, Philipps University of Marburg, Hans Meerwein-Str. 4, 35043 Marburg, Germany.; ^9^ Unit for Enteropathogenic Bacteria and Legionella (FG11) and National Reference Centre for Salmonella and other Bacterial Enterics, Robert Koch Institute (RKI), Burgstr. 37, 38855 Wernigerode, Germany.; ^10^ German Center for Infection Research (DZIF), Partner Site Hannover-Braunschweig, 38124 Braunschweig, Germany.; ^11^ Institute of Pharmacy, Pharmaceutical/Medicinal Chemistry and Tübingen Center for Academic Drug Discovery (TüCAD2), University of Tübingen, Auf der Morgenstelle 8, 72076 Tübingen, Germany.; ^12^ Institute of Organic Chemistry and Biomolecular Drug Research Centre (BMWZ), Leibniz University Hannover, Schneiderberg 1B, 30167 Hannover, Germany.; ^13^ Interfaculty Institute of Biochemistry, University of Tübingen, Tübingen, Germany.; ^14^ Excellence Cluster “Controlling Microbes to Fight Infections” (CMFI), Elfriede-Aulhorn-Str. 6, 72076 Tübingen, Germany.

## Abstract

The enteric pathogen *Salmonella enterica* serovar Typhimurium relies on the activity of effector proteins to invade, replicate, and disseminate into host epithelial cells and other tissues, thereby causing disease. Secretion and injection of effector proteins into host cells is mediated by dedicated secretion systems, which hence represent major virulence determinants. Here, we report the identification of a synthetic small molecule with drug-like properties, C26, which suppresses the secretion of effector proteins and consequently hinders bacterial invasion of eukaryotic cells. C26 binds to and inhibits HilD, the transcriptional regulator of the major secretion systems. Although sharing the same binding pocket as the previously described long-chain fatty acid ligands, C26 inhibits HilD with a unique binding mode and a distinct mechanism. We provide evidence of intramacrophage activity and present analogs with improved potency and suitability as scaffolds to develop antivirulence agents against *Salmonella* infections in humans and animals.

## Teaser

Synthetic molecules targeting HilD provide an antivirulence strategy to disrupt *Salmonella enterica* pathogenicity.

## INTRODUCTION

The threat that antibiotic resistance poses to global public health is well recognized. In 2019, an estimated 1.27 million deaths were attributed to antibiotic-resistant bacterial infections worldwide (*1*). A promising strategy to circumvent antibiotic resistance is the development of drugs targeting virulence factors that are essential for bacterial pathogenesis but not for bacterial growth and viability (*2*–*4*). In contrast to antibiotics that directly inhibit growth or kill the bacteria, nonlethal antivirulence agents are thought to exert a reduced selective pressure for the development of resistant strains (*5*) and preserve the commensal microbiota.

Nontyphoidal salmonellae (NTS) like *Salmonella enterica* subspecies *enterica* serovar Typhimurium are enteric pathogens causing inflammatory diarrhea. Several strains can lead to invasive nontyphoidal *Salmonella* (iNTS) infections once the bacteria invade the intestinal epithelium (*6*, *7*). *S.* Typhimurium invasion of epithelial cells is mediated by secretion systems that are encoded on horizontally acquired *Salmonella* pathogenicity islands (SPIs) and through which effector proteins are exported (*8*). The first step in the pathogenesis of *S.* Typhimurium is adhesion to the host epithelial cells. A giant nonfimbrial adhesin is secreted through the SPI-4–encoded type I secretion system (T1SS) to initiate adhesion (*9*, *10*). A type III secretion system encoded in SPI-1 (T3SS-1) is concomitantly assembled and enables the engulfment, followed by the internalization of bacteria into host epithelial cells (*9*). Once inside the epithelial cells or inside macrophages, bacteria survive and replicate inside the *Salmonella*-containing vacuole (SCV) (*11*), owing to a second T3SS encoded on SPI-2 (T3SS-2) (*12*). Within macrophages, invasive strains of *S.* Typhimurium can disseminate in the bloodstream leading to a life-threatening systemic infection (*13*).

The sequential activation of the different secretion systems requires a finely tuned regulation of the expression of SPI-encoded genes to coordinate the adhesion and injection of virulence factors in response to environmental signals. *S.* Typhimurium has virulence-associated signal transduction systems, which sustain a feed-forward regulatory loop formed by the three transcriptional regulators HilD, HilC, and RtsA (*14*). These three regulators positively modulate each other’s expression, by binding to the promoter regions of their respective encoding genes (*15*, *16*), and activity by forming homo- or heterodimers (*17*). HilD is the main regulator through which the upstream signals feed into the regulatory network (*14*, *18*). HilD positively regulates the transcriptional regulator HilA, known to be an activator of T3SS-1 (*19*, *20*) and T1SS (*11*). HilD is also involved in the regulation of SPI-2 through the transcriptional activation of *ssrAB* (*21*), which code for a two-component system. HilD is therefore considered the central regulator of *S.* Typhimurium invasion-related pathogenicity. A HilD-deficient strain is unable to activate the virulence genes encoded on SPIs (*22*), to invade the cecal tissue, and to elicit inflammation in a mouse model of *S.* Typhimurium gastrointestinal infection (*23*). The importance of the HilD-regulated SPI-1 and SPI-2 has also been demonstrated in a chicken infection model. Oral infection of 1-day-old chickens with SPI-1– or SPI-2–deficient mutants resulted in a strong reduction of intestinal and systemic salmonellosis (*24*).

Considering the critical role of T3SSs at different stages of *S.* Typhimurium pathogenicity, several antivirulence agents targeting T3SS structural proteins (*25*–*28*) and regulatory proteins (*29*–*36*) have been identified. However, none of these inhibitors are actively being developed to treat *Salmonella* infections. In this study, we combined a virtual and phenotypic screen to identify inhibitors of T3SS-1 with drug-like properties. We identified compound C26 as a small molecule inhibiting protein secretion through T1SS, T3SS-1, and T3SS-2. Analysis of the mode of action revealed that C26 leads to a down-regulation of all invasion-associated SPIs by targeting the transcriptional regulator HilD. As a result, treating bacteria with C26 impeded the invasion into host cells. We lastly conducted a structure-activity relationship (SAR) analysis and uncovered analogs with improved potency.

## RESULTS

### Identification of T3SS-1 inhibitors

**Fig. 1.**
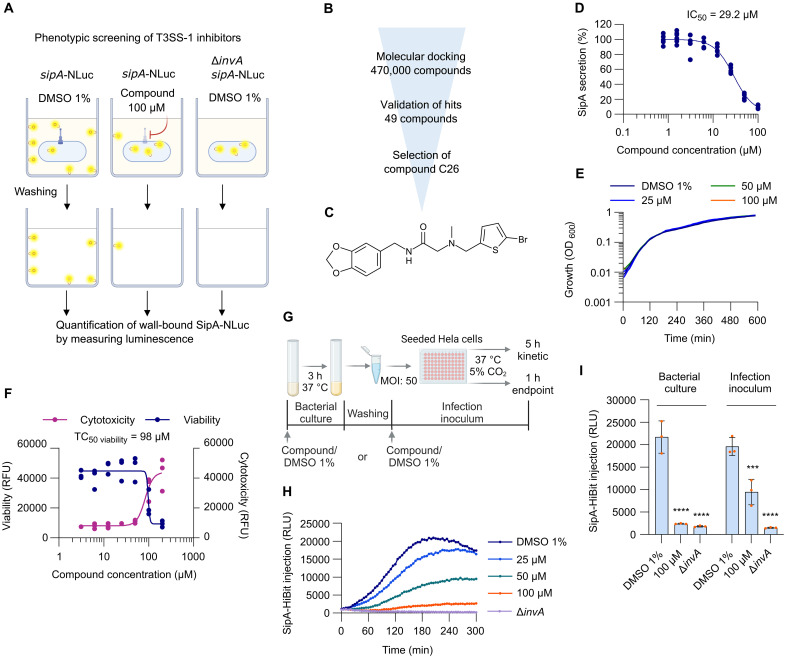
Identification of T3SS-1 inhibitors. (**A**) NanoLuc Luciferase assay to screen T3SS-1 inhibitors in 384-well plates. Compounds were screened at a final concentration of 100 μM (*N* = 3 replicates). Cultures with strains *sipA*-NLuc and Δ*invA*, *sipA*-NLuc with 1% (v/v) DMSO were used as positive and negative controls, respectively. Figure created with BioRender. (**B**) Screening workflow applied to identify T3SS-1 inhibitors. (**C**) Chemical structure of compound C26. (**D**) Dose-response curve of SipA secretion with increasing concentrations of C26. 100% relative SipA secretion corresponds to the luminescence intensity of the WT strain grown in the presence of 1% (v/v) DMSO. The relative SipA secretion of the ∆*invA* mutant was considered as the bottom (*N* = 2 technical replicates within *N* = 3 biological replicates). (**E**) Growth of *S.* Typhimurium SL1344 in LB medium supplemented with C26 at different concentrations or 1% (v/v) DMSO. Experiment performed in a 96-well plate. Growth assessed by measuring the OD at 600 nm (*N* = 3 biological replicates). (**F**) In vitro toxicity in HeLa cells exposed to 100 μM C26 for 18 hours using the ApoTox-Glo assay. Fluorescence was measured as a readout for viability 400_Ex_/505_Em_ and cytotoxicity 485_Ex_/520_Em_ (*N* = 3 biological replicates). (**G**) Description of the experimental plan used to monitor SipA-HiBiT injection into HeLa cells. Figure created with BioRender. h, hours. (**H**) Kinetic of SipA-HiBiT injection intro HeLa cells when the compound or DMSO (1%) was added to the bacterial culture. MOI: 50. Representative replicate from three independent experiments. (**I**) Effect of C26 at 100 μM on the injection of SipA-HiBiT into HeLa cells when added to the bacterial culture or only to the infection inoculum. Endpoint measurement 1 hour postinfection. MOI: 50. ***P < 0.001; ****P < 0.0001 (Bonferroni’s multiple comparisons test). *N* = 3 biological replicates.

To identify inhibitors of *S.* Typhimurium T3SS-1, we first set up an assay to monitor the secretion of the effector protein SipA (Fig. 1A). We then used molecular docking to computationally screen an Enamine library of ~470,000 commercially available compounds against the major export apparatus protein SctV (InvA), resulting in the selection of 49 compounds (Fig. 1B). SipA secretion was monitored as reported previously (*37*) to assess the inhibitory activity of the 49 compounds. The most potent compound, named C26 (CAS number 930993-54-7), was a drug-like small molecule with *N*-benzyl aminoacetamide core and a molecular weight of 397.3 Da (Fig. 1C and table S1).

C26 inhibited the secretion of SipA with an average half-maximal inhibitory concentration (IC_50_) of 29.2 μM [confidence interval (CI): 27.1 to 31.4] (Fig. 1D) and did not impair the growth of *S.* Typhimurium (Fig. 1E). We then assessed mammalian cell toxicity in vitro on HeLa cells using the ApoTox-Glo assay after an exposure of 12 and 18 hours to the compound. After 12 hours, the average half-maximal toxic concentration (TC_50 viability_) could not be calculated because of low toxicity levels (fig. S1). After 18 hours, the compound exhibited an average TC_50 viability_ of 98 μM (Fig. 1F). We further evaluated toxicity in mice at an initial dose of 3 mg/kg in a maximum tolerated dose (MTD) experiment. Neither mortality nor notable adverse effects were observed after oral administration of C26 at 3, 10, and at the highest tested dose of 30 mg/kg in three separate rounds (table S2). The body weight gain in all tested animals was normal after 72 hours of treatment, and no notable abnormalities were observed at termination in all groups (table S3).

To better characterize the T3SS-1 inhibitory activity of C26, a split NanoLuc (HiBiT/LgBiT) system was used to quantify the levels of injected SipA into HeLa cells, as previously described (*37*, *38*). SipA was fused to HiBiT, whereas LgBiT was stably expressed in the cytoplasm of the HeLa cell line. Only if SipA-HiBiT is injected into the HeLa cells, a functional luciferase can be reconstituted by the interaction between LgBiT and HiBiT. Therefore, luminescence intensity inside the host cells can be used as a proxy for the translocation efficiency of SipA-HiBiT. When C26 was added to the bacterial culture (Fig. 1G) and then removed from the medium by centrifugation of the inoculum before infecting HeLa cells, we observed a dose-dependent decrease in SipA injection into HeLa cells over a 5-hour infection time (Fig. 1H). Similarly, the endpoint measurement of SipA injection at 1 hour postinfection under the same conditions showed a strong effect of C26 (100 μM) with 11% residual SipA injection (Fig. 1I). Notably, when bacteria were treated exclusively during the infection of host cells (infection inoculum), SipA injection was reduced to an average of 48% of the untreated bacteria (Fig. 1I). This observation suggests a fast inhibition of SipA injection by the compound without the need for prior treatment of growing bacteria.

### Effect of C26 on the expression of virulence genes and invasion of eukaryotic cells

**Fig. 2.**
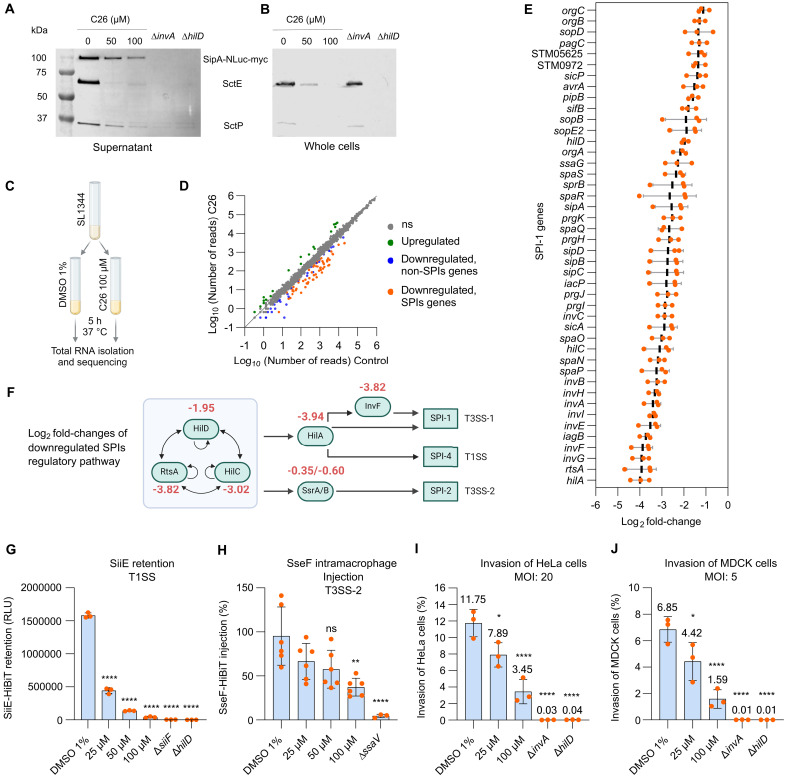
C26 targets the regulatory pathway of SPIs and reduces invasion into host cells. (**A** and **B**) Abundance of T3SS-1 effector proteins in the supernatant (A) and in whole cells (B) monitored by Western blotting. Mouse anti-*myc* (1:1000), anti-SctE (1:1000), and anti-SctP (1:1000) antibodies were used to quantify SipA, SctE (SipB), and SctP (invJ), respectively. (**C**) Experimental plan of the transcriptome analysis by RNA-seq. (**D**) Scatterplot representing the level of gene expression when bacteria were grown in the presence of C26 (100 μM) or DMSO [1% (v/v)] as a control condition. n.s., not significant. Green: Up-regulated genes. Orange: Down-regulated genes encoded in SPIs. Blue: Down-regulated genes that are not encoded in SPIs. Gray: Below statistical cutoff. (**E**) Log_2_ FCs in the expression of SPI-1–encoded genes in the presence of C26 (100 μM) (*N* = 3 biological replicates). (**F**) Regulation of SPI-encoded genes. The log_2_ FCs in gene expression in the presence of C26 (100 μM) are indicated in red. (**G**) Activity of C26 on the cell surface retention of SiiE-HiBiT. ∆*siiF* and ∆*hilD* mutants were used as controls for lack of SiiE secretion and expression, respectively. *N* = 3 biological replicates. (**H**) Activity of C26 on T3SS-2 as quantified by measuring the injection of SseF-HiBiT inside macrophages RAW 264.7 expressing LgBiT. The ∆*ssaV* mutant was used as a control for the lack of T3SS-2 activity. MOI: 10. *N* = 6 biological replicates, except for Δ*ssaV* mutant for which *N* = 3 biological replicates. (**I** and **J**) Invasion of HeLa cells, MOI: 20 (I) and MDCK cells, MOI: 5 (J) by *S.* Typhimurium in the presence of C26. ∆*invA* and ∆*hilD* mutants were used as negative controls. *N* = 3 biological replicates. *P < 0.05; **P < 0.01; ****P < 0.0001 (Bonferroni’s multiple comparisons test).

We analyzed the expression and secretion of the T3SS-1–secreted proteins SipA, SctE (SipB), and SctP (InvJ) by Western blotting (Fig. 2, A and B). SipA, SctE, and SctP secretion into the culture supernatant was reduced in a dose-dependent manner when bacteria were grown in the presence of C26 (Fig. 2A). As expected, no secretion was detected in ∆*invA* and ∆*hilD* mutants, which are deficient in T3SS-1 secretion and expression, respectively. Contrary to the ∆*invA* phenotype, C26 blocked the expression of SipA, SctE, and SctP in whole cells, matching the phenotype of the ∆*hilD* mutant (Fig. 2B). These data suggested that C26 may interfere with the regulation of SPI-1. We therefore performed a transcriptome analysis by RNA sequencing (RNA-seq) on bacteria treated with C26 (100 μM) under SPI-1–inducing conditions (Fig. 2C). C26 led to the down-regulation of genes encoded on several SPIs (Fig. 2D), with stronger down-regulation observed for genes encoded on SPI-1 and SPI-4 (Fig. 2E and fig. S2A). The expression of *hilD*, *hilC*, and *RtsA* was down-regulated by a log_2_ fold change (FC) of 1.95, 3.02, and 3.82, respectively (Fig. 2, E and F), and consequently, the expression of *hilA* was also reduced by a log_2_ FC of 3.94. On the basis of these results, C26 was assumed to, besides SPI-1, affect the HilA-regulated SPI-4 encoding the T1SS and, to a lesser extent, the SPI-2 encoding T3SS-2, which are necessary for invasion and survival inside the host cell, respectively.

We then quantified the effect of C26 on the SPI-4 encoded T1SS using the secretion of the giant adhesin SiiE fused to HiBiT (SiiE-HiBiT) as a readout. The deleted *siiF*, encoding for the ABC-transporter component of the T1SS, and Δ*hilD* mutants served as controls for impeded secretion and expression of SPI-4–encoded genes, respectively. We used the Nano-Glo HiBiT extracellular detection system to quantify the SiiE-HiBiT surface retention. When bacteria were grown in the presence of C26, the amount of SiiE-HiBiT retained on the bacterial cell surface was reduced in a dose-dependent manner (Fig. 2G). In addition, we showed by Western blotting analysis that C26 led to a dose-dependent reduction of the expression of SiiF (fig. S2B), confirming the inhibitory activity of C26 on the SPI-4–encoded T1SS.

The activity of C26 on T3SS-2 was investigated by monitoring the secretion of SseF-HiBiT by bacteria localized inside the LgBiT-expressing macrophages. After allowing bacteria to invade the cells, followed by a gentamicin treatment to kill the noninvading bacteria, the compound was added to the infection medium. Luminescence was measured 7 hours after infection. Luminescence values correspond to the amount of SseF-HiBiT secreted by the bacteria localized inside the macrophages. In this experimental setup, C26 (100 μM) reduced SseF-HiBiT secretion to an average of 37% of the control [1% (v/v) dimethyl sulfoxide (DMSO)] (Fig. 2H). To exclude possible interference of the compound’s cytotoxicity with the assay, we exposed the same macrophages cell line to the compound for 6 hours (fig. S3). No toxicity was observed, suggesting that the compound interferes with the activity of the T3SS-2 when *S.* Typhimurium is inside macrophages.

Last, we investigated how C26 affected the invasion of *S.* Typhimurium into HeLa cells (Fig. 2I) and Madin-Darby canine kidney (MDCK) cells (Fig. 2J). Under standard conditions, an average of 11.8 and 6.9% of the original inoculum invaded HeLa cells and MDCK cells, respectively. In the presence of 100 μM C26, the counts of *S.* Typhimurium inside HeLa cells and MDCK cells decreased to 3.5 and 1.6% of the original inoculum, respectively. The effect of C26 on invasion, however, did not reach the level of ∆*invA* and ∆*hilD* mutants, for which invasion was abolished. Our results provide evidence that C26 hinders the invasion of host cells by targeting the regulation of the genes encoded in SPI-1 and SPI-4 and affects the intramacrophage activity of the SPI-2–encoded T3SS-2. The consequence of the latter on the SPI-2–mediated intracellular replication and survival remains to be investigated and is therefore a limitation of the study.

### Identification of the molecular target of C26

**Fig. 3.**
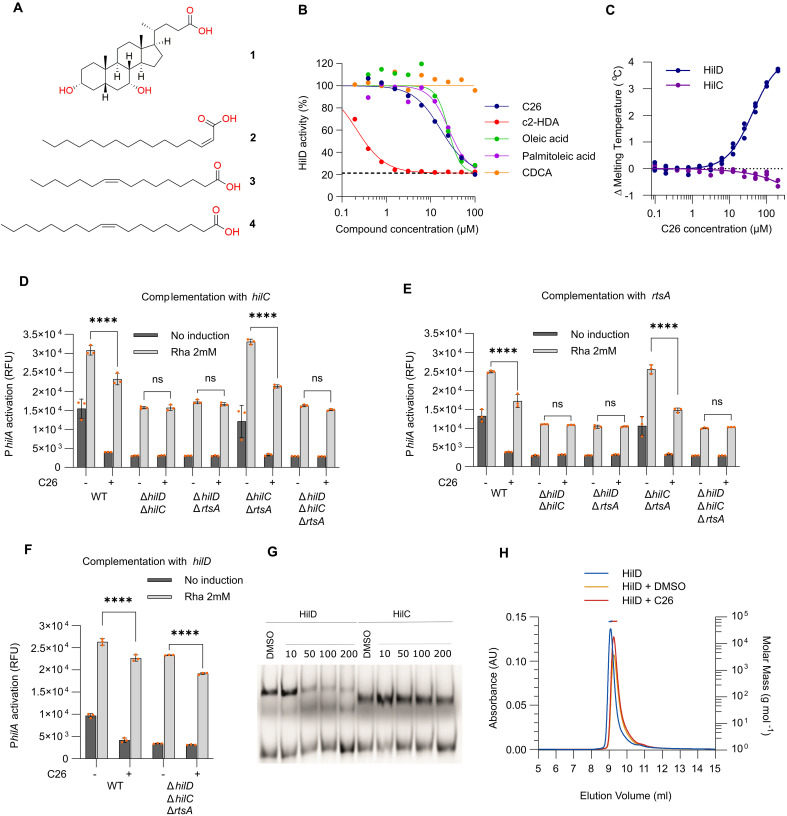
C26 targets the transcriptional regulator HilD. (**A**) Structure of the HilD inhibitors CDCA (**1**), c2-HDA (**2**), palmitoleic acid (**3**), and oleic acid (**4**). (**B**) Whole-cell HilD activity assay. Dose-response curve of P*_hilA_*-sfGFP expression with increasing concentrations of C26 and other known HilD inhibitors. Fluorescence measured at 485_Ex_/510_Em_ (*N* = 3 biological replicates). The dashed line corresponds to the baseline ∆*hilD*. (**C**) Changes in the calculated melting temperature of HilD and HilC upon incubation with increasing concentrations of C26, as determined from the fluorescence at 350 nm by NanoDSF, (*N* = 3 separate experiments). (**D** and **E**) Effect of C26 (100 μM) on P*_hilA_* activation in different background strains complemented with *hilC* (D) or *rtsA* (E). n.s., not significant; ****P < 0.0001 (Bonferroni’s multiple comparisons test). *N* = 3 biological replicates. (**F**) Effect of C26 (100 μM) on strains overexpressing *hilD*. ****P < 0.0001 (Bonferroni’s multiple comparisons test). *N* = 3 biological replicates. (**G**) EMSA showing the effect of C26 on the binding of purified HilD and HilC to the promoter of *hilA*. (**H**) SEC-MALS analysis of HilD in the presence of DMSO (1%) or C26 (100 μM). Left *x* axis shows UV absorbance measured at 280 nm. Right *x* axis shows the calculated molecular weight values from light scattering, highlighted by horizontal dashes. AU, arbitrary units.

To decipher how C26 down-regulates the invasion-associated SPIs, a whole-cell assay was developed to monitor the activity of HilD, HilC, and RtsA. The endogenous *hilA* promoter (P*_hilA_*) was fused with a reporter gene encoding superfolder green fluorescent protein (sfGFP). We first tested the effect of C26 on P*_hilA_* activation in different knockouts of *hilD*, *hilC*, and *rtsA* (fig. S4A). In ∆*hilD* strains, P*_hilA_* activation was at the background noise level. The deletion of *hilC*, *rtsA*, or both, did not affect the activation of P*_hilA_*, and C26 remained as active as in the wild-type (WT) strain. These results are in accordance with previous observations on the minor role of HilC and RtsA in the activation of P*_hilA_* under SPI-1–inducing conditions (*18*). P*_hilA_* activation levels can therefore be used as a proxy of HilD activity. Several HilD inhibitors have been described. Among them, the fatty acids (FAs) oleic acid, palmitoleic acid, *cis*-2-hexadecenoic acid (c2-HDA) (*29*, *32*), and the bile acid chenodeoxycholic acid (CDCA) (*35*) (Fig. 3A). We used the P*_hilA_* activation assay to compare their HilD inhibitory activity with that of C26 (Fig. 3B). Here, C26 exhibited an IC_50_ of 16.9 μM (CI: 14.1 to 20.3 μM). The IC_50_ values of oleic acid and palmitoleic acid were 25.1 and 25.8 μM, respectively. c2-HDA exhibited a strong inhibition of HilD with an IC_50_ of 0.21 μM. No activity of CDCA was observed at the highest tested concentration of 100 μM. These data are in line with the reported activities of the FAs (*39*) and CDCA (*35*) and therefore confirm the reliability of the assay to quantify HilD transcriptional activity.

To assess the affinity of C26 to HilD and HilC in vitro, we used nanoscale differential scanning fluorimetry (nanoDSF), a technique monitoring protein unfolding from intrinsic fluorescence. C26 binding resulted in a dose-dependent increase in the melting temperature of HilD with an apparent *K*_d_ of 30.2 μM, whereas no effect on the thermal stability of HilC was observed (Fig. 3C).

Next, we investigated the selectivity of C26 for HilD using the whole-cell P*_hilA_* activation assay. In the absence of HilD, the pool of HilC and RtsA is insufficient to activate the expression of *hilA*. Therefore, assessing the sensitivity of HilC and RtsA to C26 requires P*_hilA_* to be activated in a HilD-independent manner. To create such a condition, we used a plasmid-encoded *hilC* or *rtsA* under the control of a rhamnose-inducible promoter. Induction of *hilC* (Fig. 3D) or *rtsA* (Fig. 3E) with rhamnose at 2 mM in ∆*hilD* ∆*hilC*, ∆*hilD* ∆*rtsA*, and ∆*hilD* ∆*hilC* ∆*rtsA* knockout mutants resulted in a P*_hilA_* activation level close to that of the WT strain. Under these conditions, in which either *hilC* and/or *rtsA* are the sole activators of P*_hilA_*, C26 did not lead to a reduction of P*_hilA_* activation, as opposed to the WT and the ∆*hilC* ∆*rtsA* backgrounds where P*_hilA_* activation was reduced in the presence of C26. These data suggested that the compound does not impair the activity of HilC and RtsA.

Supposing that HilD is the target of C26, its overexpression would titrate C26 activity and enable P*_hilA_* activation, thus resulting in a resistance mechanism by target overexpression. In the absence of C26, inducing the expression of a plasmid-encoded *hilD* resulted in an increase in P*_hilA_* activation both in the WT background and in the ∆*hilD* ∆*hilC* ∆*rtsA* knockout strain (Fig. 3F). In the presence of C26, P*_hilA_* activation decreased significantly by 14 and 18% in WT and ∆*hilD* ∆*hilC* ∆*rtsA* background strains, respectively, indicating an activity against HilD. The remaining high activation level of P*_hilA_* in the presence of C26 could be explained by the relatively high concentration of induced HilD when compared to the standard pool in the WT strain.

To understand the mechanism of HilD inhibition, we assessed whether C26 impaired the DNA binding activity of HilD using an electrophoretic mobility shift assay (EMSA). HilC, which does not interact with C26, was used as a negative control (Fig. 3G). Recombinant HilD and HilC both bind to a fragment of the *hilA* promoter, encompassing the common A1 binding site (*15*, *16*). In the presence of C26, we observed a dose-dependent inhibition of the DNA binding activity of HilD, whereas no effect on HilC activity was observed.

CDCA and oleic acid have been shown to inhibit the binding of HilD to DNA by disrupting HilD homodimerization (*35*, *39*). We used multiangle light scattering coupled to size exclusion chromatography (SEC-MALS) to investigate whether C26 had a similar effect. However, incubation with equimolar amounts of C26 had no effect on the oligomerization state of HilD, which eluted as a dimer (Fig. 3H and table S4). We confirmed this result by performing BS^3^ [bis(sulfosuccinimidyl)suberate] cross-linking of HilD after incubation with C26 or oleic acid (fig. S4B). Decreased levels of the cross-linked HilD dimer were observed in the presence of oleic acid at 50 and 100 μM, whereas C26 did not affect the levels of cross-linking at the highest tested concentrations of 200 μM. Together, our data suggest that C26 inhibits HilD binding to P*_hilA_* without disrupting its dimerization, a mechanism distinct from that of other tested inhibitors of HilD. We further investigated the effect of C26 on the formation of HilD-HilE heterodimers using a microscale thermophoresis (MST) dimerization assay that we previously described (*39*). In contrast to oleic acid, which disrupted the binding of HilE to HilD, no effect on heterodimerization was observed for C26 (fig. S4C).

### Structural analysis and druggability of HilD

To assess the druggability of HilD, we first performed a bioinformatics analysis. Homologs of *Salmonella* HilD from γ-proteobacteria and a pool of representative homologous sequences, belonging to the AraC/XylS family, were retrieved from the National Center for Biotechnology Information (NCBI)/GenBank using BLAST (accession date 08 January 2022) and from the full draft genomes of the Integrated Microbial Genomes and Microbiomes database (fig. S5). No similar sequences were found in vertebrate genomes. Phylogenetic analyses of the AraC/XylS family showed that HilC and HilD share the highest similarity (fig. S4). The lack of binding of C26 to HilC (Fig. 3C) therefore strengthens the assumption that the compound selectively binds to HilD.

**Fig. 4.**
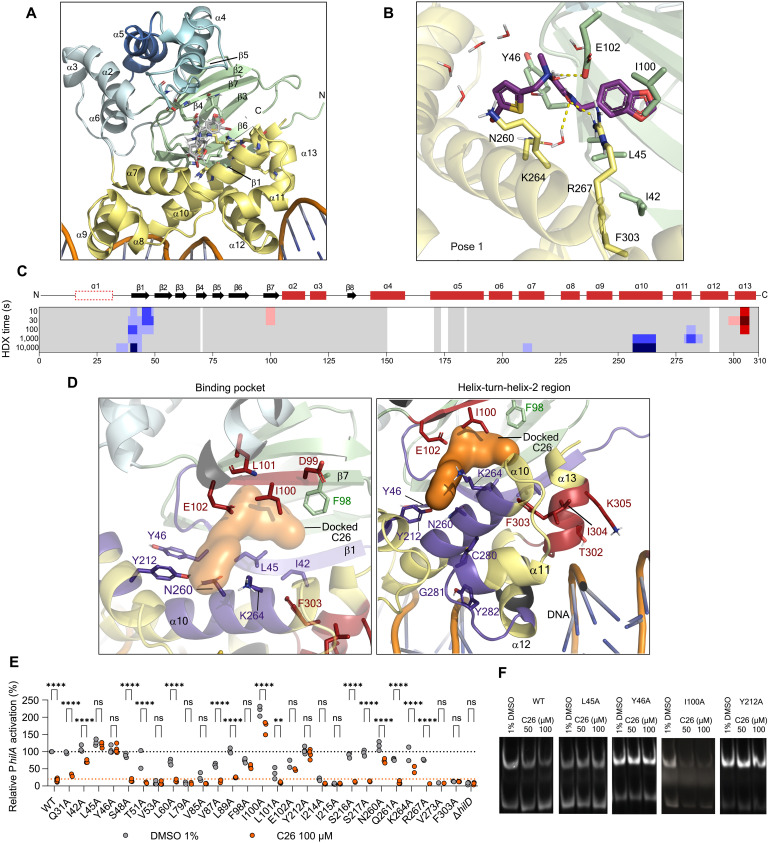
Druggability of HilD and structural characterization of the HilD-C26 complex. (**A**) HilD model with dsDNA generated by AlphaFold. DNA binding domain is highlighted in yellow, bound to a generic dsDNA fragment, whereas the β sheets of the cupin barrel are depicted in green. Dimerization interfaces are displayed in tones of blue and numbered accordingly. (**B**) Structural representation of the proposed binding mode from molecular modeling of pose 1 within the predicted binding pocket, generated by clustering the MD trajectory by the ligand RMSD variation. (**C**) Difference in HDX between C26-bound and apo HilD projected on its amino acid sequence. Different tones of blue or red reflect, respectively, a decrease or an increase in HDX in the presence of C26 (100 μM). The HilD secondary structure is schematically depicted above (red rectangles, α helices; black arrows, β strands). (**D**) Mapping of the regions exhibiting a lower (blue) or higher (red) deuterium incorporation in the presence of C26 (100 μM) as identified by HDX-MS. Zoom on the predicted binding pocket (left), and on the predicted DNA binding HTH-2 region (right). Relevant residues are shown as sticks. Binding pocket volume is depicted in an orange surface. (**E**) Whole-cell assay to monitor the sensitivity of HilD mutants to C26. Bacteria were treated with either DMSO (gray-filled circles, WT: black dotted line) or C26 (orange-filled circles, WT: orange dotted line). P*_hilA_* activation levels by the HilD mutants were calculated relative to HilD_WT_ grown in DMSO 1%. Bonferroni’s multiple comparisons test was applied to compare treated and untreated conditions within each strain and compare the treated mutants to the treated WT condition (see Materials and Methods) (*n* ≥ 2 replicates, except HilD_V273A_ for which *N* = 1 replicate). n.s., not significant; **P < 0.01; ****P < 0.0001 (Bonferroni’s multiple comparisons test). (**F**) EMSAs showing the binding of 600 nM HilD_WT_, HilD_L45A_, HilD_Y46A_, HilD_I100A_, and HilD_Y212A_ to P*_hilA_*, upon incubation with the indicated concentrations of C26.

To gain a deeper understanding of the interaction between C26 and HilD, we used AlphaFold2 to generate a structural model for the HilD core (residues 37 to 308), which was simulated with and without a short DNA fragment (see Materials and Methods). HilD consists of an N-terminal domain (NTD) with a cupin barrel and an all α-helical dimerization interface and a C-terminal DNA binding domain (CTD) with two helix-turn-helix (HTH) motifs (Fig. 4A). We previously identified a pocket involving the cupin barrel and α helices 7 and 10 as the oleic acid binding site (*39*). Assuming the same pocket to bind C26, we identified potential key amino acid positions involved in HilD-ligand interaction using simulations of protonated C26 within this pocket (Fig. 4B). Our docking calculations suggested two different potential binding modes for C26: pose 1 (Fig. 4B and fig. S6), with the bromothiophene establishing a chalcone interaction with the backbone of N260 and a cation-π interaction with K264, and pose 2 (fig. S6), horizontally inverted with this moiety accommodated within the cupin barrel near residues L45 and I100. We performed longer simulations for both poses to derive their relevant protein-ligand interactions and determine the potential binding energy for each pose. Simulations of pose 1 were most stable within HilD’s pocket, as observed by their small variation of the root mean square deviation (RMSD), and low predicted binding energy values (figs. S7 and S8), which together would support this as the preferred binding mode. We could further gain experimental support for this conclusion by nuclear magnetic resonance (NMR) spectroscopy via saturation transfer difference (STD) experiments on the HilD-C26 complex. Intensities in STD experiments are sensitive to the proximity of the ligand to the saturated groups in the protein, here the methyl groups of aliphatic residues. The high concentration of these groups surrounding the cupin barrel binding pocket of HilD allowed the discrimination of the binding poses. We applied the CORCEMA algorithm (*40*–*42*) to predict STD intensities for frames of both molecular dynamics (MD) trajectories and calculate an *R*-factor for each frame reflecting the fit to the experimental data. The trajectory starting from pose 1 has a significantly higher density of frames with lower *R*-factors than that starting from pose 2 (fig. S9).

### Structural characterization of the HilD-C26 complex

To probe the proposed binding model further, we performed hydrogen-deuterium exchange mass spectrometry (HDX-MS) on purified HilD alone (with DMSO as a mock) or in the presence of C26 (100 μM). Differences in deuterium exchange upon incubation with C26 are highlighted along the sequence of HilD (Fig. 4C and fig. S10) and mapped onto the HilD model to highlight the binding pocket and the HTH-2 region (α11 and α12) (Fig. 4D). Decreased HDX was observed for residues 35 to 50, 209 to 212, and 256 to 265, all of which are located in the predicted binding pocket in agreement with our computational model (Fig. 4D). These HDX changes mirror those observed for oleic acid (*39*), supporting the assumption that both compounds bind to the same binding pocket. In contrast to oleic acid binding, areas of decreased HDX induced by C26 in the DNA binding domain of HilD were restricted to a short stretch (residues 279 to 285, α11). An HDX increase was apparent for helix α13 (residues 297 to 305) in the presence of C26, whereas oleic acid exclusively reduced HDX in helices α11 to α13 and the interconnecting linkers (*39*). It is likely that binding of C26 to the pocket enclosed by α10-α7-β1-β7 causes a conformational change in helices α11 and α13, resulting in the loss of affinity to P*_hilA_*. We propose this mechanism to be the mode of action of C26 rather than interference with HilD dimerization as shown for oleic acid (Fig. 3H and fig. S4C).

Next, we developed a plasmid-based system for the fast introduction of point mutations to assess their effect on HilD activity and sensitivity to C26. We then performed an alanine scan on amino acid positions located in the predicted binding pocket (Fig. 4E). F303A inactivated HilD, which confirms the importance of the helix α13 for DNA binding and therefore supports the proposed mode of action of C26. L45A, Y46A, F98A, E102A, and Y212A resulted in a significant full loss of sensitivity to C26 (100 μM), whereas I42A, I100A, N260A, and K264A resulted in a partial loss of sensitivity. We confirmed the resistance of HilD_Y46A_ to C26 by showing that the invasiveness of the mutant into HeLa cells was unaffected by the compound (fig. S11). We then assessed the effect of C26 on the DNA binding of HilD_L45A_, HilD_Y46A_, HilD_I100A_, and HilD_Y212A_ by EMSA and observed that each of the mutants bound to the *hilA* promoter with comparable affinity to HilD_WT_ (fig. S12). C26 did not exert any effect on the DNA binding ability of HilD_L45A_, HilD_Y46A_, and HilD_Y212A_ (Fig. 4F), confirming the resistance of these mutants to C26 and highlighting their resistance mechanism. HilD_I100A_ binding to P*_hilA_* was hindered by the compound, suggesting that its reduced sensitivity to C26 in the whole-cell assay (Fig. 4E) may be attributed to its twofold higher transcriptional regulatory activity as compared to HilD_WT_. Combining data generated by MD simulations, biophysical methods, and whole-cell and in vitro assays, we were able to map the binding pocket, identify the amino acid residues important for C26 action, and confirm the proposed mode of action of the compound.

### Spectrum of activity

**Fig. 5.**
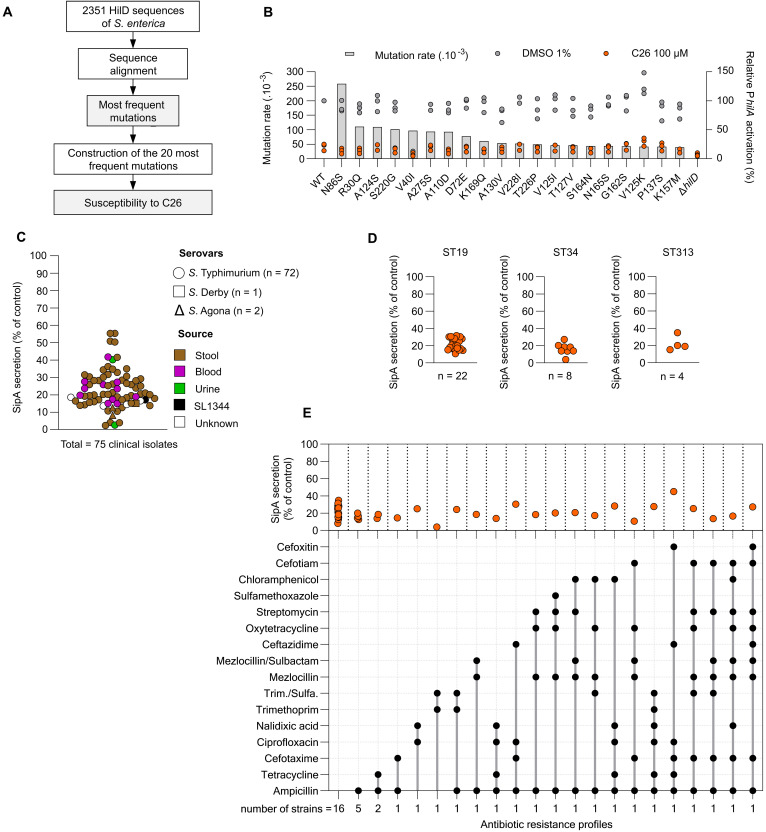
Spectrum of activity. (**A**) Applied workflow for the identification of the most frequent amino acid substitutions in HilD among *S. enterica*. (**B**) Mutation rates of the 20 most frequent substitutions in 2351 sequences of HilD and their consequence on sensitivity to C26 (100 μM). Mutation rates are shown in gray bars (left *y* axis). HilD activity (right *y* axis) was quantified as in Fig. 4E. Bacteria were treated with either 1% DMSO (black dots) or 100 μM C26 (orange dots). (**C**) Activity of C26 (100 μM) on clinical strains of *S. enterica* isolated from human stool (brown), blood (purple), and urine (green) samples. 100% corresponds to SipA secretion in bacteria treated with 1% DMSO. (**D**) Activity of C26 (100 μM) on clinical strains of *S. enterica* isolates clustered by sequence type (ST). 100% corresponds to SipA secretion in bacteria treated with 1% DMSO. (**E**) Combination matrix (bottom) of the phenotypic antibiotic resistance profiles of *S.* Typhimurium clinical isolates (total = 42) and their corresponding sensitivity to C26 (100 μM) as monitored by quantification of SipA secretion (top). 100% corresponds to SipA secretion in bacteria treated with 1% DMSO. Each orange dot corresponds to a single clinical isolate. Trim./Sulfa., trimethoprim/sulfamethoxazole.

Having identified amino acid substitutions leading to resistance of HilD to C26, we searched for the presence of these mutations among 2351 HilD sequences from the NCBI (acquired on 10 June 2023) (Fig. 5A). We first performed a sequence alignment to identify the most frequent substitutions as compared to the reference HilD sequence of SL1344 (table S5). Using the plasmid-based system to monitor HilD activity, we individually introduced the 20 most frequent substitutions into the sequence of HilD and quantified their sensitivity to C26 (Fig. 5B). Except for V40I, which resulted in a nonfunctional HilD, none of the other 19 substitutions affected HilD transcriptional activity. C26 exhibited an inhibitory activity on all the tested variants at a level similar to that of the WT reference sequence.

Last, we aimed to evaluate the spectrum of activity of C26 among clinical isolates of *S. enterica*. We acquired 37 strains of *S.* Typhimurium isolated from patients between 2010 and 2020 at the university hospital of Tübingen, Germany. We selected an additional set of 70 representative clinical *S. enterica* strains, covering different sequence types (fig. S13), and antibiotic resistance profiles (table S6) from the strain collection of the National Reference Centre for *Salmonella* at the Robert Koch Institute, Germany (total = 107 clinical isolates). We used a plasmid-encoded *sipA*-NLuc to assess the secretion levels of SipA through T3SS-1 in the 107 clinical isolates. Thirty-two strains exhibited SipA secretion levels lower than the predefined cutoff of 5% of the reference strain SL1344 and were therefore excluded from further analyses (fig. S14). The remaining 75 strains, regardless of their isolation source (stool: *N* = 58, blood: *N* = 11, or urine: *N* = 2), were all sensitive to C26 (100 μM) (Fig. 5C). Notably, the compound exhibited an activity against *S.* Agona (*N* = 2) and *S.* Derby (*N* = 1). We then clustered the characterized strains according to their clinical multilocus sequence types (MLSTs). Strains belonging to most frequent STs in Germany ST19 (*N* = 22) and ST34 (*N* = 8), and other STs such as ST313 (*N* = 4), were all sensitive to C26 without a reduced sensitivity pattern (Fig. 5D). Last, we clustered the *S.* Typhimurium clinical isolates according to their phenotypic antibiotic resistance profiles (Fig. 5E). The latter had no influence on the inhibitory activity of the compound, strengthening the assumption that antivirulence agents are characterized by a reduced risk of cross-resistance with direct-acting antibiotics. We conclude that C26 has an advantageous activity spectrum among *S.* Typhimurium clinical isolates, regardless of their source of isolation, sequence type, and antibiotic resistance profile.

### SAR analysis

**Fig. 6.**
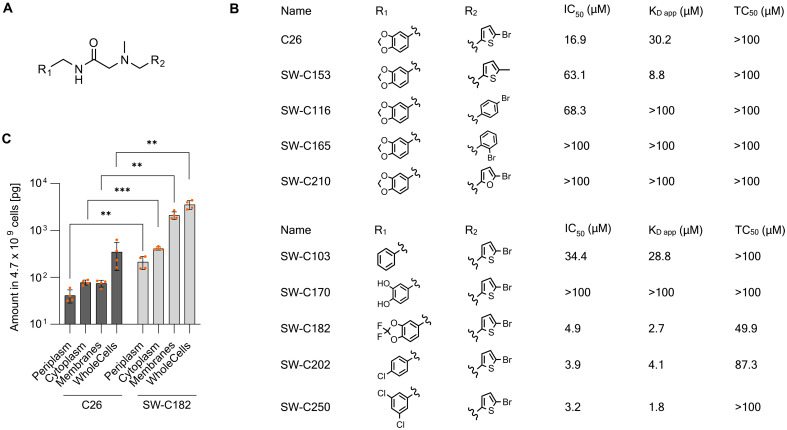
SAR analysis. (**A** and **B**) Structures and activities of C26 and analogs. IC_50_ values were determined with the whole-cell HilD activity assay. *K*_d,app_ values correspond to the in vitro affinity determined by nanoDSF. TC_50_ values correspond to cytotoxicity determined with HeLa cells using the CellTox Green Cytotoxicity Assay. *N* = 3 replicates. (**C**) Quantification of C26 and SW-C182 in subcellular compartments of *S.* Typhimurium. Whole cell is the amount found in unfractionated bacteria. **P < 0.01; ***P < 0.001 (multiple paired *t* tests). *N* = 4 biological replicates.

To gauge the potential for further optimization of C26 potency, we performed an initial SAR analysis. The 5-bromothiophen-2-yl unit turned out to be favorable because its replacement by isosteres such as 2-methylthiophene (SW-C153), 4-bromophenyl (SW-C116), 2-bromophenyl (**SW-C165**), and 2-bromofurane (**SW-C210**) led to less active compounds in the P*_hilA_* activation assay with IC_50_’s of 63.1, 68.3, >100, and >100 μM, respectively (Fig. 6, A and B). The replacement of the benzodioxol moiety allowed a fine-tuning of activities. Compound SW-C103 with a simple phenyl residue, was slightly less potent (IC_50_ = 34.4 μM), whereas the removal of the methylene bridge in the heterocycle to give the catechol (**SW-C170**) led to a complete loss of activity. The 4-chlorophenyl and 3,5-dichlorophenyl compounds SW-C202 and SW-C250 were considerably more potent than C26, with IC_50_’s of 3.9 and 3.2 μM, respectively, whereas TC_50_’s remained in the same range (fig. S15). This improved potency was due to an increased affinity of SW-C202 and SW-C250 to HilD, as reflected by apparent *K*_d_ values obtained by nanoDSF of 4.1 and 1.8 μM, respectively.

To probe whether cellular uptake also contributes to differences in cellular bioactivities, we adapted our method for the quantification of intracellular uptake to *S.* Typhimurium (*43*). The overall uptake of compound SW-C182 was 10-fold higher than that of C26 (Fig. 6C and table S7). In the cytosol, the target compartment of the inhibitors, uptake differed by 5.3-fold. This implies that the activity of C26 and its analogs are driven by both cellular uptake and binding to HilD. In summary, a first round of hit-to-lead led to a 10-fold improved, low micromolar cellular activity, demonstrating the optimization potential of the C26 series.

## DISCUSSION

The slow pace at which direct-acting antibiotics targeting Gram-negative pathogens are being discovered and developed requires the exploration of different approaches. Instead of selecting drug targets that are essential for bacterial survival, we pursued an antivirulence strategy, targeting the invasion-mediating pathogenicity factors of *S. enterica*. We designed a combined in silico and phenotypic screening assay to identify T3SS-1 inhibitors and found a small molecule targeting the transcriptional regulator HilD. Our hit compound (C26) is characterized by a straightforward chemical synthesis and a favorable, Lipinski’s rule-of-five compliant druglikeness (*44*). By combining MD simulations with experimental evidence, we showed that C26 unexpectedly binds to the HilD homodimer to inhibit its binding to P*_hilA_*, identified the binding pocket, and suggested a mechanism of HilD inhibition. Given that the identified druggable pockets of SctV were solvent exposed, highly charged, and hydrophilic, whereas the only determined binding pocket of HilD encompasses the cupin barrel creating a conserved hydrophobic cavity, it is therefore very unlikely that SctV and HilD would share a similar binding pocket.

Several natural compounds have been identified as HilD inhibitors. Plant-derived compounds like the cyclic diarylheptanoid myricanol (*31*) and the flavonoid fisetin (*36*) bind to HilD and inhibit its DNA binding activity. The bile acid CDCA has been shown to interfere with HilD dimerization and DNA binding (*35*). Similarly, long-chain fatty acids (LCFAs) have been shown to bind to HilD and block its dimerization (*32*, *39*). We showed that C26 shares the same binding pocket as CDCA and LCFAs; however, its binding mode is suggested to be different because the inhibition occurs without interfering with the dimerization of the protein.

LCFAs are characterized by a broad spectrum of targets among the AraC-like regulators including HilC and RtsA (*32*, *39*, *45*, *46*), and ToxT and Rns to modulate the virulence of *Vibrio cholerae* (*47*) and *Escherichia coli* (*48*), respectively. In contrast, our hit compound is a synthetic molecule that selectively binds and inhibits HilD activity while remaining inactive against its closest homolog HilC. From a drug development perspective, the latter property is advantageous considering the hurdles associated with polypharmacology (*49*).

Point mutations resulting in resistance to C26 were not found among the 20 most frequent HilD variants that were revealed to be fully sensitive to the compound. In addition, C26 exhibited a good spectrum of activity among *S. enterica* clinical isolates, including strains from ST19, which is associated with gastroenteritis (*50*), and ST313, which is the dominant sequence types in sub-Saharan Africa causing systemic infections (*51*, *52*).

The current hit compound shows promising activity and drug-like properties. Our results further demonstrate that C26 is able to inhibit the secretion of effector proteins of *Salmonella* within infected macrophages. This property is a critical prerequisite for anti-infective drugs targeting Gram-negative intracellular pathogens. Using this compound as a scaffold to perform SAR analysis will be necessary to identify more potent analogs that can serve as drug candidates. Synthetic small molecules targeting HilD could be valuable options for the treatment of *S. enterica* gastrointestinal infections and the prevention of invasive *S. enterica* infections in humans. Considering that secretion systems are essential for the systemic dissemination of NTS in chickens (*24*), it is also conceivable to use HilD inhibitors to prevent invasive *S. enterica* infections in poultry.

## MATERIALS AND METHODS

### Strains and growth conditions

All the strains used in this study are listed in table S8. Except clinical isolates (table S6), all *Salmonella* strains were derived from *S. enterica* serovar Typhimurium SL1344 (*53*) or NCTC 12023 (*54*) (used exclusively for the intramacrophage T3SS-2 injection assay shown in Fig. 3H). Plasmids used in this study are listed in table S9. Primers used for cloning are listed in table S10. *S.* Typhimurium strains were cultured with low aeration at 37°C in Luria-Bertani (LB) medium supplemented with 0.3 M NaCl and the appropriate antibiotic when required.

### Virtual screening of T3SS-1 inhibitors

Virtual screening against T3SS was performed based on the InvA (SctV) C-terminal structure against a commercially available library of ligands. The InvA CTD as a dimer is available with an excellent resolution (*55*) [Protein Data Bank (PDB) ID: 2X49; resolution: 1.50 Å). Potential binding sites were determined using SiteMap, which predicted four potential druggable pockets (DrugScore > 1.0). We proceeded with site 2 (DScore: 1.014), encompassing the region of F388, M505, K512, R544, and M546, which is near the dimerization interface. Ligands were docked within a grid around 12 Å from the centroid of the predicted binding site pocket, as mentioned above.

For this virtual screening step, system preparation and docking calculations were performed using the Schrödinger Drug Discovery suite for molecular modeling (version 2014.1) with standard settings. All ligands were retrieved from Enamine Advanced screening collection (accessed on December 2014 containing 468,436 unique compounds—which is limited by chemical properties: molecular weight ≤ 350 Da, cLogP ≤ 3, and rotB ≤ 7) prepared using LigPrep (*56*) to generate the three-dimensional (3D) conformation, adjust the protonation state to physiological pH (7.4), and calculate the partial atomic charges with the OPLS2005 force field (*57*), generating a total of 1,604,573 states. Docking studies with the prepared ligands were performed using Glide (Glide V7.7) (*58*, *59*) with the Virtual Screening Workflow pipeline that starts docking the total ligand library with high-throughput screening (HTVS) precision and just proceeds with the top 10% of the best scored ligands for single precision (SP) and then their top 10% to extra precision (XP). The final 1000 ligands underwent molecular mechanics with generalized Born and surface area (MM/GBSA) calculations to predict their binding energy. Ligands for testing were selected based on their predicted binding energy and visually inspected for hydrogen bond interactions.

### Sequence similarity search and phylogenetic tree

*Salmonella’s* HilD homologs and a pool of representative homologous sequences containing the AraC domain were retrieved from γ-proteobacteria species. Sequences were retrieved from the NCBI/GenBank using the Blast tool (with scoring matrix BLOSUM45 for distant similar sequences) and from the full draft genomes of the Integrated Microbial Genomes and Microbiomes database (*60*) (with an *e*-value cutoff of 10^−5^) creating a dataset. No similar sequences were found in vertebrate genomes. Sequences renaming and editing were performed with in-house Perl scripts. Sequences with less than 30% global similarity were excluded from further analyses. The full dataset was clustered by similarity (99%) using CD-Hit (*61*), and a set of representative sequences were selected for global alignment using Muscle (*62*). Maximum likelihood phylogenetic tree was generated using PhyML 3.0 (*63*), with posterior probability values (aBayes) as branch statistical support. The substitution model VT was selected for calculations, by ProtTest3 (*64*), based on the highest Bayesian Information Criterion values. All other parameters, with the exception of the equilibrium frequencies, were estimated from the dataset. Dendrogram figures were generated using FigTree v1.4.4 (https://github.com/rambaut/figtree/releases).

### Phenotypic screen of T3SS-1 inhibitors

For compound screening, 50 μl of overnight cultures of *Salmonella* with an approximate optical density at 600 nm (OD_600_) of 2 were diluted in fresh LB medium to an OD_600_ of 0.05 and added to 384-well plates (Nunc MaxiSorp, white) containing 5 nmol of screening compound per well, for a final compound concentration of 100 μM. The plates were incubated for 5 hours at 37°C with shaking at 180 rpm, upon which the bacteria were removed. The plates were washed with phosphate-buffered saline (PBS) [137 mM NaCl, 2.7 mM KCl, 10 mM Na_2_HPO_4_, and 1.8 mM KH_2_PO_4_ (pH 7.4)] using a Tecan HydroSpeed plate washer. Residual PBS after washing was removed, and 50 μl of NanoLuc working solution (NanoGlo, Promega) was added to the wells. Luminescence was then measured using a Tecan Sparc Multimode reader. Cultures with *sipA-*NLuc and Δ*invA sipA-*NLuc strains with 1% (v/v) DMSO were used as positive and negative controls, respectively.

### Chemical synthesis

#### Starting materials

Starting materials were purchased from commercial suppliers (Sigma-Aldrich, TCI, BLDpharm, abcr, Carbolution, Thermo Fisher Scientific, Alfa Aesar, and Acros Organics) and used without further purification. The compounds SW-C153, SW-C116, and SW-C103 are commercially available and were purchased from commercial suppliers.

#### Accurate mass method

High-resolution masses were obtained using a Maxis II TM HD mass spectrometer (Bruker Daltonics, Bremen, Germany).

#### Flash column chromatography

Purification on reversed phase was done with a Pure C-850 FlashPrep system (Büchi) using FlashPure EcoFlex C18 cartridges (Büchi). A gradient of water and acetonitrile was used as an eluent. Dryloads were prepared with silica gel (C18, 0.035 to 0.07 mm, and 400 to 220 mesh; Carl Roth).

Normal phase purification was carried out with a Pure C-810 Flash system (Büchi) using FlashPure cartridges (Büchi). A gradient of cyclohexane and ethyl acetate or dichloromethane and methanol was used as an eluent. Dryloads were prepared with silica gel (60 Å, 230 to 400 mesh, and 40 to 63 μm; Merck).

Conventional column chromatography was carried out with silica gel (60 Å, 230 to 400 mesh, and 40 to 63 μm; Merck) using the eluents described in the synthesis procedures.

#### High-performance liquid chromatography

High-performance liquid chromatography (HPLC) was carried out with a Dionex UltiMate 3000 system (Thermo Fisher Scientific) using a Luna 5 μm C18(2) 100 Å, LC column 250 mm by 21.2 mm, AXIA packed (Phenomenex). As an eluent, water and acetonitrile were used without or with 0.1% formic acid.

NMR spectra of synthesized compounds are provided in data S2.

### Synthesis of compound C26 and analogs

**Fig. 7.**
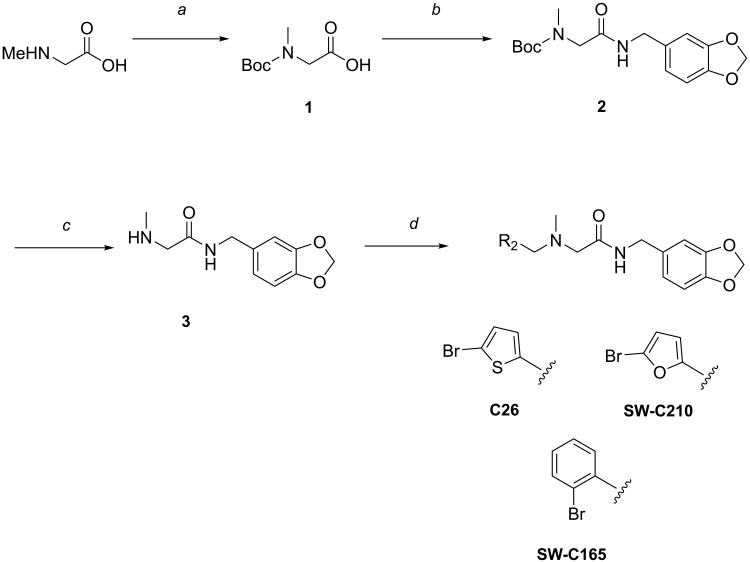
Reagents and conditions for the synthesis of C26, SW-C165, and SW-C210. (**a**) Boc_2_O, KOH, H_2_O/dioxane, r.t., overnight 57%; (**b**) iBuOCOCl, Et_3_N, THF, 0°C to r.t., 2 hours, then piperonylamine, r.t., 2 hours, 84%; (**c**) TFA, CH_2_Cl_2_, r.t., overnight, 72%; (**d**) R_2_CHO, HOAc, THF, r.t., 10 min, then NaBH(OAc)_3_, r.t., overnight.

The synthesis of the compound C26 (CAS number 930993-54-7) and some of its analogs was performed according to the reaction scheme shown in Fig. 7.

### *N*-(*tert*-Butoxycarbonyl)-*N*-methylglycine (1)

Sarcosine (1.00 g, 11.22 mmol, 1 equiv) was dissolved in a mixture of H_2_O (11 ml) and 1,4-dioxane (25 ml), and the solution was cooled to 0°C. KOH (2.73 g, 50.51 mmol, 4.5 equiv), dissolved in H_2_O (4 ml), and di-*tert*-butyl dicarbonate (2.94 g, 13.46 mmol, 1.2 equiv) was added, and the solution was stirred overnight at room temperature (r.t.). Then, 1,4-dioxane was rotary evaporated and the aqueous residue was acidified with 1 N HCl to pH = 3. The solution was extracted with EtOAc (3 x 40 ml), and combined organic phased were washed with brine, dried over sodium sulfate, filtered, and concentrated under reduced pressure to obtain compound **1** as a brownish oil (1.2 g, 6.33 mmol, 57%). The crude product was used for the next step without further purification. The experimental NMR data correspond with those from the literature.

^1^H NMR (500 MHz, CDCl_3_): δ 4.02 (s, 1H), 3.95 (s, 1H), 2.94 (s, 3H), 1.46 (d, *J* = 18.7 Hz, 9H).

Liquid chromatography–mass spectrometry (LC-MS) [electrospray ionization (ESI)]: mass/charge ratio (*m/z*) 190 (M + H^+^).

### *tert*-Butyl {2-[(benzo[*d*][1,3]dioxol-5-ylmethyl)amino]-2-oxoethyl}(methyl)carbamate (2)

To an ice-cooled and stirring solution of *N*-Boc-sarcosine **1** (1.20 g, 6.33 mmol, 1 equiv) in dry tetrahydrofuran (THF) (10 ml) was added triethylamine (2.54 ml, 1.36 g, 18.99 mmol, 3 equiv) and isobutyl chloroformate (0.95 g, 6.96 mmol, 1.2 equiv). The solution was allowed to come to r.t. and was stirred for 2 hours at r.t. under nitrogen atmosphere. Piperonylamine (0.76 ml, 0.95 g, 6.33 mmol, 1 equiv) was added, and the reaction mixture was stirred for 2 hours under the same conditions. The solution was quenched with an aqueous, saturated NaHCO_3_ solution (50 ml) and extracted with CH_2_Cl_2_ (3 x 40 ml). Combined organic phases were washed with brine (40 ml), dried over sodium sulfate, filtered, and concentrated under reduced pressure. The crude product was purified by column chromatography (1:20, MeOH:CH_2_Cl_2_) to afford compound **2** (1.71 g, 5.32 mmol, 84%).

*R*_f_ (Retention factor): 0.38 (1:50, MeOH:CH_2_Cl_2_).

^1^H NMR (500 MHz, CDCl_3_): δ 6.76 to 6.74 (m, 2H), 6.72 (dd, *J* = 7.9, 1.6 Hz, 1H), 5.94 (s, 2H), 4.37 (d, *J* = 5.7 Hz, 2H), 3.88 (s, 2H), 2.94 (s, 3H), 1.43 (s, 9H).

LC-MS (ESI): *m/z* 345 (M + Na).

### *N*-(Benzo[*d*][1,3]dioxol-5-ylmethyl)-2-(methylamino)acetamide (3)

Compound **2** (629 mg, 1.95 mmol, 1 equiv) was dissolved in CH_2_Cl_2_ (10 ml). Trifluoroacetic acid (TFA) (5 ml) was added, and the reaction mixture was stirred overnight at r.t. The solution was quenched with an aqueous, saturated NaHCO_3_ solution (50 ml) and extracted with CH_2_Cl_2_ (3 x 40 ml). Combined organic phases were washed with brine (40 ml), dried over sodium sulfate, filtered, and concentrated under reduced pressure to afford the compound **3** (311 mg, 1.40 mmol, 72%). The crude product was used for the next step without further purification.

^1^H NMR (500 MHz, CDCl_3_): δ 6.78 to 6.75 (m, 3H), 5.94 (s, 2H), 4.38 (d, *J* = 5.9 Hz, 2H), 3.93 (d, *J* = 6.7 Hz, 2H), 2.46 (s, 3H).

LC-MS (ESI): *m/z* 223 (M + H^+^).

### *N*-(benzo[*d*][1,3]dioxol-5-ylmethyl)-2-{[(5-bromothiophen-2-yl)methyl](methyl)amino}acetamide (C26)

To a stirring solution of compound **3** (202 mg, 0.90 mmol, 1 equiv) in dry THF (6 ml) was added 5-bromothiophene-2-carbaldehyde (83 μl, 146 mg, 0.76 mmol, 0.84 equiv) and acetic acid (104 μl, 109 mg, 1.80 μmol, 2 equiv). After 10 min, sodium triacetoxyborohydride (288 mg, 1.35 mmol, 1.5 equiv) was added and the solution was stirred for 18 hours at r.t. under nitrogen atmosphere. The reaction mixture was concentrated under reduced pressure, the crude product was purified by reversed-phase (RP) flash chromatography, and product-containing fractions were lyophilized. The residue was taken up in MeOH and purified by HPLC to afford compound **C26** (58 mg, 0.14 μmol, 20%).

^1^H NMR (500 MHz, DMSO-d_6_): δ 8.19 (t, *J* = 5.8 Hz, 1H), 7.06 (d, *J* = 3.7 Hz, 1H), 6.86 to 6.83 (m, 3H), 6.73 (dd, *J* = 8.1, 1.4 Hz, 1H), 5.97 (s, 2H), 4.20 (d, *J* = 6.1 Hz, 2H), 3.79 (s, 2H), 3.05 (s, 2H), 2.25 (s, 3H).

^13^C NMR (126 MHz, DMSO-d_6_): δ 169.1, 147.23, 147.21, 146.0, 133.4, 129.8, 127.0, 120.4, 110.5, 107.9, 100.8, 59.1, 55.5, 42.0, 41.6.

 High-Resolution Mass Spectrometry (HRMS) (ESI) calculated for C_16_H_18_BrN_2_O_3_S [M (^79^Br) + H^+^]: 397.0222, found: 397.0214; calculated for C_16_H_17_BrN_2_O_3_S [M (^81^Br) + H^+^]: 399.0201, found: 399.0194.

### *N*-(benzo[*d*][1,3]dioxol-5-ylmethyl)-2-[(2-bromobenzyl)(methyl)amino]acetamide (SW-C165)

To the compound **3** (35 mg, 0.157 mmol, 1.0 equiv) in THF (2 ml) was added 2-bromobenzaldehyde (29 mg, 0.157 mmol, 1.0 equiv) and acetic acid (19 μl, 0.314 mmol, 2.0 equiv) at r.t. under nitrogen. The mixture was stirred for 5 min, and sodium triacetoxyborohydride (50 mg, 0.236 mmol, 1.5 equiv) was added in one portion and stirred overnight at r.t. The reaction mixture was quenched with saturated NaHCO_3_ solution (30 ml) and extracted with Et_2_O (2 x 25 ml). The combined organic layers were dried over Na_2_SO_4_, filtered, and concentrated under reduced pressure. The crude compound was purified by column chromatography [1:2, Ethyl Acetate (EtOAc):Petroleum Ether (PE)] to obtain the compound **SW-C165** (37 mg, 61%) as a colorless oil.

*R*_f_: 0.5 [1:1, EtOAc:PE, stain: ultraviolet (UV)/KMnO_4_].

^1^H NMR (400 MHz, CDCl_3_): δ 7.56 (bs, 1H), 7.53 (d, *J* = 8.4 Hz, 1H), 7.22 to 7.27 (m, 2H), 7.10 to 7.18 (m, 1H), 6.73 (d, *J* = 7.9 Hz, 1H), 6.64 to 6.70 (m, 2H), 5.94 (s, 2H), 4.30 (d, *J* = 6.1 Hz, 2H), 3.65 (s, 2H), 3.12 (s, 2H), 2.31 (s, 3H).

^13^C NMR (101 MHz, CDCl_3_): δ 170.7, 148.0, 147.0, 137.2, 133.4, 132.3, 131.6, 129.4, 127.5, 125.2, 121.0, 108.4, 108.4, 101.1, 62.3, 60.5, 43.5, 42.9.

HRMS (ESI) calculated for C_18_H_20_BrN_2_O_3_ (M + H^+^): 391.0657, found: 391.0656.

### *N*-(benzo[*d*][1,3]dioxol-5-ylmethyl)-2-{[(5-bromofuran-2-yl)methyl](methyl)amino}acetamide (SW-C210)

The compound **3** (151.6 mg, 0.68 mmol, 1 equiv) was dissolved in THF (10 ml). 5-Bromo-2-furaldehyde (119 mg, 0.68 mmol, 1 equiv), acetic acid (78 μl, 80 mg, 1.36 mmol, 2 equiv), and sodium triacetoxyborohydride (216 mg, 1.02 mmol, 1.5 equiv) were added, and the yellowish reaction mixture was stirred for 16 hours at r.t. under nitrogen atmosphere. The reaction was quenched with saturated aqueous NaHCO_3_ solution (30 ml), and the aqueous phase was extracted with diethyl ether (3 x 50 ml). Combined organic phases were dried over sodium sulfate, filtered, and concentrated under reduced pressure. The crude product was purified by column chromatography (EtOAc:PE, 50:50 to 100:0) and HPLC afterward. Product-containing fractions were lyophilized yielding compound **SW-C210** as a white solid (72 mg, 0.18 mmol, 28%).

*R*_f_: 0.41 (1:1, PE:EtOAc).

^1^H NMR (500 MHz, MeOH-d_4_): δ 6.79 (dd, *J* = 1.3, 0.6 Hz, 1H), 6.76 (dd, *J* = 1.9, 1.1 Hz, 2H), 6.35 (q, *J* = 3.3 Hz, 2H), 5.91 (s, 2H), 4.30 (s, 2H), 3.73 (s, 2H), 3.21 (s, 2H), 2.39 (s, 3H).

^13^C NMR (126 MHz, MeOH-d_4_): δ 171.9, 154.2, 149.2, 148.3, 133.6, 122.8, 122.0, 113.8, 113.2, 109.1, 109.1, 102.3, 60.1, 54.2, 43.6, 42.8.

HRMS (ESI) calculated for C_16_H_18_BrN_2_O_4_ [M (^79^Br) + H^+^]: 381.0450, found: 381.0453; calculated for C_16_H_17_BrN_2_O_4_ [M (^81^Br) +H^+^]: 383.0429, found: 383.0438.

**Fig. 8.**
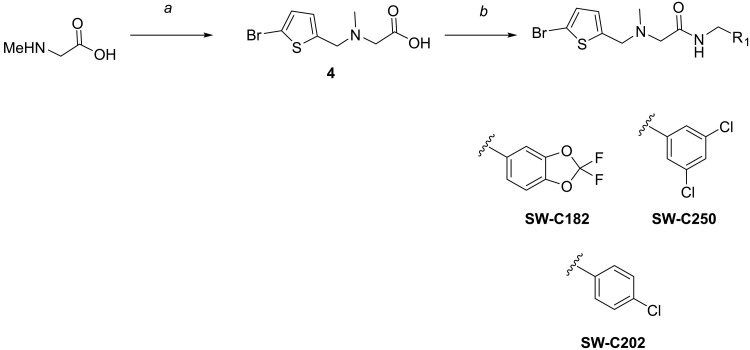
Reagents and conditions for the synthesis of SW-C182, SW-C202, and SW-C250. (**a**) 5-Bromothiophene-2-carbaldehyde, HOAc, THF, r.t., 10 min, then NaBH(OAc)_3_, r.t., overnight, 97%; (**b**) iBuOCOCl, Et_3_N, THF, 0°C to r.t., 2 hours, then R_1_CH_2_NH_2_, r.t., 2 hours; or R_1_CH_2_NH_2_, HATU, DIPEA, DMF.

Further C26 analogs were obtained according to the reaction scheme shown in Fig. 8.

### *N*-[(5-bromothiophen-2-yl)methyl]-*N*-methylglycine (4)

To a solution of sarcosine (200 mg, 2.24 mmol, 2 equiv) in dry MeOH (5 ml) was added triethylamine (313 μl, 227 mg, 2.24 mmol, 2 equiv) and 5-bromothiophene-2-carboxaldehyde (121 μl, 214 mg, 1.12 mmol, 1 equiv). The reaction mixture was stirred for 14 hours at r.t. under argon atmosphere, then NaBH_4_ (169 mg, 4.48 mmol, 4 equiv) was added, and the solution was stirred for another 16 hours at the same conditions. All volatile components were removed under reduced pressure, and the crude product was purified by RP flash chromatography to afford the compound **4** as a white solid (286 mg, 1.08 mmol, 97%).

^1^H NMR (500 MHz, MeOH-d_4_): δ 6.92 (d, *J* = 3.7 Hz, 1H), 6.77 (dt, *J* = 3.7, 0.8 Hz, 1H), 3.82 (d, *J* = 0.6 Hz, 2H), 3.03 (s, 2H), 2.32 (s, 3H).

^13^C NMR (126 MHz, MeOH-d_4_): δ 178.1, 145.0, 130.5, 128.2, 112.1, 61.2, 56.4, 42.4, 35.9.

HRMS (ESI) calculated for C_8_H_11_BrNO_2_S [M (^79^Br) + H^+^]: 263.9694, found: 263.9704; calculated for C_8_H_10_BrNO_2_S [M (^81^Br) + H^+^]: 265.9673, found: 265.9683.

### 2-{[(5-Bromothiophen-2-yl)methyl](methyl)amino}-*N*-[(2,2-difluorobenzo[*d*][1,3]dioxol-5-yl)methyl]acetamide (SW-C182)

To the compound **4** (30 mg, 0.113 mmol, 1.0 equiv) in THF (2 ml) was added triethylamine (80 μl, 0.567 mmol, 5.0 equiv) under nitrogen atmosphere at 0°C. Isobutyl chloroformate (23 μl, 0.175 mmol, 1.50 equiv) was added dropwise at 0°C and stirred for 2 hours at r.t., and (2,2-difluorobenzo[*d*][1,3]dioxol-5-yl)methanamine (21 mg, 0.113 mmol, 1.0 equiv) was added at r.t. in a single portion and stirred at r.t. for an additional 2 hours. The reaction mixture was quenched with saturated NaHCO_3_ solution (20 ml) and extracted with Et_2_O (2 x 20 ml). The combined organic layers were dried over anhydrous Na_2_SO_4_, filtered, and concentrated under vacuum. The crude compound was purified using column chromatography (2:3, EtOAc:PE) to obtain compound **SW-C182** (10 mg, 20%) as a colorless oil.

*R*_f_: 0.40 (1:2, PE:EtOAc, stain: KMnO_4_/UV).

^1^H NMR (400 MHz, CDCl_3_): δ 6.97 to 7.05 (m, 3H), 6.89 (d, *J* = 3.6 Hz, 1H), 6.71 (bs, 1H), 4.44 (d, *J* = 5.6 Hz, 2H), 3.80 (bs, 2H), 3.20 (bs, 2H), 2.40 (s, 3H).

^13^C NMR (101 MHz, CDCl_3_): δ 170.0, 144.1, 143.2, 134.7, 131.8, 129.8, 129.3, 123.0, 109.6, 109.3, 56.9, 42.9, 31.1.

HRMS (ESI) calculated for C_16_H_16_BrF_2_N_2_O_3_S (M + H^+^): 433.0033, found: 433.0029.

### 2-{[(5-Bromothiophen-2-yl)methyl](methyl)amino}-*N*-(3,5-dichlorobenzyl)acetamide (SW-C250)

To a stirring solution of compound **4** (100 mg, 379 μmol, 1 equiv) in dry DMF (6 ml) was added *N*,*N*-Diisopropylethylamine (DIPEA) (198 μl, 147 mg, 1140 μmol, 3 equiv) and Hexafluorophosphate Azabenzotriazole Tetramethyl Uronium (HATU) (173 mg, 454 μmol, 1.2 equiv). After 10 min, 3,4-dichlorobenzylamine (61 μl, 80 mg, 454 μmol, 1.2 equiv) was added and the solution was stirred for 20 hours at r.t. under argon atmosphere. The mixture was diluted with water (10 ml) and extracted with CH_2_Cl_2_ (3 x 30 ml). Phases were separated, and combined organic phases were washed with brine (20 ml) and dried over sodium sulfate. The solution was filtered, washed with CH_2_Cl_2_, and concentrated under reduced pressure. The residue was purified by RP flash chromatography and then HPLC. Product-containing fractions were lyophilized to yield compound **SW-C250** (28.5 mg, 18%).

^1^H NMR (500 MHz, CD_3_OD): δ 7.33 (t, *J* = 2.0 Hz, 1H), 7.27 (d, *J* = 2.0 Hz, 2H), 6.94 (d, *J* = 3.7 Hz, 1H), 6.79 (dt, *J* = 3.7, 0.9 Hz, 1H), 4.39 (s, 2H), 3.79 (d, *J* = 0.9 Hz, 2H), 3.10 (s, 2H), 2.36 (s, 3H).

^13^C NMR (126 MHz, CD_3_OD): δ 173.64, 145.03, 144.45, 136.29, 130.88, 128.43, 128.26, 127.33, 112.85, 60.33, 57.43, 43.48, 42.91.

HRMS (ESI) calculated for C_15_H_16_BrCl_2_N_2_OS [M (^79^Br) + H^+^]: 420.9544, found: 420.9548; calculated for C_15_H_16_BrCl_2_N_2_OS [M (^81^Br) + H^+^]: 422.9523, found: 422.9558.

### 2-{[(5-Bromothiophen-2-yl)methyl](methyl)amino}-*N*-(4-chlorobenzyl)acetamide (SW-C202)

To a stirring solution of compound **4** (200 mg, 0.75 mmol, 1 equiv) in dry DMF (6 ml) was added DIPEA (395 μl, 293 mg, 2.27 mmol, 3 equiv) and HATU (345 mg, 0.90 mmol, 2 equiv). After 10 min, 4-chlorobenzylamine (110 μl, 128 mg, 0.90 mmol, 1.2 equiv) was added and the solution was stirred for 3 hours at r.t. under nitrogen atmosphere. The reaction mixture was diluted with water (10 ml) and extracted with CH_2_Cl_2_ (3 x 30 ml). Combined organic phases were washed with brine (20 ml), dried over sodium sulfate, filtered, and concentrated under reduced pressure. The residue was taken up in DMSO and purified twice by HPLC. Product-containing fractions were lyophilized to yield compound **SW-C202** (50 mg, 128 μmol, 17%) as a yellow oil.

^1^H NMR (500 MHz, DMSO-d_6_): δ 8.41 (s, 1H), 7.39 to 7.36 (m, 2H), 7.29 to 7.26 (m, 2H), 7.10 (d, *J* = 3.3 Hz, 1H), 6.90 (s, br, 1H), 4.29 (d, *J* = 6.1 Hz, 2H), 3.90 (s, 2H), 3.19 (s, 2H), 2.34 (s, 3H).

^13^C NMR (176 MHz, DMSO-d_6_): δ 158.1, 140.4, 138.5, 131.3, 130.8, 129.9, 129.1, 128.8, 128.2, 127.9, 55.2, 42.9, 35.8.

HRMS (ESI) calculated for C_15_H_17_BrClN_2_OS [M (^79^Br) + H^+^]: 386.9933, found: 386.9954; calculated for C_15_H_16_BrClN_2_OS [M (^81^Br) +H^+^]: 388.9913, found: 388.9905.

**Fig. 9.**
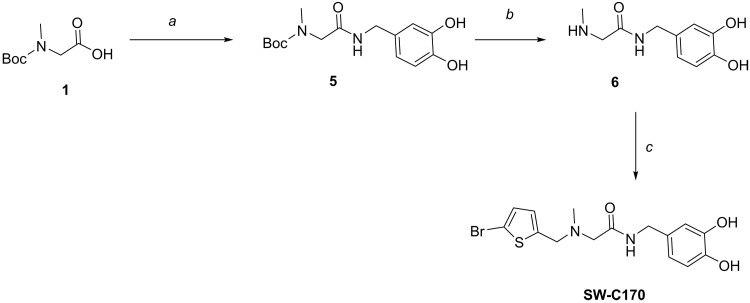
Reagents and conditions for the synthesis of SW-C170. (**a**) iBuOCOCl, Et_3_N, THF, 0°C to r.t., 2 hours, then (HO)_2_C_6_H_3_CH_2_NH_2_, r.t., 2 hours, 42%; (**b**) TFA, CH_2_Cl_2_, r.t., 5 hours; (**c**) 2-bromo-5-(bromomethyl)thiophene, Et_3_N, THF, 16 hours, 68% over two steps.

2-{[(5-Bromothiophen-2-yl)methyl](methyl)amino}-*N*-(3,4-dihydroxybenzyl)acetamide (**SW-C170**) was synthesized according to the reaction scheme shown in Fig. 9.

### *tert*-Butyl {2-[(3,4-dihydroxybenzyl)amino]-2-oxoethyl}(methyl)carbamate (5)

To the Boc-sarcosine **1** (0.270 g, 1.440 mmol, 1.0 equiv) in THF (10 ml) was added triethylamine (0.80 ml, 5.750 mmol, 4.0 equiv) under nitrogen atmosphere at 0°C. Isobutyl chloroformate (0.210 ml, 1.580 mmol, 1.10 equiv) was added dropwise at 0°C and stirred for 2 hours min at r.t., and 3,4-dihydroxybenzylamine (0.200 g, 1.440 mmol, 1.0 equiv) was added at r.t. in a single portion and stirred at r.t. for 16 hours. The reaction mixture was quenched with saturated NaHCO_3_ solution (20 ml) and extracted with EtOAc (2 x 20 ml). The combined organic layers are dried over anhydrous Na_2_SO_4_, filtered, and concentrated under reduced pressure. The crude compound was purified using column chromatography (1:20, MeOH:CH_2_Cl_2_) to obtain compound **5** (0.189 g, 42%) as a colorless liquid.

*R*_f_: 0.30 (1:20, MeOH:CH_2_Cl_2_, stain: ninhydrin/UV).

^1^H NMR (400 MHz, CD_3_OD): δ 6.59 to 7.03 (m, 3H), 4.30 (s, 2H), 3.96 (m, 2H), 2.95 (s, 3H), 1.36 (m, 9H).

^13^C NMR (101 MHz, CD_3_OD): δ 171.4, 157.1, 146.4, 145.7, 120.4, 120.1, 116.2, 81.6, 53.4, 43.8, 36.3, 28.5.

HRMS (ESI) calculated for C_15_H_22_N_2_O_5_Na (M + Na^+^): 333.1426, found: 333.1426.

### *N*-(3,4-Dihydroxybenzyl)-2-(methylamino)acetamide (6)

To the compound **5** (50 mg, 0.160 mmol, 1.0 equiv) in CH_2_Cl_2_ (2 ml) was added TFA (0.7 ml) at r.t. and stirred for 5 hours. The reaction mixture was concentrated under reduced pressure to afford crude **6** that was used without further characterization.

### 2-{[(5-Bromothiophen-2-yl)methyl](methyl)amino}-*N*-(3,4-dihydroxybenzyl)acetamide (SW-C170)

2-Bromo-5-(bromomethyl)thiophene (40 mg, 0.17 mmol, 1.0 equiv) was dissolved in dry THF (0.5 ml), triethylamine (242 μl, 1.68 mmol, 10.0 equiv) was added, and the solution was cooled to 0°C. Compound **6** (54 mg, 0.16 mmol, 1.00 equiv) was added, and the mixture was stirred 5 min at 0°C and at r.t. for 16 hours. THF was removed under reduced pressure. The crude mixture was washed with sat. NaHCO_3_ solution (10 ml) and extracted with ethyl acetate (3 x 10 ml). The combined organic layers were dried over anhydrous Na_2_SO_4_, filtrated, and evaporated under reduced pressure. The crude product was purified by column chromatography (1:20, MeOH:CH_2_Cl_2_) to obtain product **SW-C170** (44 mg, 68%) as a yellow oil.

*R*_f_: 0.8 (1:20, MeOH:CH_2_Cl_2_, stain: KMnO_4_/UV).

^1^H NMR (400 MHz, CD_3_OD): δ 6.92 (d, *J* = 3.7 Hz, 1H), 6.73 (dd, *J* = 8.2, 6.0 Hz, 3H), 6.61 (dd, *J* = 8.1, 2.0 Hz, 1H), 4.26 (s, 2H), 3.76 (s, 2H), 3.07 (s, 2H), 2.32 (s, 3H).

^13^C NMR (101 MHz, CD_3_OD): δ 172.7, 146.4, 145.7, 144.8, 131.3, 130.7, 128.2, 120.2, 116.3, 116.0, 112.6, 60.4, 57.2, 43.6, 43.1.

### In vitro cell toxicity assays

In vitro toxicity of compound C26 was assessed in HeLa cells (ATCC CCL-2) using the ApoTox-Glo Triplex Assay and in macrophages RAW 264.7-LgBiT using the CellTox Green Cytotoxicity Assay (Promega, USA) according to the manufacturer’s instructions. The screening of in vitro toxicity of the compound analogs was performed with the CellTox Green Cytotoxicity Assay (Promega, USA) according to the manufacturer’s instructions. Cells (10^4^ cells per well) were seeded into a 96-well plate and incubated for 24 hours at 37°C, 5% (v/v) CO_2_. Cells were then treated with the compound at different concentrations or 1% (v/v) DMSO in Dulbecco’s modified Eagle’s medium (DMEM) without phenol red at the desired exposure time. Fluorescence intensity was measured with a TECAN Spark microplate reader. Cytotoxicity [relative fluorescence units (RFU)] and viability (RFU) values were plotted to calculate the TC_50_ using GraphPad Prism 10.1.1.

### Toxicology studies

C26 was formulated in 5% Tween 20 (v/v), 50% lecithin (from soy bean: 40 mg in 800 μl of distilled water), and 45% PBS at 0.6, 2, and 6 mg/ml for oral administration. A dosing volume of 5 ml/kg was applied. C26 was administered orally to groups of three male Institute of Cancer Research (ICR) mice (23 ± 3 g) at an initiating dose of 3 mg/kg in an MTD setup. The animals received an initial dose of 3 mg/kg. If the animals survived for 72 hours, the group (10 mg/kg) was tested. If the animals survived for 72 additional hours, the group (30 mg/kg) was tested. Experiments were performed by Pharmacology Discovery Services Taiwan Ltd., in general accordance with the “Guide for the Care and Use of Laboratory Animals: Eighth Edition” (National Academies Press, Washington, DC, 2011). The animal care and protocol were reviewed and approved by the Institutional Animal Care and Use Committee (IACUC) at Pharmacology Discovery Services Taiwan Ltd.

### SipA injection assay

SipA injection assay was performed using the split-Nanoluc (HiBiT/LgBiT) system as described previously (*37*, *38*). In brief, NanoLuc is split into two parts: LgBiT, comprising 10 of the 11 β strands of the luciferase, and HiBiT, a short peptide with high affinity to LgBiT, contributing the missing β strand to make a functional luciferase. *S.* Typhimurium strains expressing SipA-HiBiT were grown in LB supplemented with 0.3 M NaCl and either compound at different concentrations or 1% (v/v) DMSO. Cultures were incubated at 37°C with shaking at 180 rpm until an OD_600_ of 0.9 was reached. Bacteria were then pelleted and washed twice with Hanks’ balanced salt solution (HBSS). The bacterial suspension was diluted to obtain a multiplicity of infection (MOI) of 50 and then used to infect the LgBiT-expressing HeLa cell. Luminescence was measured using a Tecan Sparc Multimode reader.

### Plasmid-based SipA secretion assay

The plasmid pT10-SipA-NLuc was electroporated into electrocompetent cells of *S. enterica* clinical isolates. Bacteria at an initial OD_600_ of 0.02 were grown for 5 hours in 1 ml of LB supplemented with 0.3 M NaCl, kanamycin (50 μg/ml), and either compound at 100 μM or 1% (v/v) DMSO. Cultures were pelleted, and then 25 μl of the supernatant was transferred to a 384-well plate to measure luminescence as previously described.

### SiiE cell surface retention assay

HiBiT was inserted chromosomally into SiiE at position K5411, using the suicide plasmid pMIB8021 (pSB890-*siiE*::K5411HiBiT). Bacteria were grown for 5 hours under SPI-4–inducing conditions (LB supplemented with 0.3 M NaCl). OD units of 0.5 were harvested at 10,000*g* for 2 min at 4°C. Cell pellets were washed twice with cold PBS and then resuspended to a final concentration of 0.5 OD units. Twenty-five microliters from the bacterial suspension was transferred into a 384-well plate. The Nano-Glo HiBiT Extracellular Buffer and corresponding substrate were prepared according to the manufacturer’s instructions. Twenty-five microliters of the Nano-Glo HiBiT Extracellular buffer-substrate mix was added to each sample and then incubated at r.t. for 10 min. Luminescence was measured with a Tecan Spark microplate reader.

### Invasion assay into HeLa cells

In a white 24-well plate (NUNC), 10^5^ HeLa cells were seeded in 350 μl of DMEM (Gibco), 24 hours before the infection. Bacteria were grown in LB supplemented with 0.3 M NaCl and either compound at different concentrations or 1% (v/v) DMSO, at 37°C with shaking at 180 rpm until an OD_600_ of 0.9 was reached. Bacteria were then pelleted and washed twice with HBSS. The bacterial suspension was diluted to obtain an MOI of 20 to infect HeLa cells. The plate was centrifuged for 5 min at 300*g* to synchronize the infection and then incubated for 25 min at 37°C and 5% (v/v) CO_2_ to allow *Salmonella* invasion into HeLa cells. To quantify the invasiveness, the cells were first washed three times with 500 μl of prewarmed PBS. The remaining extracellular bacteria were killed with 500 μl of DMEM supplemented with gentamicin (100 μg/ml). HeLa cells were incubated for 1 hour and then washed three times with prewarmed PBS and lysed with 500 μl of 0.5% (v/v) SDS in PBS for 5 min at 37°C on a shaking platform. The lysate was serial diluted in PBS-T [PBS with 0.05% (v/v) Tween 20] to determine the colony-forming units (CFUs) by plating on LB medium supplemented with streptomycin (50 μg/ml).

### Invasion assay into MDCK cells

MDCK (NBL-2) cells were seeded in a 24-well plate at a density of 10^5^ cells per well in 1 ml of MEM (Gibco) and grown for 5 to 6 days, to allow polarization. Cells were washed every 2 days with fresh medium. After 5 days, each well contained ~1.8 × 10^6^ MDCK cells. Bacteria were grown as for the invasion assay of HeLa cells and diluted to obtain an MOI of 5. The plate was centrifuged for 3 min at 300*g* to synchronize the infection and then incubated for 25 min at 37°C and 5% (v/v) CO_2_ to allow *Salmonella* invasion into MDCK cells. The quantification of invasiveness was carried out as described above for HeLa cells.

### SseF injection assay

SseF injection assay was performed using the split-Nanoluc (HiBiT/LgBiT) system as described for SipA. In a white 96-well plate (NUNC) with optical bottom, 10^4^ LgBiT-expressing RAW 264.7 macrophages (*65*) were seeded in 100 μl of DMEM, 24 hours before the infection. The strain harboring the plasmid-encoded *sseF*-HiBiT and the isogenic strain Δ*ssaV* were grown in LB-NaCl at 37°C at 180 rpm until an OD_600_ of 0.9. Bacteria were washed twice with HBSS (Serva) and then used to infect preseeded cells on a 96-well plate with an MOI of 10. The plate was centrifuged at 300*g* for 3 min and then incubated for 30 min to allow invasion. Afterward, macrophages were washed with DMEM and incubated for 1 hour in DMEM supplemented with gentamicin (100 μg/ml) to eliminate noninvading bacteria. The medium was replaced by DMEM with gentamicin (16 μg/ml), supplemented with either DMSO (1%) or C26 at different concentrations. Cells were incubated at 37°C with 5% (v/v) CO_2_ for 5.5 hours (total infection time of 7 hours) and then washed twice with 1x PBS before luminescence measurements were carried out following the manufacturer’s instructions.

### Western blotting analysis

Western blot analysis to quantify secreted and nonsecreted proteins was carried out as previously described (*66*). After a 5-hour culture under SPI-1–inducing conditions, cultures were centrifuged at 10,000*g* for 2 min at 4°C to separate cell pellets for the quantification of target proteins in whole cells, and the supernatant for the quantification of secreted proteins. The supernatants were first filtered with a 0.22-μm pore size filter. Sodium deoxycholic acid was then added to a final concentration of 0.1% (w/v) followed by a protein precipitation with 10% trichloroacetic acid (v/v) for 30 min at 4°C. The samples were pelleted by centrifugation at 20,000*g* for 20 min at 4°C to retrieve precipitated proteins, which were lastly washed with acetone before resuspension in SDS–polyacrylamide gel electrophoresis (PAGE) loading buffer. Whole-cell samples were directly resuspended in SDS-PAGE loading buffer.

### Transcriptome analysis

*Salmonella* was grown for 5 hours at 37°C in LB medium in the presence of 100 μM C26 or 1% (v/v) DMSO as a control. After growth, bacteria were harvested by centrifugation, and total RNA was isolated using the Qiagen RNeasy mini kit according to the manufacturer’s protocol. RNA-seq was performed on a HiSeq2500 with a 2 x 125–base pair paired-end read protocol. The Illumina Casava software was used to demultiplex the sequenced reads providing individual raw fastq sample files. Raw fastq files were prefiltered using the chastity filter to remove reads that contain a “Y” flag. FastQC (https://www.bioinformatics.babraham.ac.uk/projects/fastqc/, version v0.11.4) was used to determine the quality of the resulting fastq files. Subsequently, an adapter trimming/removal step was conducted with Cutadapt (https://pypi.python.org/pypi/cutadapt/, version 1.8.3). This process used FastQC output (see step before) to identify reads that showed a match to some typical overrepresented (Illumina) sequences/adapters. TopHat2 (https://ccb.jhu.edu/software/tophat/index.shtml, version v2.0.12) was used as aligner to map the remaining quality controlled reads to the Salmonella genome. Read counting to features (e.g., genes or exons) in the genome was performed with HTSeq-count (https://htseq.readthedocs.io/en/master/htseqcount.html, version 0.6.0.). Counting was performed using “union” mode on the feature “gene.” The stranded option was also set to “–stranded=no” to indicate to count features on both strands. Furthermore, the -r parameter from HTSeq was set to “pos” as this is the default of the output of TopHat’s “accepted_hits.bam” mapping file. For differential expression analysis, the raw read count table resulting from HTSeq counting is used and fed into the R package DESeq2 (version 1.10.1). Graphs were also produced in the R language (R version 3.2.1) mainly using the R package ggplot2 (version 2.2.0). Reports were produced using the R package rmarkdown (version 1.3).

### Whole-cell assay monitoring HilD activity

A whole-cell reporter gene assay was used to quantify HilD transcriptional activity. Strain P*_hilA_*-sfGFP and the isogenic Δ*hilD* P*_hilA_*-sfGFP were used for this purpose. Cultures were performed in 1 ml of LB supplemented with 0.3 M NaCl. Compounds at different concentrations or 1% (v/v) DMSO were added to the corresponding tubes. Cultures were incubated at 37°C and 180 rpm for 5 hours. Cells were pelleted, resuspended in 100 μl of PBS, and then transferred into a 96-well black clear-bottom plate (Thermo Fisher Scientific, USA). Fluorescence intensity was measured with a Tecan Spark microplate reader with an excitation wavelength of 485 nm and an emission wavelength of 510 nm. Dose-response curves and IC_50_ values were calculated using CDD Vault.

### Alanine scan

A plasmid-based assay in a ΔSPI-1 background strain was developed to facilitate the introduction of point mutations in *hilD*. The Rha cassette (*rhaS*, *rhaR*, and P*rha*) was first deleted from the pT10 backbone (*67*). The fragment P*_hilD_*-*hilD*-P*_hilA_*-sfGFP was then inserted upstream of the terminator *rrnB*. *hilD* was then deleted from the resulting plasmid to serve as a negative control. Site-directed mutagenesis was performed using KOD polymerase (Novagen). The cell-based fluorescence assay was performed as described above. Statistical analysis was performed by applying a first Bonferroni’s multiple comparisons test to compare treated and untreated conditions within each strain (shown in Fig. 4E) and a second same test to compare the treated mutants to the treated WT condition. The absence of significance in both tests corresponds to mutations inactivating HilD. Significance in the first but not the second test corresponds to a phenotype similar to that of the WT. Significance in the second but not first test corresponds to a full loss of sensitivity to C26. Significance in both tests corresponds to a partial loss of sensitivity to C26.

### Identification of the most frequent HilD mutations among *S. enterica*

The retrieved sequences and script used to determine the most frequent point mutations in HilD can be accessed on the Zenodo repository. Site-directed mutagenesis was performed using KOD polymerase (Novagen). P*_hilA_* activation was monitored as described above using the plasmid-based whole-cell fluorescence assay.

### Molecular modeling for HilD’s binding site prediction and C26’s binding mode suggestion

Molecular modeling of the HilD target and complete protocol for MD simulations are described below.

#### Protein structure prediction and binding site prediction

The structural model of the N-terminal truncated Salty HilD (UniProt ID: P0CL08, starting at Ser^37^) was retrieved from the AlphaFold Protein Structure Database (*68*). All structure models can be found in the Supplementary Materials. System preparation and docking calculations were performed using the Schrödinger Drug Discovery suite for molecular modeling (version 2022.1). The protein-ligand complex was prepared with the Protein Preparation Wizard to fix protonation states of amino acids, add hydrogens, and fix missing side-chain atoms, where we selected the most likely ionization state as proposed by the software, and the structures were minimized. Now, DNA binding interactions associated with the CTD of other AraC-like proteins’ CTD have been inferred from static models based on similar MarA and Rob proteins (*69*–*72*). However, there are no structural studies focused on the full-length HilD protein regarding how the NTD and CTD interact with each other and how potential ligands interfere with this geometry. In this sense, for each system, namely, monomer (M), monomers with DNA (MDNA) systems were generated. HilD+DNA was generated using the coordinates from the CTD with bound DNA modeled based on the MarA-DNA structure [PDB ID: 1BL0 (*71*); resolution: 2.3 Å] followed by energy minimization. Potential binding pockets were predicted using SiteMap (*73*).

#### Molecular docking

All ligands for docking were drawn using Maestro and prepared using LigPrep (*56*) to generate the 3D conformation, adjust the protonation state to physiological pH (7.4), and calculate the partial atomic charges with the OPLS4 force field (*57*). Docking studies with the prepared ligands were performed using Glide (Glide V7.7) (*58*, *59*) with the flexible modality of induced-fit docking with XP, followed by a side-chain minimization step using Prime. Ligands were docked within a grid around 12 Å from the centroid of the predicted binding site pocket, as determined using SiteMap.

#### MD simulation

MD simulations were carried out using Desmond with the OPLS4 force field (*57*). The simulated system encompassed the protein-ligand complexes, a predefined water model [TIP3P (*74*)] as a solvent, and counterions. The system was treated in a cubic box with periodic boundary conditions specifying the box’s shape and size as 13-Å distance from the box edges to any atom of the protein. In all simulations, we used a time step of 1 fs, and the short-range coulombic interactions were treated using a cutoff value of 9.0 Å using the short-range method, whereas the smooth particle mesh Ewald method handled long-range coulombic interactions (*75*). Initially, the system’s relaxation was performed using steepest descent and the limited-memory Broyden-Fletcher-Goldfarb-Shanno algorithms in a hybrid manner, according to the established protocol available in the Desmond standard settings. During the equilibration step, the simulation was performed under the Number of particles, Pressure, and Temperature (NPT) ensemble for 5 ns, implementing the Berendsen thermostat and barostat methods (*76*). A constant temperature of 310 K was kept throughout the simulation using the Nosé-Hoover thermostat algorithm (*77*) and Martyna-Tobias-Klein barostat (*78*) algorithm to maintain 1 atm of pressure. After minimization and relaxation of the system, we continued with the production step of at least 2 μs, with frames being recorded/saved every 1000 ps. Five independent replicas were produced for each compound, resulting in a total of ~10 μs simulation per ligand. Trajectories and interaction data are available on the Zenodo repository (*79*). The representative structures were selected by inspecting changes in the RMSD, meaning, for figures, a representative frame was selected at random at points of the trajectory where the RMSDs were not fluctuating, after equilibration. Figure S7 represents the variation of the RMSD values along with the simulation, for both template crystal structures and simulations with docking pose. In addition, the changes in the root mean square fluctuation (RMSF), normalized by residue for the protein backbone, are displayed in fig. S8.

##### MM-GBSA binding energy calculations.

MM-GBSA predicts the binding free energy of the protein-ligand complexes and the ranking of ligands based on the free energy could be correlated to the experimental binding affinities, especially in a congeneric series. Every 50th frame from the simulations was considered for the calculations. These were used as input files for the MM-GBSA calculations with thermal_mmgbsa.py script from the Schrödinger package, using Prime (*80*). Calculated free-binding energies are represented by the MM/GBSA and normalized by the number of heavy atoms (HAC), according to the following formula: ligand efficiency = (Binding energy)/[1 + ln(HAC)] and is expressed in kcal/mol·HAC, where HAC is the heavy atom count. Trajectory distances between specific secondary structure elements were calculated using their centers of mass with the Maestro script trj_asl_distance.py (Schrödinger LLC), using the carbon alpha coordinate of specific amino acids as a reference. Energy distribution is depicted in fig. S6.

### Recombinant protein expression and purification

The *hilC* gene was inserted into the pET-21a(+) vector, with an N-terminal His_6_ tag followed by a tobacco etch virus (TEV) protease cleavage site. The *hilD* gene was cloned into the pET-24a(+) vector, with an N-terminal His_6_-small ubiquitin-like modifier (SUMO) fusion. HilD mutants were cloned into the *hilD* construct by site-directed mutagenesis. Proteins were expressed in *E. coli* C41(DE3) (*81*) cells using lysogeny broth (LB) medium. An overnight culture was inoculated into LB medium, grown at 37°C until an OD_600_ of 0.6 to 0.8 was reached and induced by the addition of 0.5 mM isopropyl-β-d-thiogalactopyranoside (IPTG). Cells were incubated with shaking overnight at 25°C, collected by centrifugation (11,800*g*, 4°C) and resuspended in buffer A [50 mM NaH_2_PO_4_ (pH 7.0), 300 mM NaCl, and 10 mM imidazole] supplemented with DNase and one cOmplete EDTA-free protease inhibitor cocktail tablet (Roche, no. 11873580001). Cells were lysed using a French press [2x, 16,000 psi (1.1 × 10⁸ Pa)], and cell debris was removed by centrifugation (95,000*g*, 1 hour, 4°C). The supernatant was filtered (0.40 μm) and loaded to a Ni-NTA column. Bound proteins were washed first with 20% (v/v) buffer B [50 mM NaH_2_PO_4_ (pH 7.0), 300 mM NaCl, and 250 mM imidazole] and then eluted with 100% (v/v) of it. The eluted SUMO-HilD protein was supplemented with SUMO protease (250 μg) to cleave the His_6_-SUMO tag and dialyzed overnight at r.t. against buffer A. For HilC, the eluted protein was supplemented with TEV protease (1 mg) and dialyzed overnight at 6°C against buffer C [50 mM NaH_2_PO_4_ (pH 7.0) and 400 mM NaCl]. The dialyzed protein was reapplied to the Ni-NTA column and equilibrated with buffer A, and the column was washed with 25% (v/v) buffer B to elute the cleaved protein. In the case of HilC, a higher NaCl concentration of 500 mM was used for both Ni-NTA purification steps. Proteins were then concentrated using Amicon Ultra Centrifugal Filters and loaded to a SEC column (Superdex 75 26/60) equilibrated with SEC buffer [50 mM NaH_2_PO_4_ (pH 7.0) and 200 mM NaCl]. Eluted fractions containing a purified protein were combined, concentrated, and stored in aliquots at −80°C. Protein purity was assessed by SDS-PAGE, and protein concentration was determined from UV absorbance at 280 nm, measured using a NanoPhotometer NP80 (IMPLEN).

### Electrophoretic mobility shift assays

EMSAs were performed similarly to as described previously (*39*) using a 62–base pair double-stranded (dsDNA) fragment of the *hilA* promoter, encompassing the A1 binding site (*82*). dsDNA fragments were generated by melting the complementary primers PhilA_A1_f / PhilA_A1_r (table S10) together in TE buffer [10 mM Tris (pH 8.0) and 1 mM EDTA] at 95°C for 10 min before slowly cooling to r.t. The forward primer was modified with a 5′-Cy5 fluorescent dye for detection. The protein (600 nM) was incubated with 50 nM labeled DNA in EMSA buffer [20 mM Tris (pH 8.0), 100 mM KCl, 100 μM EDTA, and 3% glycerol]. C26 was diluted first in DMSO and subsequently 1:100 into the protein-DNA sample. Samples were incubated at 37°C for 15 min, supplemented with diluted DNA loading dye, and separated on a 1.5-mm-thick, 6% (w/v) TBE polyacrylamide gel at 6°C at a constant voltage of 100 V. Gels were imaged using a ChemiDocMP imaging system (Bio-Rad).

### Nanoscale differential scanning fluorimetry

Thermal stability of proteins was determined using nanoDSF, with runs performed on a Prometheus NT.48 (NanoTemper Technologies). A twofold serial dilution series of C26 was prepared in DMSO. C26 was added to HilD or HilC (5 μM) in SEC buffer, giving a final DMSO concentration of 1% (v/v). Samples were incubated for >20 min at r.t. and centrifuged for 2 min prior to loading of standard capillaries (no. PR-C002). Samples were heated from 20° to 80°C with a temperature gradient of 0.5°C min^−1^. Melting temperatures were calculated from changes in the fluorescence ratio (350/330 nm), using PR.Stability Analysis v1.0.3 and a temperature range of 40° to 70°C for curve fitting. Data analysis was performed using Prism 8.4 (GraphPad). The change in HilD melting temperature, *T*_m_, was fitted as a function of ligand concentration, using Eqs. 1 and 2 to yield apparent affinity (*K*_d,app_) values

$$T_{m}\left\{{\left[L\right]}_{0}\right\}=T_{m,\text{lower}}+\left(T_{m,\text{upper}}-T_{m,\text{lower}}\right)*\left(1-\alpha \left\{{\left[L\right]}_{0}\right\}\right)$$ (1)

$$\begin{array}{c} \alpha \left\{{\left[L\right]}_{0}\right\}=\\ \frac{{\left[P\right]}_{t}-K_{d}-{\left[L\right]}_{t}+\sqrt{{\left\{{\left[P\right]}_{t}+{\left[L\right]}_{t}+K_{d,\text{app}}\right\}}^{2}-\{4{\left[P\right]}_{t}{\left[L\right]}_{t}\}}}{2{\left[P\right]}_{t}} \end{array}$$ (2)

where [P]_t_ and [L]_t_ are the total protein and ligand concentrations, respectively.

### Size exclusion chromatography–multiangle light scattering

SEC-MALS experiments were performed using a Superdex 75 Increase 10/300 GL column (Cytiva) coupled to a miniDAWN Tristar Laser photometer (Wyatt) and a RI-2031 differential refractometer (JASCO). HilD (100 μM) was incubated with 1% (v/v) of either DMSO or C26 (10 mM dissolved in DMSO) for 20 min at r.t. Fifty microliters of HilD samples was loaded onto the SEC column, equilibrated with SEC buffer, and separated using a flow rate of 0.5 ml min^−1^. Data analysis was carried out with the ASTRA v7.3.0.18 software (Wyatt).

### BS^3^ cross-linking

BS^3^ cross-linking of HilD was performed as previously described (*39*). In summary, HilD (10 μM) was first incubated with C26 or oleic acid in SEC buffer for 20 min at r.t., with a final DMSO concentration of 1% (v/v). HilD was then cross-linked by incubation with 0.2 mM BS^3^ (Thermo Fisher Scientific, Pierce, A39266) at r.t. for 1 hour, before the reaction was quenched by the addition of 50 mM Tris (pH 7.5). Samples were analyzed using SDS-PAGE and visualized by silver staining.

### NMR spectroscopy

Assignment of the C26 spectrum was readily available from considerations of chemical shifts and ^3^J couplings in a 1D spectrum. STD experiments (*83*) were acquired on the HilD C26 complex using a ligand concentration of 60 μM and a protein concentration of 16 μM.Measurements were carried out at 800 MHz on a Bruker AVIII spectrometer. Spectra were acquired at 298 K with 4096 scans and 16,384 acquired data points, with saturation at 0.8 parts per million (ppm) targeting protein methyl groups. An STD buildup series was acquired using saturation times of 400, 800, 1200, 2000, and 3000 μs.

The expected STD intensities were backcalculated for frames of the MD trajectories using the CORCEMA algorithm (*40*–*42*) implemented within the SHINE NOESY backcalculation suite (in-house software). This implementation uses ligand chemical shift and coupling data (fig. S8) plus estimated of linewidth to simulate the STD spectrum. This allows direct comparison of experimental and backcalculated spectra with an *R*-factor based on the RMSD (*84*). Parameters provided to the program include the protein and ligand concentrations, ligand affinity (35 μM), an estimate of the protein correlation time (12 ns), ligand chemical shifts, and couplings. The noninstantaneous saturation model was used with saturated protons selected on the basis of chemical shifts predicted for the HilD AlphaFold model using SHIFTX2 (*85*). Quantitative comparison was carried out on the aromatic region of the spectrum (5.5 to 7.2 ppm) as these signals are well separated from residual protein and buffer signals and their intensities are expected to be most sensitive to the orientation of the ligand ring systems.

### Hydrogen-deuterium exchange mass spectrometry

HDX-MS experiments on HilD were conducted similar as described previously (*39*). HDX-MS was performed on two samples of HilD, i.e., without or with C26 present. To do so, HilD (25 μM) was supplemented with 1% (v/v) of either DMSO or C26 (10 mM dissolved in DMSO), yielding a final concentration of 100 μM C26 in the sample. Both samples were stored in a cooled tray (1°C) until measurement.

HDX reactions were prepared by a two-arm robotic autosampler (LEAP Technologies) by addition of 67.5 μl of HDX buffer [50 mM sodium phosphate (pH 7.0), 200 mM NaCl, and 1% (v/v) DMSO] prepared with 99.9% D_2_O to 7.5 μl of HilD samples without C26. HDX reactions of HilD in the presence of C26 were prepared similarly, but the HDX buffer was supplemented with 100 μM C26 to prevent dilution of the compound upon addition of HDX buffer. After incubation at 25°C for 10, 30, 100, 1000, or 10,000 s, 55 μl of the HDX reaction was withdrawn and added to 55 μl of predispensed quench buffer [400 mM KH_2_PO_4_/H_3_PO_4_ (pH 2.2) and 2 M guanidine-HCl] kept at 1°C. Ninety-five microliters of the resulting mixture was injected into an ACQUITY UPLC M-Class System with HDX Technology (Waters) (*86*). Nondeuterated protein samples were prepared similarly (incubation for ~10 s at 25°C) by 10-fold dilution of HilD samples with H_2_O-containing HDX buffer. The injected samples were flushed out of the loop (50 μl) with H_2_O + 0.1% (v/v) formic acid (100 μl min^−1^) and guided to a protease column (2 mm by 2 cm) containing the below specified proteases immobilized to the bead material, which was kept at 12°C. For each protein state and time point, three replicates (individual HDX reactions) were digested with porcine pepsin, whereas another three replicates were digested with a column filled with a 1:1 mixture of protease type XVIII from *Rhizopus* spp. and protease type XIII from *Aspergillus saitoi*. In both cases, the resulting peptides were trapped on an AQUITY UPLC BEH C18 1.7 μm 2.1 x 5 mm VanGuard pre-column (Waters) kept at 0.5°C. After 3 min of digestion and trapping, the trap column was placed in line with an ACQUITY UPLC BEH C18 1.7 μm 1.0 x 100 mm column (Waters), and the peptides were eluted at 0.5°C using a gradient of eluents A [H_2_O + 0.1% (v/v) formic acid] and B [acetonitrile + 0.1% (v/v) formic acid] at a flow rate of 30 μl min^−1^ as follows: 0 to 7 min: 95 to 65% A; 7 to 8 min: 65 to 15% A; 8 to 10 min: 15% A; 10 to 11 min: 5% A; and 11 to 16 min: 95% A. The eluted proteins were guided to a G2-Si HDMS mass spectrometer with ion mobility separation (Waters), peptides were ionized with an ESI source (250°C capillary temperature and spray voltage of 3.0 kV), and mass spectra were acquired in positive ion mode over a range of 50 to 2000 *m/z* in enhanced high-definition MS (HDMS^E^) or high-definition MS (HDMS) mode for nondeuterated and deuterated samples, respectively (*87*, *88*). [Glu1]-Fibrinopeptide B standard (Waters) was used for lock-mass correction. During separation of the peptide mixtures on the ACQUITY UPLC BEH C18 column, the protease column was washed three times with 80 μl of wash solution [0.5 M guanidine hydrochloride in 4% (v/v) acetonitrile], and blank injections were performed between each sample to reduce peptide carryover.

Peptide identification and analysis of deuterium incorporation were carried out with the ProteinLynx Global SERVER (PLGS, Waters) and DynamX 3.0 software (Waters) as described previously (*39*). In summary, peptides were identified with PLGS from the nondeuterated samples acquired with HDMS^E^ by using low energy, elevated energy, and intensity thresholds of 300, 100, and 1000 counts, respectively. Identified ions were matched to peptides with a database containing the amino acid sequence of HilD, porcine pepsin, and their reversed sequences with the following search parameters: peptide tolerance = automatic; fragment tolerance = automatic; min fragment ion matches per peptide = 1; min fragment ion matches per protein = 7; min peptide matches per protein = 3; maximum hits to return = 20; maximum protein mass = 250,000; primary digest reagent = nonspecific; missed cleavages = 0; and false discovery rate = 100. Only peptides that were identified in three of six (for each protease digestion regime) nondeuterated samples and with a minimum intensity of 25,000 counts, a maximum length of 30 amino acids, a minimum number of three products with at least 0.1 product per amino acid, a maximum mass error of 25 ppm, and a retention time tolerance of 0.5 min were considered for further analysis. Deuterium incorporation into peptides was quantified with the DynamX 3.0 software (Waters). Hereby, the datasets generated with pepsin digestion or after digestions with proteases type XIII and XVIII were pooled. All spectra were manually inspected and, if necessary, peptides omitted (e.g., in the case of low signal-to-noise ratio or the presence of overlapping peptides).

The observable maximal deuterium uptake of a peptide was calculated by the number of residues minus one (for the N-terminal residue) minus the number of proline residues contained in the peptide. For the calculation of HDX in percent, the absolute HDX was divided by the theoretical maximal deuterium uptake multiplied by 100. To render the residue specific HDX differences from overlapping peptides for any given residue of HilD, the shortest peptide covering this residue was used. Where multiple peptides were of the shortest length, the peptide with the residue closest to the peptide’s C terminus was used.

### Microscale thermophoresis

MST measurements for the binding of HilD to HilE were performed as previously described (*39*). In summary, enhanced yellow fluorescent protein (EYFP)-HilD (100 nM) was incubated with 100 μM either oleic acid or C26 [with a final concentration of 1% (v/v) DMSO] for 10 min at r.t. (22° to 25°C) and subsequently mixed 1:1 with varying concentrations of HilE. Samples were incubated together for 10 min at r.t., centrifuged for 5 min, and loaded to standard capillaries (NanoTemper Technologies GmbH, no. MO-K022). MST runs were performed at 25°C on a NanoTemper Monolith NT.115, with an excitation power of 60% and medium MST power. Data were analyzed using the MO.Affinity Analysis v2.3 software, and affinity constants were calculated using the *K*_d_ model.

### Subcellular quantification of uptake

*S.* Typhimurium was grown in Mueller-Hinton-2 medium to an OD_600_ of 0.8 and incubated with the inhibitors (100 ng/ml, ~250 nM) for 10 min. Cells were then subjected to a fractionation protocol as previously described (*43*). The obtained fractions were protein depleted via precipitation using a mixture of H_2_O/ACN/MeOH (40/30/30) and centrifugation at 3000 rpm in a cold environment (4°C). The supernatant was evaporated in a CentriVap (Labconco, Kansas, MO, USA) device overnight at 30°C before resuspending in 50 μl of appropriate LC/MS/MS buffer, containing caffeine (10 ng/ml) as internal standard. Results were generated on a triple quadrupole mass spectrometer (AB Sciex 6500, Darmstadt, Germany) connected to an Agilent 1290 Infinity II UHPLC (Agilent Technologies, Santa Clara, CA, USA). Separation was done via reversed phase with an RP-18 column (Phenomenex Gemini, 3 μm NX-C18 110A, 50 mm by 2 mm) with a respective column guard (5 mm by 2 mm, Phenomenex, Torrance, CA, USA) at a flow rate of 700 μl/min and an elution gradient from 5 to 95% B within 4 min (A: H_2_O + 0.1% HCOOH; B: ACN + 0.1% HCOOH). Source parameters of the mass spectrometer and mass transitions are given in table S7. Calibration curves were recorded with the compounds in the respective matrices. Data were quantified with Multiquant 3.03 (AB Sciex, Darmstadt, Germany).

### Statistics

Statistical analyses were conducted using GraphPad Prism 10.1.1. Data are presented as means ± SD. Comparisons with *P* > 0.05 were not considered significant.

## Supplementary Materials

### The PDF file includes:

Figs. S1 to S15 Tables S1 to S10 Legend for data S1 Data S2 References
