# Supplementary material for: Discovery of synthetic small molecules targeting the central regulator of Salmonella pathogenicity
Source: Sci Adv. 2025 Apr 11;11(15):eadr5235. doi: 10.1126/sciadv.adr5235 (PMC11988454; doi:10.1126/sciadv.adr5235)
Supplement: Supplementary file 1 — Figs. S1 to S15 Tables S1 to S10 Legend for data S1 Data S2 References [file sciadv.adr5235_sm.pdf]

Science Advances
AAAS

Supplementary Materials for

Discovery of synthetic small molecules targeting the central regulator of *Salmonella* pathogenicity

Abdelhakim Boudrioua *et al.*

Corresponding author: Samuel Wagner, samuel.wagner@med.uni-tuebingen.de

Sci. Adv. **11**, eadr5235 (2025)
DOI: 10.1126/sciadv.adr5235

The PDF file includes:

Figs. S1 to S15
Tables S1 to S10
Legend for data S1
Data S2
References

Other Supplementary Material for this manuscript includes the following:

Data S1

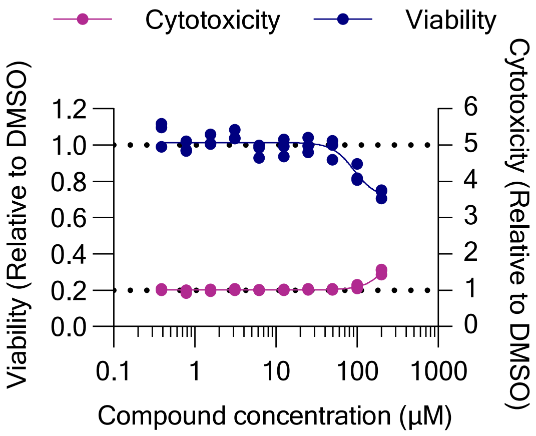

Fig. S1.

in vitro toxicity in HeLa cells exposed to C26 for 12 h using the ApoTox-Glo™ assay. Fluorescence was measured as a readout for viability at $400_{Ex}/505_{Em}$ and cytotoxicity at $485_{Ex}/520_{Em}$. N = 3 biological replicates.

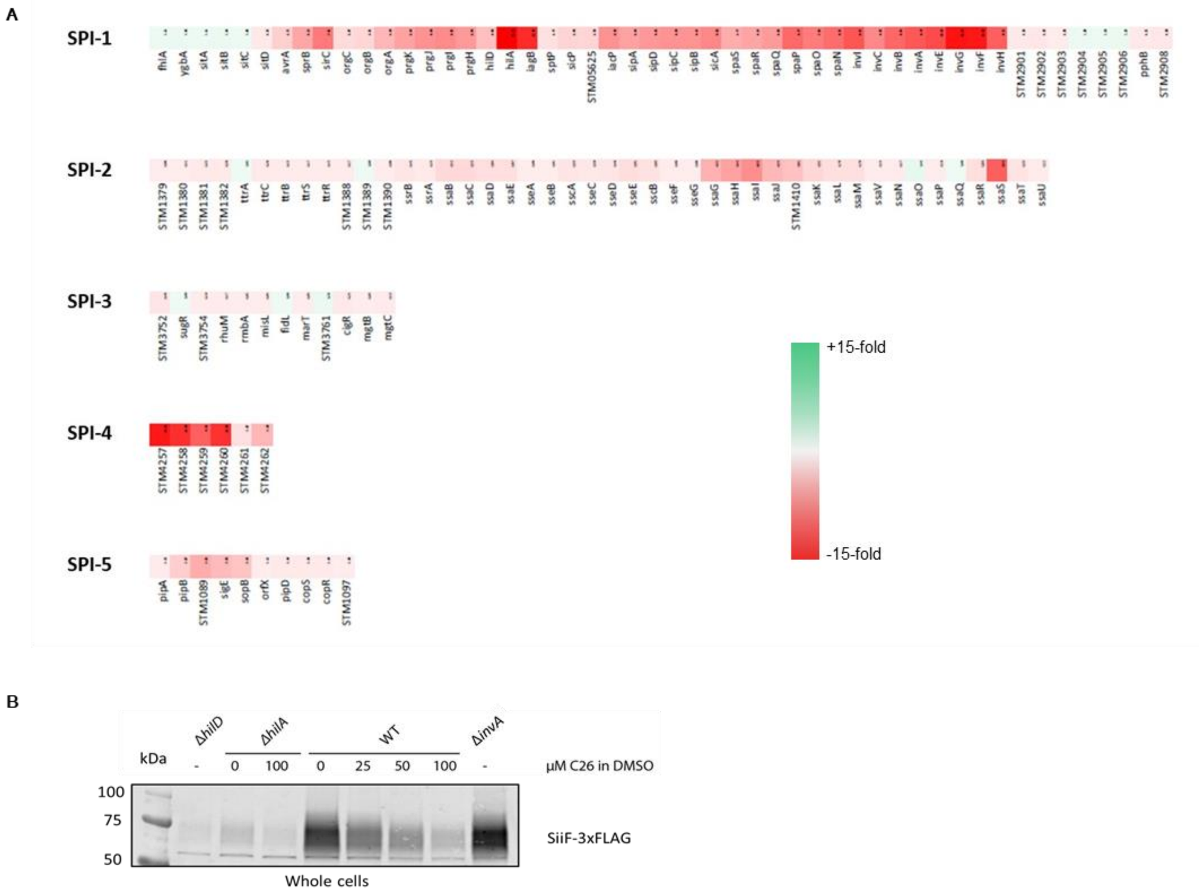

Fig. S2.

Effect of C26 on the expression of invasion genes. **A)** Heat map of mRNA fold-changes from genes encoded in SPI-1-5. **B)** Monitoring the expression of FLAG-tagged T1SS structure protein SiiF by Western blotting using mouse anti-FLAG (1:10000) antibody in the indicated *Salmonella* strains.

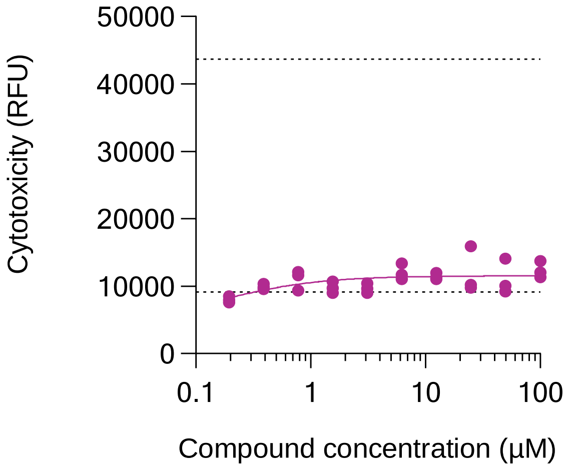

Fig. S3.

in vitro toxicity in macrophages RAW 264.7 exposed to C26 for 6 h using the CellTox™ Green Cytotoxicity Assay. Fluorescence was measured as a readout for cytotoxicity at $485_{Ex}/520_{Em}$. N = 3 biological replicates.

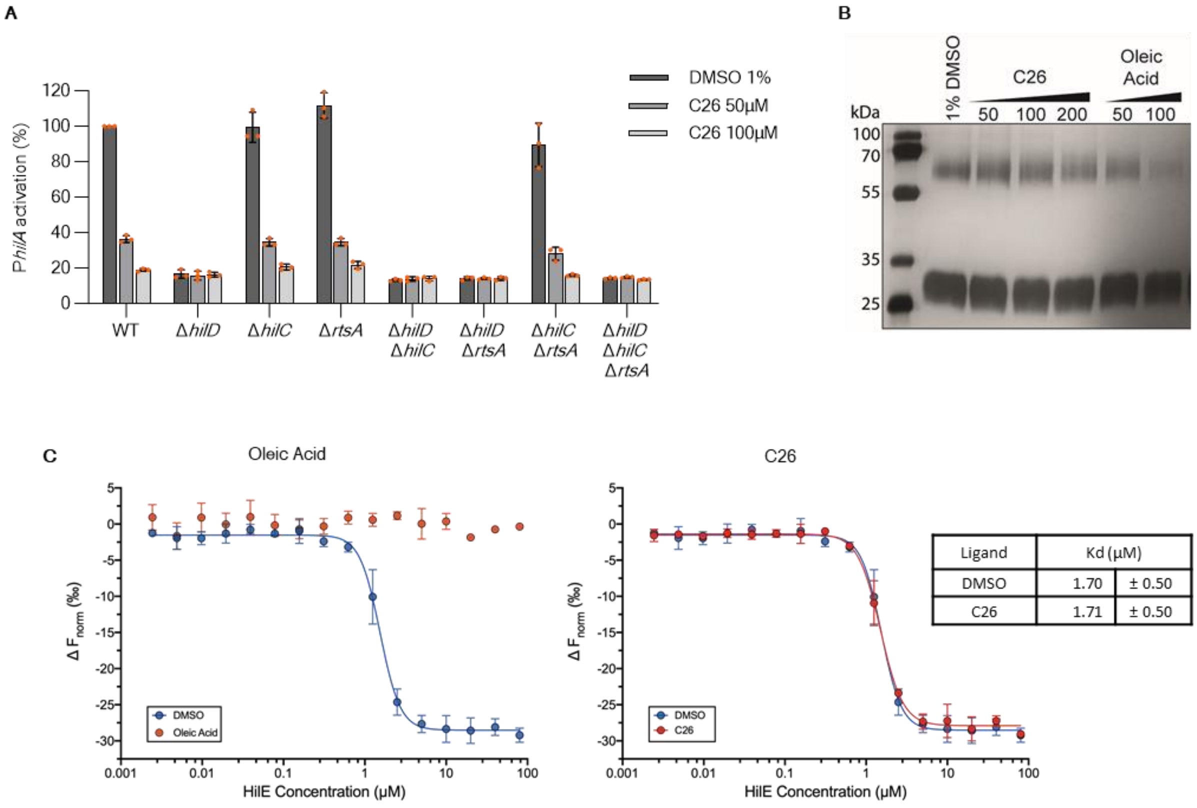

Fig. S4.

Effect of C26 on HilD dimerization and activity. **A)** Whole cell P_{hilA} activation assay to assess the effect of C26 on HilD, HilC, and RtsA transcriptional activity levels (N = 3 biological replicates). **B)** HilD dimerization investigated by BS^3 cross-linking of HilD monomers (10 µM) in the presence of C26 (50, 100, and 200 µM) or oleic acid (50 and 100 µM). **C)** MST assay to investigate the effect of C26 and oleic acid on the *in vitro* formation of the HilD-HilE heterodimer. EYFP-HilD was incubated with either 1% DMSO (blue), 100 µM oleic acid (orange) or 100 µM C26 (red) and increasing concentrations of HilE. Data show changes in thermophoresis at an MST on-time of 1.5 s and represents the mean ± SD of four replicates.

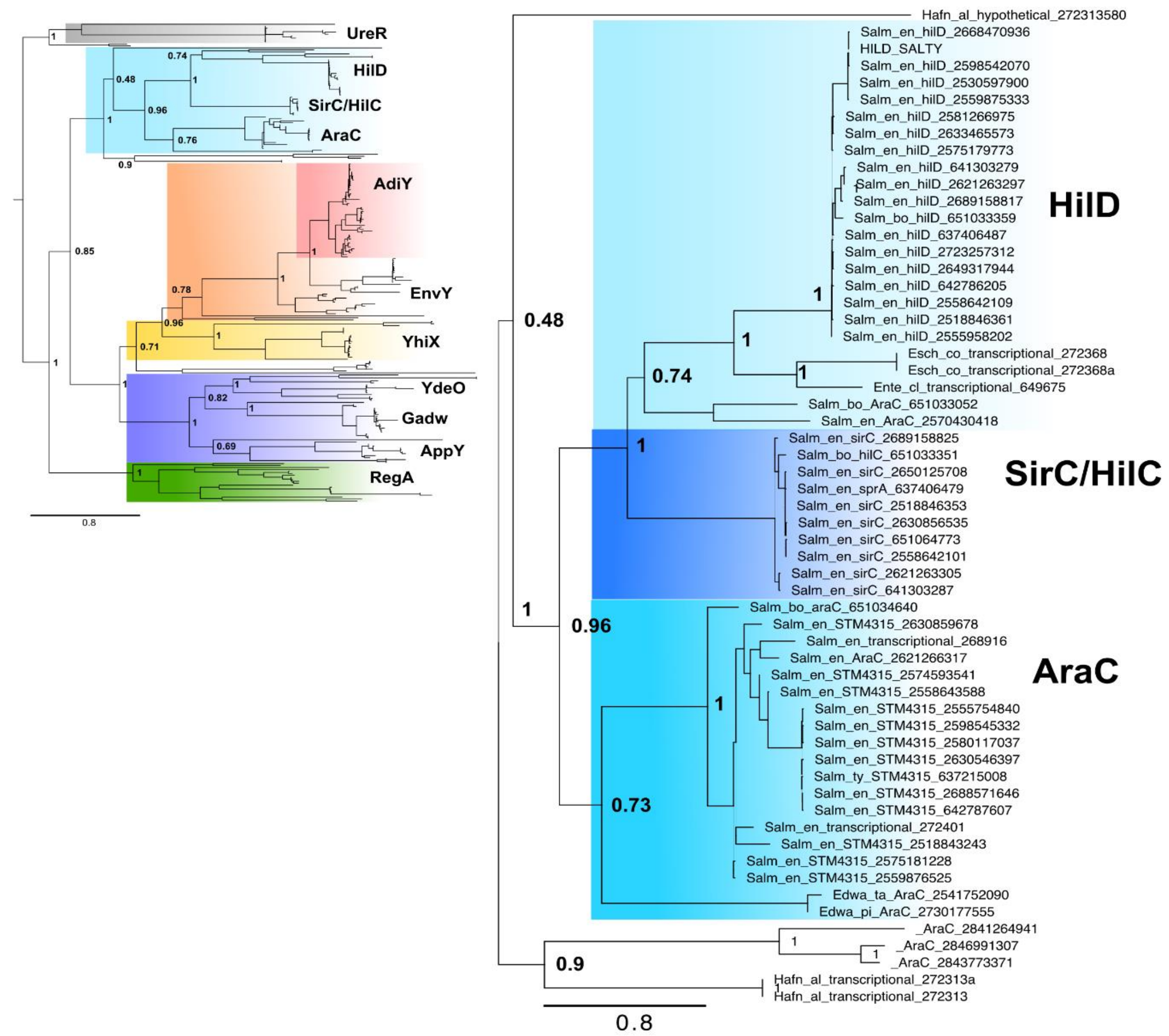

Fig. S5.

Dendrogram showing homologs of HilD. The tree was generated with FigTree (v1.4.4). Left: overview of all members of AraC/XylS family protein grouped. Right: subclade containing HilD, HilC and AraC from *Salmonella* spp. highlighted in blue. Only clades with branch statistical support of posterior probability values (calculated using aBayes) with values >0.7 or relevant for discussion are displayed.

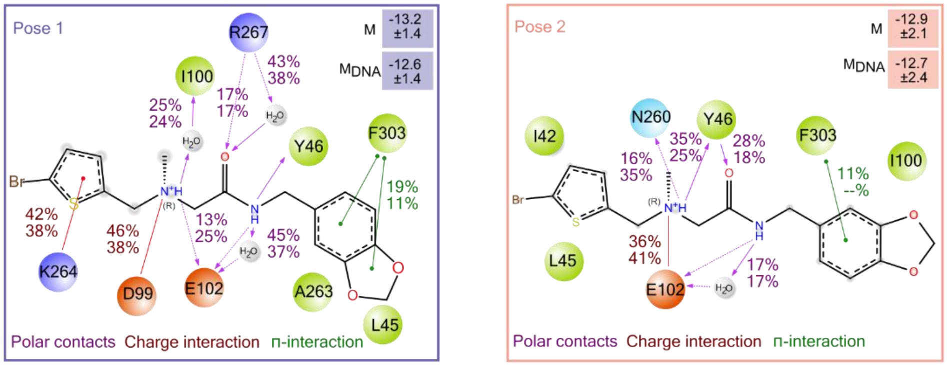

Fig. S6.

2D schematic representation of C26 in pose 1 and pose 2 potential binding modes summarizing their interaction frequency along the analyzed trajectory of MD simulations (~10 µs per ligand). Interaction frequency (%) in the upper labels are derived from monomers + DNA while the below numbers derive from simple monomeric simulations. Polar interactions are depicted in purple, charged interactions in red and π-mediated interactions as green lines. Quantification of the predicted binding energy for each ligand along the simulated trajectory, using MM/GBSA (see extended methods for calculation). The median of the calculated energies is displayed as colored boxes, together with its standard deviation, and free energy binding calculation (kcal/mol normalized by the Heavy Atoms Count, HAC).

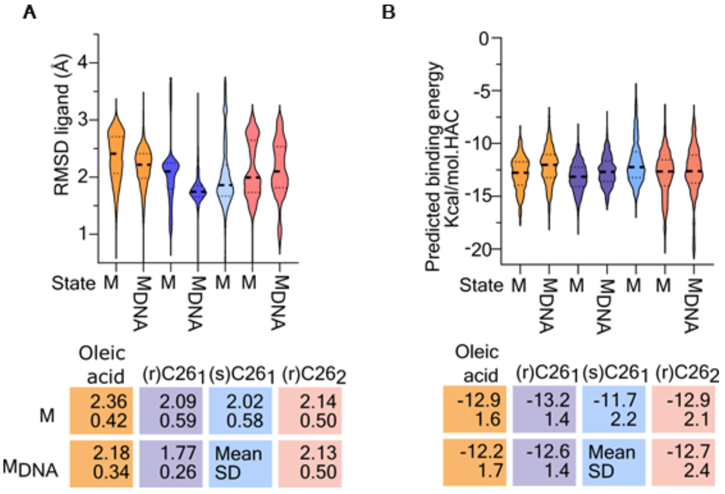

Fig. S7.

Violin plots depicting the **A)** Root mean square deviation of the ligand's heavy atoms along the trajectory time (~10 µs per ligand). **B)** Quantification of the predicted binding energy for each ligand along the simulated trajectory, using MM/GBSA (see extended methods for calculation), in the free energy binding calculations (kcal/mol normalized by the Heavy Atoms Count, HAC).

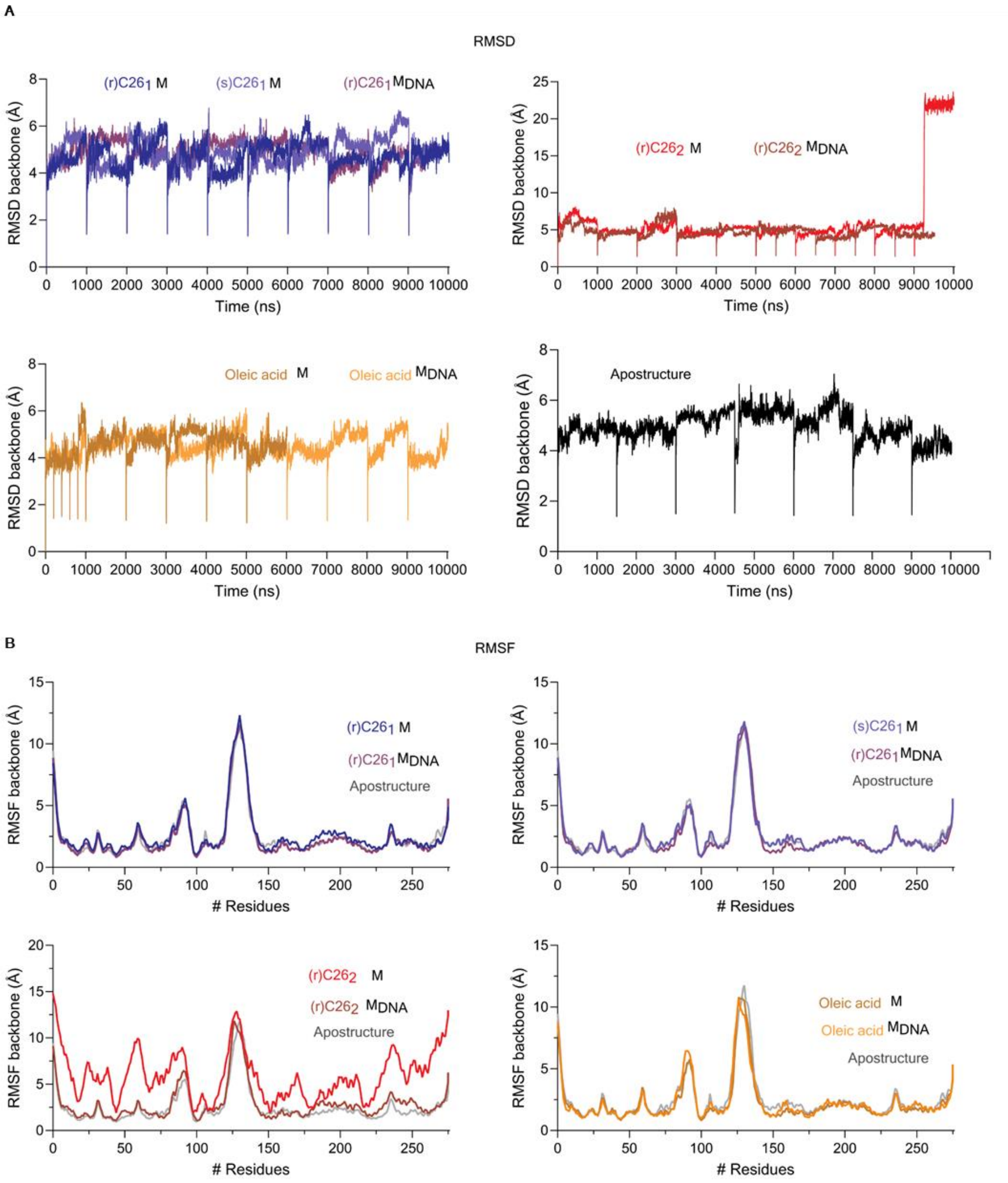

Fig. S8.

Protein’s backbone RMSD **(A)** and RMSF **(B)** were used to evaluate the equilibration of the system.

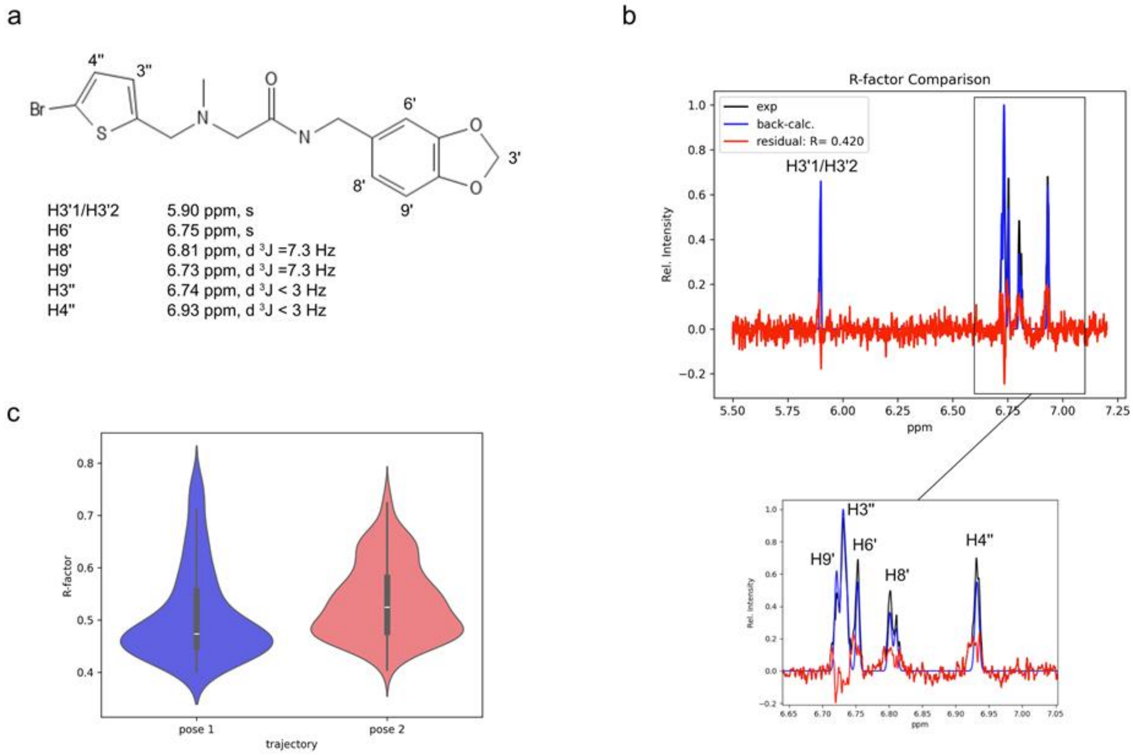

Fig. S9.

NMR saturation transfer difference (STD) experiments favor pose 1 over pose 2. **A)** NMR assignment of C26 protons considered in the STD analysis (s; singlet, d; doublet). **B)** Example of back-calculation of expected STD intensities using the CORCEMA algorithm. The program takes ligand affinity, protein and ligand concentrations and parameters of the NMR experiment into account to predict the relative intensities of STD peaks. In the current implementation, a simulated spectrum is also calculated based on ligand chemical shifts and couplings and an R-factor calculated on basis of RMSD to the experimental spectrum. Only the region shown was included in quantitative analysis. The experimental data is shown in black, while the back-calculated and residual are in blue and red, respectively. The inset shows the aromatic signals; note that H9’ and H3’’ are partially overlapped. The comparison shown is for the best frame from the two MD trajectories; frame 8 of the pose 1 trajectory (R-factor 0.420). **C)** Violin plots for the distribution of R-factors in MD trajectories starting from pose 1 and pose 2. The two distributions are significantly different (Mann-Whitney p-value < 1e-06), with the pose 1 trajectory enriched in frames with lower R-factors. All data shown is for a saturation time of 800 ms, representing a compromise between STD intensity and sensitivity to the ligand pose.

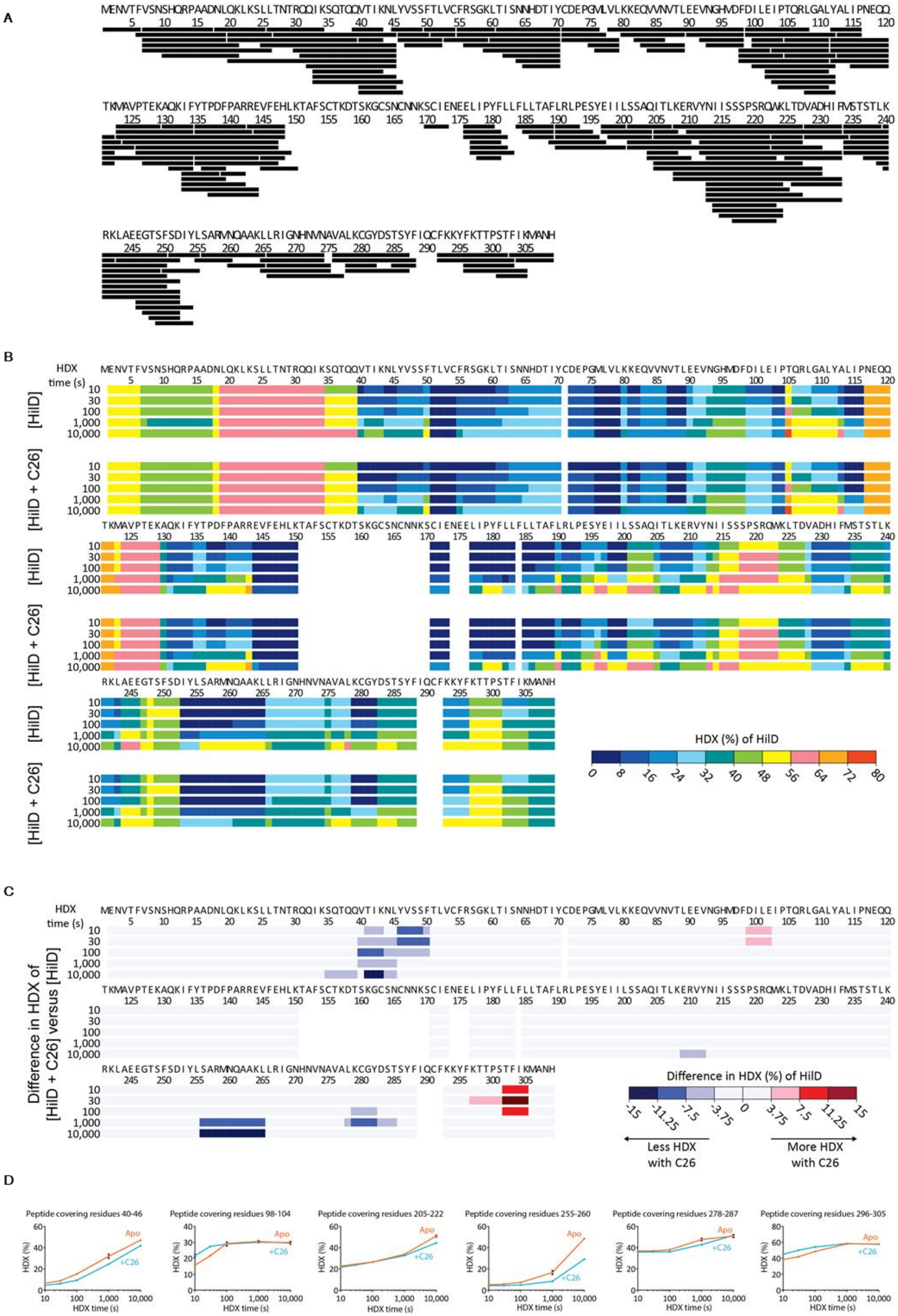

A
MENVTFVSNSHQRPAADNLQKLKSLLTNTRQQIKSQTQQVTIKNLYVSSFTLVCFRSGKLTISNNHDTIYCDEPGMLVLKKEQVVNVTLEEVNGHMDFDILEIPTQRLGALYALIPNEQQ
TKMAVPTEKAQKIFYTPDFPARREVFEHLKTAFSCTKDTSKGCSNCNNKSCIENEELIPYFLLFLLTAFLRLPESYEIILSSAQITLKERVYNIISSSPSRQWKLTDVADHIFMSTSTLK
RKLAEEGTSFSDIYLSARMNQAAKLLRIGNHNVNAVALKCGYDSTSYFIQCFKKYFKTTPSTFIKMANH
B
HDX time (s)
[HilD]
[HilD + C26]
10
30
100
1,000
10,000
HDX (%) of HilD
0 8 16 24 32 40 48 56 64 72 80
C
Difference in HDX of [HilD + C26] versus [HilD]
Difference in HDX (%) of HilD
-15 -11.25 -7.5 -3.75 0 3.75 7.5 11.25 15
Less HDX with C26
More HDX with C26
D
Peptide covering residues 40-46
Peptide covering residues 98-104
Peptide covering residues 205-222
Peptide covering residues 255-260
Peptide covering residues 278-287
Peptide covering residues 296-305
HDX (%)
HDX time (s)
Apo
+C26

Fig. S10.

HDX-MS of HilD. **A)** Each black bar denotes a peptide of HilD identified during HDX-MS experiments. **B)** Residue-specific HDX for HilD, either in isolation or in presence of 100 µM C26, was obtained from peptides by employing the shortest peptide covering any residue. No HDX could be obtained for amino acid sequences in the gaps, which indicate regions not covered by any peptides. **C)** C26-dependent changes in HDX of HilD, expressed as the difference in residue-specific HDX between HilD in isolation (HilD) and in the presence of 100 µM C26 (HilD + C26). **D)** HDX of selected representative HilD peptides. Data represent mean ± s.d. of N = 3 technical replicates (individual HDX reactions).

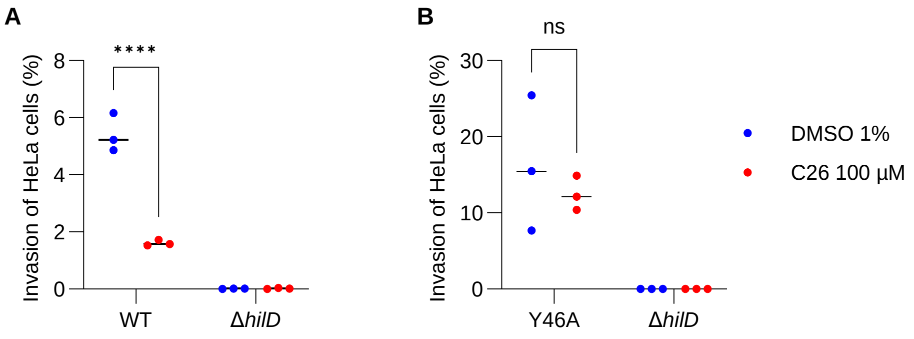

Fig. S11.

Invasion of HeLa cells by *S.* Typhimurium harboring $HilD_{WT}$ **(A)** or the C26-resistant $HilD_{Y46A}$ **(B)**. MOI: 20. Δ*hilD* mutant was used as a negative control. ns, not significant. **** $p < 0.0001$ (Bonferroni's multiple comparisons test). N = 3 biological replicates.

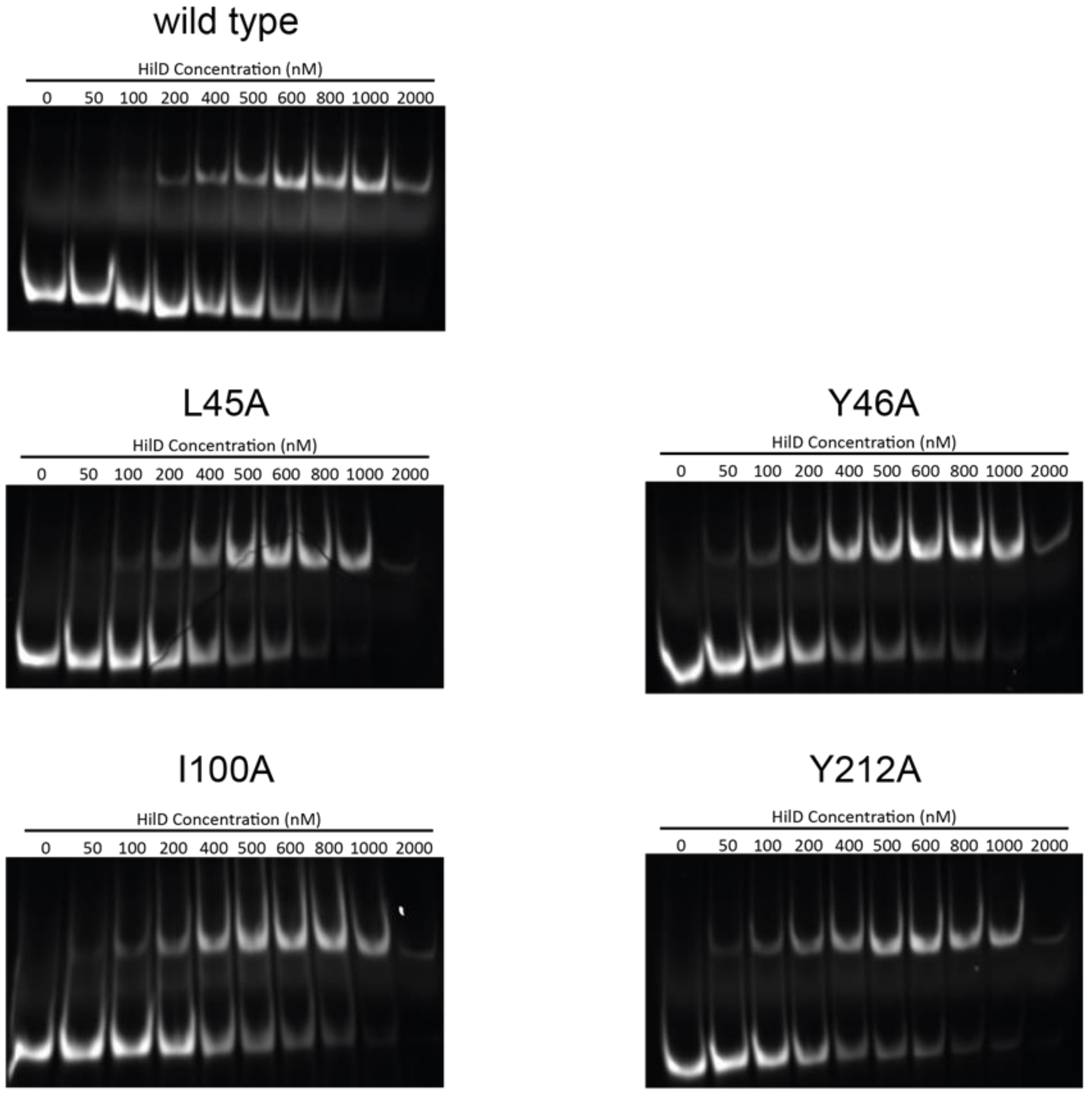

Fig. S12.

Electrophoretic mobility shift assay (EMSA) showing the binding of purified HilD mutants to the promoter of *hilA*.

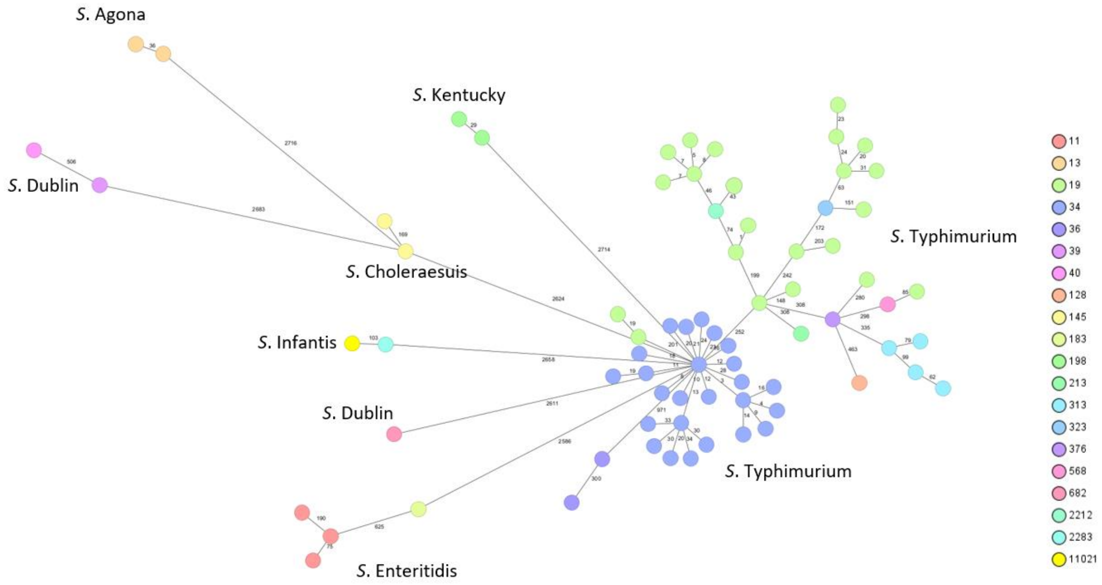

Fig. S13.

Minimum spanning tree of 75 selected *S. enterica* strains from Germany based on cgMLST (Ridom SeqSphere+, 3.002 alleles Enterobase cgMLST scheme). Color-coding based on sequence type.

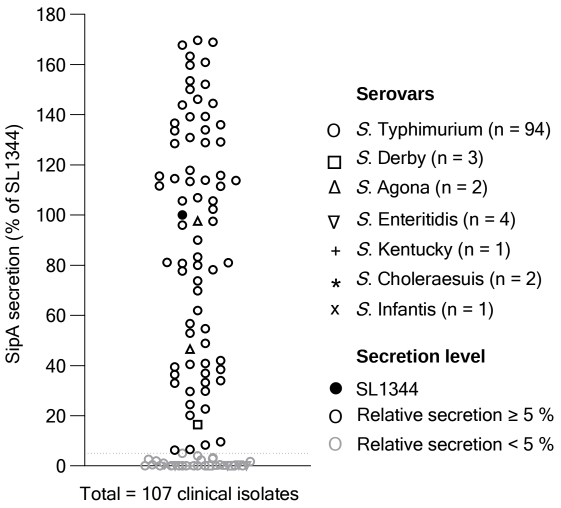

Fig. S14.

Secretion levels of SipA in clinical isolates of *S. enterica* relative to SL1344.

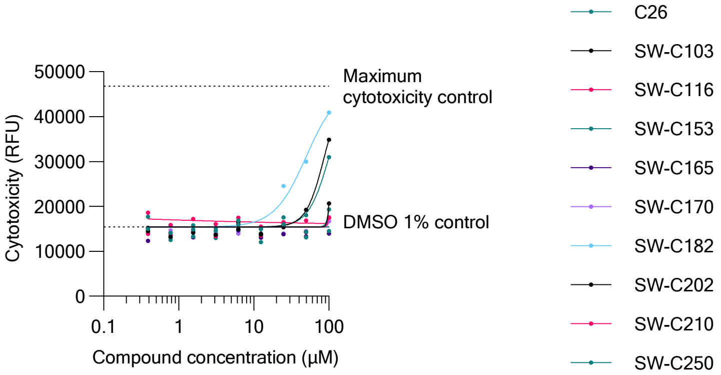

Fig. S15.

in vitro cytotoxicity of C26 and analogs. Cytotoxicity assessed with CellTox™ Green Cytotoxicity Assay with an exposure time of 18 h. N = 3 biological replicates. Data were plotted and TC_{50} values were generated with GraphPad Prism 10.1.1.

Table S1.

Chemical properties of compound C26. Values were generated with ChemAxon and DataWarrior. Molecular weight (MW), H-bond donor (HBD) and acceptor (HBA), isoelectric point (pI). a: Value interval of the majority of traded drugs. From https://openmolecules.org/properties/properties.html (accessed on October, 2024).

Property	MW (g/mol)	clogP	clogS	HBD	HBA	pI	Rotatable bonds	Druglikeness
Value for C26	397	2.69	-3.79	1	5	10.4	6	2.68
Recommended value	<500(*44*)	<5(*44*)	–6.5 – 0.5[a]	<5(*44*)	<10(*44*)		<11(*88*)	0 – 5[a]
Compliance with Lipinski's rules	yes	yes		yes	yes			

Table S2.

Recorded death events of mice during the first 72 hours after treatment. PO, *per os*. Vehicle: 5% Tween 20, lecithin (from soy bean: 40 mg in 800 μL distilled water), 45 % PBS.

Compound	Route	Dose (mg/kg)	Toxicity (death / test)				
			1 h	2 h	24 h	48 h	72 h
Vehicle	PO	5 ml/kg	0/3	0/3	0/3	0/3	0/3
C26	PO	3	0/3	0/3	0/3	0/3	0/3
		10	0/3	0/3	0/3	0/3	0/3
		30	0/3	0/3	0/3	0/3	0/3

Table S3.

Recorded body weights of mice before treatment and at 72 hours after treatment. PO, *per os*. Vehicle: 5% Tween 20, lecithin (from soy bean: 40 mg in 800 μL distilled water), 45% PBS.

Compound	Route	Dose (mg/kg)	No.	Body weight (g)	
				Pre-dose	72 h
Vehicle	PO	5 ml/kg	1	28	31
			2	26	30
			3	27	31
C26	PO	3	1	26	29
			2	28	29
			3	27	29
		10	1	25	27
			2	25	28
			3	25	27
		30	1	25	29
			2	26	29
			3	28	31

Table S4.

Molecular mass determination by SEC-MALS analysis of HilD in the presence of DMSO (1%) or C26 (100 µM). Values correspond to the curves shown in Fig. 3H.

Construct	Molecular Mass (kDa)		
	Theoretical (Dimer)	Measured (± SD)	
HilD	70.4	70.4	± 2.0
HilD + DMSO	70.4	71.4	± 2.8
HilD + C26	70.4	69.8	± 1.8

Table S5.

List of the 50 most frequent substitutions found among 2,351 sequences of *S. enterica*.

Substitution	#	Frequency (10^{-3})
N86S	608	258,6134
R30Q	259	110,1659
A124S	257	109,3152
S220G	238	101,2335
V40I	227	96,55466
A275S	219	93,15185
A110D	217	92,30115
D72E	182	77,41387
K169Q	142	60,39983
A130V	126	53,59422
V228I	122	51,89281
T226P	115	48,91536
V125I	108	45,9379
T127V	108	45,9379
S164N	103	43,81114
N165S	102	43,38579
G162S	100	42,53509
V125K	97	41,25904
P137S	96	40,83369
K157M	92	39,13228
D230E	90	38,28158
M123R	80	34,02807
K169R	80	34,02807
T121I	79	33,60272
I215L	77	32,75202
F134I	71	30,19991
R221K	68	28,92386
E209K	58	24,67035
E73A	55	23,3943
M306I	54	22,96895
T37A	49	20,84219
K305F	48	20,41684
A307K	47	19,99149
N308M	45	19,14079
S249N	44	18,71544
H309A	43	18,29009

K305R	35	14,88728
A124V	34	14,46193
K279T	33	14,03658
H231N	30	12,76053
T127A	29	12,33518
F134L	25	10,63377
M96L	24	10,20842
S237P	22	9,35772
T41S	21	8,932369
K43R	21	8,932369
K150R	19	8,081667
E128D	18	7,656316
S216Y	18	7,656316
I62V	16	6,805615

Table S6.

Clinical isolates of *S. enterica*. UKT, University hospital of Tübingen. RKI, Robert Koch Institute. Antibiotic susceptibilities were investigated by broth microdilution according to EUCAST criteria (The European Committee on Antimicrobial Susceptibility Testing. Breakpoint tables for interpretation of MICs and zone diameters. Version 10.0, 2020. http://www.eucast.org) AMP, Ampicillin. MEZ, Mezlocillin. OTE, Oxytetracycline. SMZ, Sulfamethoxazole. STR, Streptomycin. CMP, Chloramphenicol. CTM, Cefotiam. CTX, Cefotaxime. MSU, Mezlocillin/Sulbactam. NAL, Nalidixic acid. SXT, Trimethoprim / Sulfamethoxazole. TCY, Tetracycline. AZM, Azithromycin. CAZ, Ceftazidime. CIP, Ciprofloxacin. FOX, Cefoxitin. GEN, Gentamicin. Table cells are left blank when the corresponding characteristic is unknown. *Identified using NCBI AMRFinderPlus(*89*).

Name	Source	Resistance phenotype	Resistance genes*	ST	Provider
***S*. Typhimurium**					
67708850	Blood				UKT
67529692	Stool				UKT
67962944	Stool				UKT
67775928	Stool				UKT
67774011	Stool				UKT
67738336	Stool				UKT
67713042	Urine				UKT
67708976	Stool				UKT
67681882	Stool				UKT
67625108	Stool				UKT
67564932	Stool				UKT
67547889	Stool				UKT
67463497	Stool				UKT
67459438	Stool				UKT
67453336	Urine				UKT
67447090	Stool				UKT
67444809	Stool				UKT
67411073	Stool				UKT
67189086	Stool				UKT
67187434	Stool				UKT
67178294	Stool				UKT
67065118	Stool				UKT
67064696	Stool				UKT
63957635	Blood				UKT
63852331	Blood				UKT
63804211	Blood				UKT
SL3	Stool				UKT
SL17	Stool				UKT
SL18	Stool				UKT

SL60	Stool				UKT
SL131	Stool				UKT
SL120	Stool				UKT
SL256	Stool				UKT
SL175	Stool				UKT
SL145	Stool				UKT
06-01900	Stool	AMP, MEZ, OTE, SMZ, STR	*bla*$_{TEM-1}$, *sul2*, *tet*(B)	34	RKI
18-02653		AMP, CMP, CTM, CTX, MEZ, MSU, NAL, OTE, STR	*bla*$_{CARB-2}$, *bla*$_{CTX-M-1}$, *floR*, *tet*(G)	19	RKI
19-00422	Stool	AMP, CTM, CTX, MEZ, MSU, OTE	*bla*$_{TEM-1}$, *bla*$_{CTX-M-1}$, *qnrS1*, *sul2*, *tet*(A) / *tet*(M)	19	RKI
19-02162	Blood	AMP, CAZ, FOX, CTM, CTX, MEZ, MSU, OTE, STR	*bla*$_{TEM-1}$, *bla*$_{CMY-2}$, *qnrB19*, *sul2*, *tet*(B)	34	RKI
19-04442	Stool	AMP, CTM, CTX, MEZ, MSU, STR, SXT	*bla*$_{CTX-M-1}$, *sul2*, *dfrA17*	34	RKI
19-05051	Stool	AMP, CTM, CTX, MEZ, OTE, STR, SXT	*bla*$_{CTX-M-1}$, *sul1*, *tet*(A), *dfrA1*	19	RKI
20-00098	Stool	AMP, CMP, MEZ, NAL, OTE, SXT	*bla*$_{TEM-1}$, *qnrB19*, *cmlA1*, *sul3*, *tet*(B), *dfrA12*	34	RKI
20-00760		AMP, CTM, CTX, MEZ, MSU, STR, SXT	*bla*$_{TEM-1}$, *bla*$_{CTX-M-1}$, *qnrS1*, *sul1* / *sul2*, *dfrA12*	34	RKI
20-01187	Stool	AMP, MEZ, OTE, STR	*bla*$_{TEM-1}$, *sul2*, *tet*(B)	34	RKI
20-01384	Stool	AMP, CMP, MEZ, MSU, STR	*bla*$_{CARB-2}$, *floR*, *tet*(G)	19	RKI
20-02017	Stool	AMP, MEZ, OTE, STR	*bla*$_{TEM-1}$, *sul2*, *tet*(B)	34	RKI

20-02167	Stool	AMP, MEZ, MSU, OTE, STR, SXT	*bla*$_{TEM-1}$, *sul1* / *sul2*, *tet*(A), *dfrA1*	34	RKI
20-02297	Stool	Sensitive	None	313	RKI
20-02498	Stool	AMP, MEZ, STR	*bla*$_{TEM-1}$, *sul2*, *tet*(B)	34	RKI
20-02749	Stool	AMP, CMP, MEZ, MSU, OTE, STR	*bla*$_{TEM-1}$, *floR*, *sul2*, *tet*(B)	34	RKI
20-03136		Sensitive	None	323	RKI
20-03498		AMP, CMP, MEZ, OTE, SXT	*bla*$_{TEM-1}$, *qnrS1*, *cmlA1* / *floR*, *sul2* / *sul3*, *tet*(A) / *tet*(M), *dfrA12*	19	RKI
20-03595		AMP, MEZ, MSU	*bla*$_{TEM-1}$	19	RKI
20-06170		AMP, TCY	*bla*$_{TEM-1}$, *sul2*, *tet*(B)	34	RKI
20-06645		Sensitive	None	19	RKI
21-00373	Stool	Sensitive	None	19	RKI
21-00797	Stool	AMP, AZM, CAZ, CTX, SXT, TCY, TMP	*bla*$_{TEM-1}$, *bla*$_{CTX-M-1}$, *tet*(B), *dfrA17*	34	RKI
21-01183	Stool	AMP, CIP, NAL, TCY	*bla*$_{TEM-1}$, *qnrB19*, *sul2*, *tet*(B)	34	RKI
21-01586	Stool	AMP, CIP, CMP, NAL, TCY	*bla*$_{CARB-2}$, *gyrA*_D87N, *floR*, *tet*(G)	19	RKI
21-02490	Stool	AMP, TCY	*bla*$_{TEM-1}$, *sul2*, *tet*(B)	34	RKI
21-02635	Stool	AMP	*bla*$_{TEM-1}$	2212	RKI
21-03310	Stool	AMP, CAZ, CTX	*bla*$_{CTX-M-1}$	34	RKI
21-03504	Stool	AMP, CIP, NAL, SXT, TCY, TMP	*bla*$_{TEM-1}$, *qnrB19*, *sul2*, *tet*(A), *dfrA5*	19	RKI
21-03926	Stool	AMP, TCY	*bla*$_{TEM-1}$, *sul2*, *tet*(B)	34	RKI
21-03980	Stool	Sensitive	None	19	RKI
21-06117	Stool	CIP, NAL	*qnrB19*, *sul1*	19	RKI
21-06637	Stool	Sensitive	None	213	RKI
22-00115	Stool	AMP, TCY	*bla*$_{TEM-1}$, *sul2*, *tet*(B)	34	RKI
22-00733	Stool	Sensitive	None	128	RKI

22-00857	Stool	Sensitive	None	568	RKI
22-01508	Stool	AMP, TCY	*bla*$_{TEM-1}$, *sul2*, *tet*(B)	34	RKI
22-02255	Stool	Sensitive	None	313	RKI
22-02256	Stool	AMP	*bla*$_{TEM-1}$	19	RKI
22-02294	Stool	Sensitive	None	376	RKI
22-03517	Stool	AMP, CAZ, CIP, CTX, FOX, TCY	*bla*$_{CMY-2}$, *qnrS1*, *tet*(A)	36	RKI
22-03880	Stool	AMP	*bla*$_{TEM-1}$	19	RKI
22-03956		AMP, CTX	*bla*$_{TEM-1}$, *bla*$_{CTX-M-1}$	19	RKI
22-04189	Stool	AMP, CAZ, CIP, CTX	*bla*$_{TEM-1}$, *bla*$_{CTX-M-1}$, *qnrS1*	19	RKI
22-04406	Stool	Sensitive	None	19	RKI
22-05027	Stool	Sensitive	None	36	RKI
22-05885	Urine	AMP, CHL, TCY	*bla*$_{CARB-2}$, *floR*, *tet*(G)	19	RKI
22-06301	Stool	SXT, TMP	*sul1* / *sul2*, *dfrA1*	34	RKI
22-06509	Stool	Sensitive	None	19	RKI
22-07856		AMP, CIP, CTX, SXT, TCY, TMP	*bla*$_{CTX-M-1}$, *qnrS1*, *sul1*, *tet*(A), *dfrA12*	34	RKI
22-07859	Blood	AMP	*bla*$_{TEM-1}$	19	RKI
22-07954	Stool	AMP, CIP, NAL, TCY	*bla*$_{TEM-1}$, *qnrB19*, *sul2*, *tet*(B)	34	RKI
23-00671		AMP, TCY	*bla*$_{TEM-1}$, *sul2*, *tet*(B)	34	RKI
23-00837	Blood	Sensitive	None	19	RKI
23-00954	Stool	Sensitive	None	19	RKI
23-01121	Blood	Sensitive	None	313	RKI
23-01218	Blood	AMP, SXT, TMP	*bla*$_{TEM-1}$, *sul1* / *sul2*, *dfrA1*	313	RKI
23-04527	Blood	AMP	*bla*$_{TEM-1}$	19	RKI
**Other *S. enterica* serovars**					
18-00670, Serovar Choleraesuis	blood	STR	None	145	RKI
18-04107, Serovar Enteritidis		Sensitive	None	11	RKI
18-04810, Serovar Agona	stool	Sensitive	None	13	RKI

19-01006, Serovar Choleraesuis		Sensitive	None	145	RKI
19-01481, Serovar Enteritidis	stool	Sensitive	None	11	RKI
19-02948, Serovar Kentucky	stool	AMP, CIP, GEN, MEZ, MSU, NAL, OTE, STR	*bla*$_{TEM-1}$, *gyrA*_D87Y / *gyrA*_S83F / *parC*_S80I, *sul1*, *tet*(A)	198	RKI
19-03178, Serovar Agona	stool	Sensitive	None	13	RKI
20-05235, Serovar Derby	stool	Sensitive	None	39	RKI
20-06129, Serovar Enteritidis	stool	Sensitive	None	11	RKI
21-01720, Serovar Infantis	stool	AMP, CIP, CMP, NAL, SXT, TCY, TMP	*bla*$_{TEM-1}$, *gyrA*_S83Y, *cmlA1*, *sul1* / *sul3*, *tet*(A), *dfrA8*	2283	RKI
21-02525, Serovar Derby	stool	Sensitive	None	682	RKI
21-02979, Serovar Enteritidis	stool	Sensitive	None	183	RKI
21-06414, Serovar Derby	stool	Sensitive	None	40	RKI

Table S7.
Mass spectrometric parameters used for the quantification of C26 and SW-C182.

	Q1 (mass) [g/mol]	Q3 (mass) [g/mol]	Declustering potential [V]	Collision energy [V]	Collision cell exit potential [V]
**Caffeine (IS)**					
quantifier	195.116	138.1	81	27	10
qualifier	195.116	110.1	81	31	6
**C26**					
quantifier	397.52	174.7	11	25	10
qualifier	397.52	134.9	11	21	10
**SW-C182**					
quantifier	432,96	175.0	1	23	12
qualifier	432,96	96.1	1	81	12

Table S8.

List of strains

Name	Genotype	Parental strain	Source
Escherichia coli			
pir116	F- *mcrA* Δ*(mrr-hsdRMS-mcrBC) ϕ80dlacZ*Δ*M15* Δ*lacX74 recA1 endA1 araD139* Δ*(ara, leu)7697 galU galK λ– rpsL nupG pir-116(DHFR)*		(*90*)
β2163	(F-) RP4-2-Tc::Mu ΔdapA::(erm-pir)		(*91*)
NEB5α	*fhuA2* Δ(*argF-lacZ)U169 phoA glnV44 Φ80* Δ*(lacZ)M15 gyrA96 recA1 relA1 endA1 thi-1 hsdR17*		NEB
C41(DE3)	F- *ompT hsdSB* (rB- mB-) *gal dcm* (DE3)		Sigma-Aldrich
***Salmonella* Typhimurium**			
SL1344	Wild type		(*53*)
NCTC 12023	Wild type		(*54*)
SB1751	Δ*invA*		(*66*)
MIB4841	Δ*hilD*. Made by allelic exchange. Suicide plasmid pMIB5779	SL1344	This study
MIB5363	Δ*hilA*. Made by allelic exchange. Suicide plasmid pMIB7636	SL1344	This study
MIB5371	Δ*rtsA*. Made by allelic exchange. Suicide plasmid pMIB7639	SL1344	This study
MIB5585	Δ*hilC*. Made by allelic exchange. Suicide plasmid pMIB7633	SL1344	This study
MIB5591	Δ*hilC* Δ*rtsA*. Made by allelic exchange. Suicide plasmid pMIB7639	MIB5585	This study
MIB5587	Δ*hilD* Δ*hilC*. Made by allelic exchange. Suicide plasmid pMIB7633	MIB4841	This study
MIB5373	Δ*hilD* Δ*rtsA*. Made by allelic exchange. Suicide plasmid pMIB7639	MIB4841	This study
MIB5593	Δ*hilD* Δ*hilC* Δ*rtsA*. Made by allelic exchange. Suicide plasmid pMIB7639	MIB5587	This study
MIB3231	*sipA*-NLuc-*myc*		(*37*)
MIB3233	Δ*invA sipA*-NLuc-*myc*		(*37*)
MIB5063	Δ*hilD sipA*-NLuc-*myc.* Made by allelic exchange. Suicide plasmid pMIB5779	MIB3231	This study
MIB3877	*sipA*-3xFLAG-HiBit		(*37*)
MIB3879	Δ*invA sipA*-3xFLAG-HiBit		(*37*)
MIB5849	*siiE*::K5411HiBiT. Made by allelic exchange. Suicide plasmid pMIB8021	SL1344	This study
MIB5853	Δ*siiF siiE*-HiBiT. Made by allelic exchange. Suicide plasmid pMIB8023	MIB5849	This study

MIB5850	Δ*hilD siiE*-HiBiT. Made by allelic exchange. Suicide plasmid pMIB5779	MIB5849	This study
MIB5731	*siiF*-3xFLAG *sipA*-NLuc-*myc*. Made by allelic exchange. Suicide plasmid pMIB7882	MIB3231	This study
MIB5733	Δ*hilD siiF*-3xFLAG *sipA*-NLuc-*myc*. Made by allelic exchange. Suicide plasmid pMIB7882	MIB4841	This study
MIB5735	Δ*invA siiF*-3xFLAG *sipA*-NLuc-*myc*. Made by allelic exchange. Suicide plasmid pMIB7882	SB1751	This study
MIB5378	Δ*hilA sipA*-NLuc-*myc*. Made by allelic exchange. Suicide plasmid pMIB7636	MIB3231	This study
MIB5737	Δ*hilA siiF*-3xFLAG *sipA*-NLuc-*myc*. Made by allelic exchange. Suicide plasmid pMIB7882	MIB5378	This study
MvP1890	Δ*ssaV*::FRT	NCTC 12023	(*54*)
MIB5600	P_{hilA}-sfGFP. Made by allelic exchange. Suicide plasmid pMIB7644	MIB5600	This study
MIB5633	Δ*hilD* P_{hilA}-sfGFP. Made by allelic exchange. Suicide plasmid pMIB5779	MIB5600	This study
MIB5635	Δ*hilC* P_{hilA}-sfGFP. Made by allelic exchange. Suicide plasmid pMIB7633	MIB5600	This study
MIB5637	Δ*rtsA* P_{hilA}-sfGFP. Made by allelic exchange. Suicide plasmid pMIB7639	MIB5600	This study
MIB5639	Δ*hilD* Δ*hilC* P_{hilA}-sfGFP. Made by allelic exchange. Suicide plasmid pMIB5779	MIB5635	This study
MIB5641	Δ*hilD* Δ*rtsA* P_{hilA}-sfGFP. Made by allelic exchange. Suicide plasmid pMIB5779	MIB5637	This study
MIB5643	Δ*hilC* Δ*rtsA* P_{hilA}-sfGFP. Made by allelic exchange. Suicide plasmid pMIB7633	MIB5637	This study
MIB5645	Δ*hilD* Δ*hilC* Δ*rtsA* P_{hilA}-sfGFP. Made by allelic exchange. Suicide plasmid pMIB5779	MIB5643	This study
MIB3224	ΔSPI-1. Made by allelic exchange. Suicide plasmid p890-ΔSPI1	SB300	This study

Table S9.

Plasmids. If not otherwise specified, inserts were amplified from genomic DNA of *S.* Typhimurium SL1344.

**Plasmid**	**Relevant Genotype/Characteristic**	**Source**
**Plasmids derived from pSB890 backbone**		
pSB890	Tet^{R}, R6Kγ *ori*. Suicide plasmid	(*92*)
pMIB5779	Created by Gibson cloning<br>Vector: gib_uni_890_f2 / gib_uni_890_r2<br>Insert1: g_p890_hilD_A_f / g_KO_hilD_A_r<br>Insert2: g_KO_hilD_D_f / g_p890_hilD_D_r	This study
pMIB8021	Created by Gibson cloning<br>Vector: gib_uni_890_f2 / gib_uni_890_r2<br>Insert 1: gib_890_HiBiT_a_f / gib_HiBit_Ig53_b_r<br>Insert 2: gib_HiBit_Ig53_c_f / gib_890_HiBiT_d_r<br>Gibson assembly of inserts 1 and 2:<br>gib_890_HiBiT_a_f / gib_890_HiBiT_d_r	This study
pMIB8023	Created by Gibson cloning<br>Vector: gib_uni_890_f2 / gib_uni_890_r2<br>Insert 1: gib_890_dsiiF_a_f / gib_dsiiF_b_r on gDNA of MIB5849<br>Insert 2: gib_dsiiF_c_f / gib_890_dsiiF_d_r on gDNA of MIB5849	This study
pMIB7882	Created by Gibson cloning<br>Vector: gib_uni_890_f2 / gib_uni_890_r2<br>Insert 1: gib_890_ssiF_a_f / gib_FLAG_siiF_b_r<br>Insert 2: gib_FLAG_siiF_c_f / gib_890_siiF_d_r	This study
pMIB7636	Created by Gibson cloning<br>Vector: gib_uni_890_f2 / gib_uni_890_r2<br>Insert 1: gib_890_hilA_a_f / gib_890_hilA_b_r<br>Insert 2: gib_890_hilA_c_f / gib_890_hilA_d_r	This study
pMIB7639	Created by Gibson cloning<br>Vector: gib_uni_890_f2 / gib_uni_890_r2<br>Insert 1: gib_890_rtsAB_a_f / gib_890_rtsA_b_r<br>Insert 2: gib_890_rtsA_c_f / gib_890_rtsA_d_r	This study
pMIB7633	Created by Gibson cloning<br>Vector: gib_uni_890_f2 / gib_uni_890_r2<br>Insert 1: gib_890_hilC_a_f / gib_890_hilC_b_r<br>Insert 2: gib_890_hilC_c_f / gib_890_hilC_d_r	This study
pMIB7882	Created by Gibson cloning<br>Vector: gib_uni_890_f2 / gib_uni_890_r2<br>Insert 1: gib_890_siiF_a_f / gib_FLAG_siiF_b_r<br>Insert 2: gib_FLAG_siiF_c_f / gib_890_siiF_d_r	This study
pMIB7640	Created by Gibson cloning<br>Vector: gib_uni_890_f2 / gib_uni_890_r2	This study

	Insert 1: gib_890_hilA_a_f / gib_890_hilA::sfGFP_b_r<br>Insert 2: gib_890_hilA::sfGFP_c_f / gib_890_hilA_d_r<br>Insert 3: gib_sfGFP_f / gib_sfGFP_r	
pMIB7644	Derived from pMIB7640. Deletion of the 6 nucleotides "TACACT" between the RBS and the start codon by QuickChange with primers QC_hilA_SD-B_f and QC_hilA_SD-B_r	This study
p890-ΔSPI1	Created by Gibson cloning<br>Vector: gib_uni_890_f2 / gib_uni_890_r2<br>Insert 1: gib890_dSPI1_a_f / dSPI1_b_r<br>Insert 2: dSPI1invH_bc136f / dSPI1_d_r	This study
**Plasmids derived from pT10 backbone made by Gibson assembly**		
pT10	P_{rha}, Kan^R, SC101 ori. Synonym pSB3398	(*66*)
pMIB5776	pT10-*hilD*.<br>Vector: gib_uni_pT12_f / gib_uni_pT12_r<br>Insert: gib_pT12_hilD_f / gib_pT12_hilD_r	This study
pMIB5071	pT10-*sscB*-*sseF*-3xFLAG	(*93*)
pMIB7435	pT10-*sscB*-*sseF*-HiBiT-3xFLAG.<br>Vector: gib_HiBit_FLAG2_f/gib_HiBit_SseFc_r on pMIB5071	This study
pMIB8984	pT10-P*sseA*-*sscB*-*sseF*-HiBiT-3xFLAG.<br>Vector: gib_nSD_sscB_Fwd/ gib_pT10_KO_rha_Rv on pMIB7435<br>Insert: gib_pT10-KO-rha_ProsseA_f/ gib_ProsseA_nSD-SscB_r on gDNA of NCTC 12023	This study
pMIB7649	pT10-*hilC*.<br>Vector: gib_uni_pT12_f / gib_uni_pT12_r<br>Insert: gib_pT12_hilC_f / gib_pT12_hilC_r	This study
pMIB7648	pT10-*rtsA*.<br>Vector: gib_uni_pT12_f / gib_uni_pT12_r<br>Insert: gib_pT12_rtsA_f / gib_pT12_rtsA_r	This study
pMIB8048	Δ*rhaRS*-P_{rha}::P_{hilD}-*hilD*-P_{hilA}-sfGFP<br>Vector: gib_uni_pT12_f / gib_uni_pT10_rep_r<br>Insert: gib_PhilD_pT10_f / gib_sfGFP_pT10_rrnB_r on gDNA of MIB5600	This study
**Plasmids derived from pMIB8048 made by QuikChange**		
pMIB8306	P_{hilD}-P_{hilA}-sfGFP.<br>gib_PhilA_rep_f / gib_rep_PhilA_r	This study
pMIB8049	P_{hilD}-*hilD* Q31A-P_{hilA}-sfGFP	This study
pMIB8056	P_{hilD}-*hilD* I42A-P_{hilA}-sfGFP	This study
pMIB8057	P_{hilD}-*hilD* L45A-P_{hilA}-sfGFP	This study
pMIB8058	P_{hilD}-*hilD* Y46A-P_{hilA}-sfGFP	This study
pMIB8059	P_{hilD}-*hilD* S48A-P_{hilA}-sfGFP	This study

pMIB8050	P_{hilD}-*hilD* T51A-P_{hilA}-sfGFP	This study
pMIB8060	P_{hilD}-*hilD* V53A-P_{hilA}-sfGFP	This study
pMIB8501	P_{hilD}-*hilD* L60A-P_{hilA}-sfGFP	This study
pMIB8508	P_{hilD}-*hilD* L79A-P_{hilA}-sfGFP	This study
pMIB8513	P_{hilD}-*hilD* V85A-P_{hilA}-sfGFP	This study
pMIB8502	P_{hilD}-*hilD* V87A-P_{hilA}-sfGFP	This study
pMIB8509	P_{hilD}-*hilD* L89A-P_{hilA}-sfGFP	This study
pMIB8503	P_{hilD}-*hilD* F98A-P_{hilA}-sfGFP	This study
pMIB8504	P_{hilD}-*hilD* I100A-P_{hilA}-sfGFP	This study
pMIB8510	P_{hilD}-*hilD* L101A-P_{hilA}-sfGFP	This study
pMIB8301	P_{hilD}-*hilD* E102A-P_{hilA}-sfGFP	This study
pMIB8505	P_{hilD}-*hilD* Y212A-P_{hilA}-sfGFP	This study
pMIB8515	P_{hilD}-*hilD* I214A-P_{hilA}-sfGFP	This study
pMIB8516	P_{hilD}-*hilD* I215A-P_{hilA}-sfGFP	This study
pMIB8517	P_{hilD}-*hilD* S216A-P_{hilA}-sfGFP	This study
pMIB8518	P_{hilD}-*hilD* S217A-P_{hilA}-sfGFP	This study
pMIB8506	P_{hilD}-*hilD* N260A-P_{hilA}-sfGFP	This study
pMIB8507	P_{hilD}-*hilD* Q261A-P_{hilA}-sfGFP	This study
pMIB8304	P_{hilD}-*hilD* K264A-P_{hilA}-sfGFP	This study
pMIB8597	P_{hilD}-*hilD* R267A-P_{hilA}-sfGFP	This study
pMIB8319	P_{hilD}-*hilD* V273A-P_{hilA}-sfGFP	This study
pMIB8411	P_{hilD}-*hilD* F303A-P_{hilA}-sfGFP	This study
pMIB8602	P_{hilD}-*hilD* N86S-P_{hilA}-sfGFP	This study
pMIB8603	P_{hilD}-*hilD* R30Q-P_{hilA}-sfGFP	This study
pMIB8604	P_{hilD}-*hilD* A124S-P_{hilA}-sfGFP	This study
pMIB8605	P_{hilD}-*hilD* S220G-P_{hilA}-sfGFP	This study
pMIB8606	P_{hilD}-*hilD* V40I-P_{hilA}-sfGFP	This study
pMIB8607	P_{hilD}-*hilD* A275S-P_{hilA}-sfGFP	This study
pMIB8608	P_{hilD}-*hilD* A110D-P_{hilA}-sfGFP	This study
pMIB8609	P_{hilD}-*hilD* D72E-P_{hilA}-sfGFP	This study
pMIB8601	P_{hilD}-*hilD* K169Q-P_{hilA}-sfGFP	This study
pMIB8610	P_{hilD}-*hilD* A130V-P_{hilA}-sfGFP	This study
pMIB9041	P_{hilD}-*hilD* V228I-P_{hilA}-sfGFP	This study
pMIB9042	P_{hilD}-*hilD* T226P-P_{hilA}-sfGFP	This study
pMIB9043	P_{hilD}-*hilD* V125I-P_{hilA}-sfGFP	This study
pMIB9044	P_{hilD}-*hilD* T127V-P_{hilA}-sfGFP	This study
pMIB9045	P_{hilD}-*hilD* S164N-P_{hilA}-sfGFP	This study
pMIB9046	P_{hilD}-*hilD* N165S-P_{hilA}-sfGFP	This study
pMIB9047	P_{hilD}-*hilD* G162S-P_{hilA}-sfGFP	This study
pMIB9048	P_{hilD}-*hilD* V125K-P_{hilA}-sfGFP	This study
pMIB9049	P_{hilD}-*hilD* P137S-P_{hilA}-sfGFP	This study
pMIB9050	P_{hilD}-*hilD* K157M-P_{hilA}-sfGFP	This study
pHilC	pET-21a(+) with *hilC* cloned at NdeI and NotI sites. Synthesised by Synbio Technologies. N-terminal His_6 tag with TEV cleavage site; Amp^R	This study

pSUMO-HilD	pET-24a(+) with *hilD* cloned at NdeI and NotI sites. N-terminal His_6-SUMO tag; Kan^R	(*94*)
pHilD_L45A	pSUMO-HilD with L45A. Made by site-directed-mutagenesis of pSUMO-HilD using the following primers: L45A_f / L45A_r	This study
pHilD_Y46A	pSUMO-HilD with Y46A. Made by site-directed-mutagenesis of pSUMO-HilD using the following primers: Y46A_f / Y46A_r	This study
pHilD_I100A	pSUMO-HilD with I100A. Made by site-directed-mutagenesis of pSUMO-HilD using the following primers: I100A_f / I100A_r	This study
pHilD_Y212A	pSUMO-HilD with Y212A. Made by site-directed-mutagenesis of pSUMO-HilD using the following primers: Y212A_f / Y212A_r	This study

Table S10.
List of primers.

Primers	Sequence 5’-3’
gib_uni_890_f2	CAAGCTCAATAAAAAGCCCCAC
gib_uni_890_r2	CAAGAGGGTCATTATATTTCGCG
gib_890_HiBiT_a_f	TTCCGCGAAATATAATGACCCTCTTGACGCCGCCAAATGCTCCGGTC
gib_HiBit_Ig53_b_r	GCTAATCTTCTTGAACAGCCGCCAGCCGCTCACCTTCACCACGCTTTCTTCCGCCGC
gib_HiBit_Ig53_c_f	GTGAGCGGCTGGCGGCTGTTCAAGAAGATTAGCGTGACAGCCTATAGTATTACATTG
gib_890_HiBiT_d_r	GCGGTGGGGCTTTTTATTGAGCTTGATCAATATCGACGTCATCCT
gib_890_dsiiF_a_f	TGTTATTCCGCGAAATATAATGACCCTCTTGCTTTACGCCAGGTACACCG
gib_dsiiF_b_r	CCACCTGATAACAGCGACAAGCGCTGCTTATTAAGTAAACCCCCTCACCC
gib_dsiiF_c_f	TCACCTTTGGGTGAGGGGGTTTACTTAATAAGCAGCGCTTGTCGC
gib_890_dsiiF_d_r	CCACCGCGGTGGGGCTTTTTATTGAGCTTGTCTTTCGCATACCAGGCAGG
gib_890_ssiF_a_f	CGCGAAATATAATGACCCTCTTGTCAAGGGTGATGTTACTACTGGCGC
gib_FLAG_siiF_b_r	ATCGATGTCATGATCTTTATAATCACCGTCATGGTCTTTGTAGTCCATTAATAATTTATCCGGAGAAC
gib_FLAG_siiF_c_f	GATTATAAAGATCATGACATCGATTACAAGGATGACGATGACAAATAAAATAAGCAGCGCTTGTCGCTG
gib_890_ssiF_d_r	GTGGGGCTTTTTATTGAGCTTGATCTCTTTCGCATACCAGGCAGGAC
gib_HiBit_FLAG2_f	AGCGGCTGGCGGCTGTTCAAGAAGATTAGCTCTAGAGACTACAAAGACCATGAC
gib_HiBit_SseFc_r	TCTTCTTGAACAGCCGCCAGCCGCTCACTCCACTCGAACCTGGTTCTCCCCGAGATGTATG
gib_nSD_sscB_Fwd	ATAGGTATGATGATGAAAGAAGATCAGAAAAATAAAATACCC
gib_pT10_KO_rha_Rv	TTAAGCACTAGGCCTCAGATCC
gib_pT10-KO-rha_ProsseA_f	GGATCTGAGGCCTAGTGCTTAATAGAAGAGAACAACGGCAAG
gib_ProsseA_nSD-SscB_r	GGGTATTTTATTTTTCTGATCTTCTTTCATCATCATACCTATACGATAGATAATTAACGTGC
gib_890_hilA_a_f	GTTATTCCGCGAAATATAATGACCCTCTTGATCTCCTTCCGGCTTTAACC

gib_890_hilA_b_r	AATAATGCATATCTCCTCTCTCAGATTGATAATAGTGTAT TCTCTTACAGGG
gib_890_hilA_c_f	CTTTTCACCCTGTAAGAGAATACACTATTATCAATCTGAG AGAGGAGATATGC
gib_890_hilA_d_r	GCGGTGGGGCTTTTTATTGAGCTTGAGAATACCTGGCGGA TAGGG
gib_890_rtsAB_a_f	GTTATTCCGCGAAATATAATGACCCTCTTGCCATAATGAT GCGTTGTTCG
gib_890_rtsA_b_r	AGATAAAAACGCTAAAAATTCCGATGGTGTTTGTAAACAT TCAATGCTCCC
gib_890_rtsA_c_f	GTCCAGGTGGGGAGCATTGAATGTTTACAAACACCATCG GAATTTTTAGCG
gib_890_rtsA_d_r	GCGGTGGGGCTTTTTATTGAGCTTGATATTTGCGGCAGCG TAGTC
gib_890_hilC_a_f	GTTATTCCGCGAAATATAATGACCCTCTTGCCAAAAACTG ATGGTGTTGC
gib_890_hilC _b_r	GATAGTAACGTTTAAAATAATTTCACAAAATTTTATCCTG TGTGCTATAAGG
gib_890_hilC _c_f	ATAGCACACAGGATAAAATTTTGTGAAATTATTTTAAACG TTACTATCTG
gib_890_hilC _d_r	GCGGTGGGGCTTTTTATTGAGCTTGATTCATTCCTACCGC AATCG
gib_890_siiF_a_f	CGCGAAATATAATGACCCTCTTGTCAAGGGTGATGTTACT ACTGGCGC
gib_FLAG_siiF_b_r	ATCGATGTCATGATCTTTATAATCACCGTCATGGTCTTTGT AGTCCATTAATAATTTATCCGGAGAAC
gib_FLAG_siiF_c_f	GATTATAAAGATCATGACATCGATTACAAGGATGACGAT GACAAATAAAATAAGCAGCGCTTGTCGCTG
gib_890_siiF_d_r	GTGGGGCTTTTTATTGAGCTTGATCTCTTTCGCATACCAGG CAGGAC
gib_890_hilA::sfGF P_b_r	CGGTGAACAGTTCTTCACCTTTAGACATGATAATAGTGTA TTCTCTTACAGGG
gib_890_hilA::sfGF P_c_f	TCACGCACGGCATGGATGAGCTCTACAAATAAAATCTGA GAGAGGAGATATGC
gib_sfGFP_f	TCTAAAGGTGAAGAACTGTTC
gib_sfGFP_r	TTATTTGTAGAGCTCATCCATG
QC_hilA_SD-B_f	CTTTTCACCCTGTAAGAGAAATTATCATGTCTAAAGGTGA AGAAC
QC_hilA_SD-B_r	GTTCTTCACCTTTAGACATGATAATTTCTCTTACAGGGTGA AAAG
gib890_dSPI1_a_f	CGCGAAATATAATGACCCTCTTGTCTGTTAGCCAACCGTC GAC
dSPI1_b_r	GCGATTCGATAACAATGCCGT
dSPI1invH_bc136f	ACGGCATTGTTATCGAATCGCTACTTGCTGCCCATGAAAG AC

dSPI1_d_r	GCGGTGGGGCTTTTTATTGAGCTTGATTCATCGCCTGGAG CTTCAAA
g_p890_hilD_A_f	CGCGAAATATAATGACCCTCTTG GATAGAGATACGCTTATTTTCTTCG
g_KO_hilD_A_r	CTTAAGTGACAGATACAAAAAATG ATTATCCCTTTGTTGATGTTATTTTAATG
g_KO_hilD_D_f	CATTAAAATAACATCAACAAAGGGATAAT CATTTTTTGTATCTGTCACTTAAG
g_p890_hilD_D_r	GTGGGGCTTTTTATTGAGCTTG ACGGTCAGGTTGAGCTTTTATTATG
PhilA_A1_f	[Cyanine5]GGGAGTAAAGAAAAGACGATATCATTATTTTGC AAAAAAATATAAAAATAAGCGCACCATTA
PhilA_A1_r	TAATGGTGCGCTTATTTTTATATTTTTTTGCAAAATAATGA TATCGTCTTTTCTTTACTCCC
gib_uni_pT12_f	AGCTTGGCTGTTTTGGCGGATG
gib_uni_pT12_r	GGTGAATTCCTCCTGAATTTC
gib_pT12_hilD_f	GAAATTCAGGAGGAATTCACCATGGAAAATGTAACCTTT GTAAGTAATAG
gib_pT12_hilD_r	CATCCGCCAAAACAGCCAAGCTTTAATGGTTCGCCATTTT TATG
gib_pT12_hilC_f	GTCGTAATGAAATTCAGGAGGAATTCACCATGGTATTGCC TTCAATGAATAAATC
gib_pT12_hilC_r	ATCTTCTCTCATCCGCCAAAACAGCCAAGCTTCAATGGTT CATTGTACGCATAAAG
gib_pT12_rtsA_f	GGTCGTAATGAAATTCAGGAGGAATTCACCATGCTAAAA GTATTTAATCCCTCAC
gib_pT12_rtsA_r	TCTTCTCTCATCCGCCAAAACAGCCAAGCTTCAATTAACA TATTGATGACGAGAG
gib_uni_pT10_rep_r	TCAGATCCTTCCGTATTTAGCCAG
gib_PhilD_pT10_f	GAACATACTGGCTAAATACGGAAGGATCTGAATATACTG TTAGCGATGTCTG
gib_sfGFP_pT10_rrnB_r	TTCTCTCATCCGCCAAAACAGCCAAGCTTTATTTGTAGAG CTCATCCATG
gib_PhilA_rep_f	GAACATACTGGCTAAATACGGAAGGATCTGAATCTCCTTC CGGCTTTAAC
gib_rep_PhilA_r	AATCCACAGGGTTAAAGCCGGAAGGAGATTCAGATCCTT CCGTATTTAGC
QC_hilD_Q31A_f	TTAAAATCACTTTTGACAAATACCCGGGCGCAAATTAAAA GTCAGACTCAGCAGG
QC_hilD_Q31A_r	TGCTGAGTCTGACTTTTAATTTGCGCCCGGGTATTTGTCAA AAGTGATTTTAATTTCTG
QC_hilD_I42A_f	GTCAGACTCAGCAGGTTACCGCCAAAAATCTTTATGTAAG
QC_hilD_I42A_r	CTTACATAAAGATTTTTGGCGGTAACCTGCTGAGTCTGAC
QC_hilD_L45A_f	CAGCAGGTTACCATCAAAAATGCTTATGTAAGCAGTTTCA C

QC_hilD_L45A_r	GTGAAACTGCTTACATAAGCATTTTTGATGGTAACCTGCTG
QC_hilD_Y46A_f	CAGGTTACCATCAAAAATCTTGCTGTAAGCAGTTTCACTTTAG
QC_hilD_Y46A_r	CTAAAGTGAAACTGCTTACAGCAAGATTTTTGATGGTAACCTG
QC_hilD_S48A_f	CCATCAAAAATCTTTATGTAGCCAGTTTCACTTTAGTTTGCTTTC
QC_hilD_S48A_r	GAAAGCAAACTAAAGTGAAACTGGCTACATAAAGATTTTTGATGG
QC_hilD_T51A_f	CATCAAAAATCTTTATGTAAGCAGTTTCGCTTTAGTTTGCTTTCGGAGCGGTAAAC
QC_hilD_T51A_r	GTTTACCGCTCCGAAAGCAAACTAAAGCGAAACTGCTTACATAAAGATTTTTGATG
QC_hilD_V53A_f	CTTTATGTAAGCAGTTTCACTTTAGCTTGCTTTCGGAGCGGTAAAC
QC_hilD_V53A_r	GTTTACCGCTCCGAAAGCAAGCTAAAGTGAAACTGCTTACATAAAG
QC_hilD_L60A_f	GTTTGCTTTCGGAGCGGTAAAGCGACGATTAGCAATAATCAC
QC_hilD_L60A_r	GTGATTATTGCTAATCGTCGCTTTACCGCTCCGAAAGCAAAC
QC_hilD_L79A_f	CTGTGACGAACCTGGGATGTTGGTGGCCAAAAAAGAGCAGGTAGTTAACG
QC_hilD_L79A_r	CGTTAACTACCTGCTCTTTTTTGGCCACCAACATCCCAGGTTCGTCACAG
QC_hilD_V85A_f	GTGCTCAAAAAAGAGCAGGTAGCTAACGTGACGCTTGAAGAGGT
QC_hilD_V85A_r	ACCTCTTCAAGCGTCACGTTAGCTACCTGCTCTTTTTTGAGCAC
QC_hilD_V87A_f	CAAAAAAGAGCAGGTAGTTAACGCGACGCTTGAAGAGGTCAATG
QC_hilD_V87A_r	CATTGACCTCTTCAAGCGTCGCGTTAACTACCTGCTCTTTTTTG
QC_hilD_L89A_f	GAGCAGGTAGTTAACGTGACGGCTGAAGAGGTCAATGGCCAC
QC_hilD_L89A_r	GTGGCCATTGACCTCTTCAGCCGTCACGTTAACTACCTGCTC
QC_hilD_F98A_f	GAGGTCAATGGCCACATGGATGCCGATATACTCGAGATACCGAC
QC_hilD_F98A_r	GTCGGTATCTCGAGTATATCGGCATCCATGTGGCCATTGACCTC
QC_hilD_I100A_f	CAATGGCCACATGGATTTCGATGCACTCGAGATACCGACGCAAC
QC_hilD_I100A_r	GTTGCGTCGGTATCTCGAGTGCATCGAAATCCATGTGGCCATTG

QC_hilD_L101A_f	GGCCACATGGATTTCGATATAGCCGAGATACCGACGCAACGAC
QC_hilD_L101A_r	GTCGTTGCGTCGGTATCTCGGCTATATCGAAATCCATGTGGCC
QC_hilD_E102A_f	TGGCCACATGGATTTCGATATACTCGCAATACCGACGCAACGACTTGGCGCTCTC
QC_hilD_E102A_r	AGAGCGCCAAGTCGTTGCGTCGGTATTGCGAGTATATCGAAATCCATGTGGCCATTG
QC_hilD_Y212A_f	GATAACGTTAAAGGAGCGCGTTGCCAACATTATATCTTCGTCACC
QC_hilD_Y212A_r	GGTGACGAAGATATAATGTTGGCAACGCGCTCCTTTAACGTTATC
QC_hilD_I214A_f	CGTTAAAGGAGCGCGTTTACAACGCTATATCTTCGTCACCCAGTAG
QC_hilD_I214A_r	CTACTGGGTGACGAAGATATAGCGTTGTAAACGCGCTCCTTTAACG
QC_hilD_I215A_f	GTTAAAGGAGCGCGTTTACAACATTGCATCTTCGTCACCCAGTAGAC
QC_hilD_I215A_r	GTCTACTGGGTGACGAAGATGCAATGTTGTAAACGCGCTCCTTTAAC
QC_hilD_S216A_f	GAGCGCGTTTACAACATTATAGCTTCGTCACCCAGTAGACAGTGG
QC_hilD_S216A_r	CCACTGTCTACTGGGTGACGAAGCTATAATGTTGTAAACGCGCTC
QC_hilD_S217A_f	CGCGTTTACAACATTATATCTGCGTCACCCAGTAGACAGTGGAAG
QC_hilD_S217A_r	CTTCCACTGTCTACTGGGTGACGCAGATATAATGTTGTAAACGCG
QC_hilD_N260A_f	CATCTACTTATCGGCAAGAATGGCTCAGGCAGCAAAACTTTTACG
QC_hilD_N260A_r	CGTAAAAGTTTTGCTGCCTGAGCCATTCTTGCCGATAAGTAGATG
QC_hilD_Q261A_f	CTTATCGGCAAGAATGAATGCGGCAGCAAAACTTTTACGCATAG
QC_hilD_Q261A_r	CTATGCGTAAAAGTTTTGCTGCCGCATTCATTCTTGCCGATAAG
QC_hilD_K264A_f	ATCGGCAAGAATGAATCAGGCAGCAGCGCTTTTACGCATAGGCAACCATAATG
QC_hilD_K264A_r	CATTATGGTTGCCTATGCGTAAAAGCGCTGCTGCCTGATTCATTCTTGCCGATAAG
QC_hilD_R267A_f	GAATCAGGCAGCAAAACTTTTAGCCATAGGCAACCATAATGTTAATGCTGTAGC
QC_hilD_R267A_r	ACAGCATTAACATTATGGTTGCCTATGGCTAAAAGTTTTGCTGCCTGATTCATTC
QC_hilD_V273A_f	CGCATAGGCAACCATAATGCGAATGCTGTAGCATTAAAATGTGGT

QC_hilD_V273A_r	ACCACATTTTAATGCTACAGCATTCGCATTATGGTTGCCT ATGCG
QC_hilD_F303A_f	ATATTTTAAAACTACGCCATCGACAGCTATAAAAATGGCG AACCATTAAATCTCCTTC
QC_hilD_F303A_r	AGATTTAATGGTTCGCCATTTTTATAGCTGTCGATGGCGT AGTTTTAAAATATTTTTTG
QC_hilD_N86S_f	GTTGGTGCTCAAAAAAGAGCAGGTAGTTTCCGTGACGCTT GAAGAGGTCAATGGCC
QC_hilD_N86S_r	GGCCATTGACCTCTTCAAGCGTCACGGAAACTACCTGCTC TTTTTTGAGCACCAAC
QC_hilD_R30Q_f	CAGAAATTAAAATCACTTTTGACAAATACCCAGCAGCAA ATTAAAAGTCAGACTCAGC
QC_hilD_R30Q_r	GCTGAGTCTGACTTTTAATTTGCTGCTGGGTATTTGTCAAA AGTGATTTTAATTTCTG
QC_hilD_A124S_f	CCCAAACGAGCAGCAAACCAAAATGTCGGTACCCACAGA GAAAGCGCAGAAGATC
QC_hilD_A124S_r	GATCTTCTGCGCTTTCTCTGTGGGTACCGACATTTTGGTTT GCTGCTCGTTTGGG
QC_hilD_S220G_f	GTTTACAACATTATATCTTCGTCACCCGGTAGACAGTGGA AGCTTACGGATGTTG
QC_hilD_S220G_r	CAACATCCGTAAGCTTCCACTGTCTACCGGGTGACGAAGA TATAATGTTGTAAAC
QC_hilD_V40I_f	GCAGCAAATTAAAAGTCAGACTCAGCAGATTACCATCAA AAATCTTTATGTAAGCAG
QC_hilD_V40I_r	CTGCTTACATAAAGATTTTTGATGGTAATCTGCTGAGTCT GACTTTTAATTTGCTGC
QC_hilD_A275S_f	CGCATAGGCAACCATAATGTTAATTCTGTAGCATTAAAAT GTGGTTATGATAGC
QC_hilD_A275S_r	GCTATCATAACCACATTTTAATGCTACAGAATTAACATTA TGGTTGCCTATGCG
QC_hilD_A110D_f	GAGATACCGACGCAACGACTTGGCGATCTCTATGCACTTA TCCCAAACGAG
QC_hilD_A110D_r	CTCGTTTGGGATAAGTGCATAGAGATCGCCAAGTCGTTGC GTCGGTATCTC
QC_hilD_D72E_f	GCAATAATCACGATACGATTTACTGTGAGGAACCTGGGAT GTTGGTGCTC
QC_hilD_D72E_r	GAGCACCAACATCCCAGGTTCCTCACAGTAAATCGTATCG TGATTATTGC
QC_hilD_K169Q_f	CAAGCAAAGGTTGCAGTAACTGTAACAACCAAAGTTGTA TTGAAAATGAAGAG
QC_hilD_K169Q_r	CTCTTCATTTTCAATACAACTTTGGTTGTTACAGTTACTGC AACCTTTGCTTG
QC_hilD_A130V_f	CAAAATGGCGGTACCCACAGAGAAAGTGCAGAAGATCTT CTATACGCCTGACTTTC
QC_hilD_A130V_r	GAAAGTCAGGCGTATAGAAGATCTTCTGCACTTTCTCTGT GGGTACCGCCATTTTG

QC_hilD_V228I_f	CCCAGTAGACAGTGGAAGCTTACGGATATTGCCGATCATATATTTATGAGTAC
QC_hilD_V228I_r	GTACTCATAAATATATGATCGGCAATATCCGTAAGCTTCCACTGTCTACTGGG
QC_hilD_T226P_f	CACCCAGTAGACAGTGGAAGCTTCCGGATGTTGCCGATCATATATTTATG
QC_hilD_T226P_r	CATAAATATATGATCGGCAACATCCGGAAGCTTCCACTGTCTACTGGGTG
QC_hilD_V125I_f	CCCAAACGAGCAGCAAACCAAAATGGCGATACCCACAGAGAAAGCGCAGAAGATC
QC_hilD_V125I_r	GATCTTCTGCGCTTTCTCTGTGGGTATCGCCATTTTGGTTTGCTGCTCGTTTGGG
QC_hilD_T127V_f	GCAGCAAACCAAAATGGCGGTACCCGTAGAGAAAGCGCAGAAGATCTTCTATAC
QC_hilD_T127V_r	GTATAGAAGATCTTCTGCGCTTTCTCTACGGGTACCGCCATTTTGGTTTGCTGC
QC_hilD_S164N_f	GTACGAAGGATACAAGCAAAGGTTGCAATAACTGTAACAACAAAAGTTGTATTG
QC_hilD_S164N_r	CAATACAACTTTTGTTGTTACAGTTATTGCAACCTTTGCTTGTATCCTTCGTAC
QC_hilD_N165S_f	GAAGGATACAAGCAAAGGTTGCAGTTCCTGTAACAACAAAAGTTGTATTG
QC_hilD_N165S_r	CAATACAACTTTTGTTGTTACAGGAACTGCAACCTTTGCTTGTATCCTTC
QC_hilD_G162S_f	CTCCTGTACGAAGGATACAAGCAAAAGTTGCAGTAACTGTAACAACAAAAGTTG
QC_hilD_G162S_r	CAACTTTTGTTGTTACAGTTACTGCAACTTTTGCTTGTATCCTTCGTACAGGAG
QC_hilD_V125K_f	CCCAAACGAGCAGCAAACCAAAATGGCGAAACCCACAGAGAAAGCGCAGAAGATC
QC_hilD_V125K_r	GATCTTCTGCGCTTTCTCTGTGGGTTTCGCCATTTTGGTTTGCTGCTCGTTTGGG
QC_hilD_P137S_f	GAAAGCGCAGAAGATCTTCTATACGTCTGACTTTCCTGCCAGAAGAGAGG
QC_hilD_P137S_r	CCTCTCTTCTGGCAGGAAAGTCAGACGTATAGAAGATCTTCTGCGCTTTC
QC_hilD_K157M_f	CTGAAAACGGCGTTCTCCTGTACGATGGATACAAGCAAAGGTTGCAGTAACTG
QC_hilD_K157M_r	CAGTTACTGCAACCTTTGCTTGTATCCATCGTACAGGAGAACGCCGTTTTCAG
L45A_f	AATGCATATGTAAGCAGTTTCAC
L45A_r	TTTGATGGTAACCTGCTGAGT
Y46A_f	AATCTTGCAGTAAGCAGT
Y46A_r	TTTGATGGTAACCTGCTG
I100A_f	GATTTCGATGCACTCGAGATACCG

I100A_r	CATGTGGCCATTGACCTC
Y212A_f	GCGCGTTGCAAACATTATATCT
Y212A_r	TCCTTTAACGTTATCTGAGCCG

Data S1. RNA-seq source data (separate file).
RNA-seq read count data (N = 3 biological replicates) when bacteria were grown with DMSO (1%) as a control or C26 (100 µM). Source data of Fig. 2D and 2E are also included in the file.

Data S2. NMR Spectra of synthesized compounds.

***N*-(benzo[d][1,3]dioxol-5-ylmethyl)-2-(((5-bromothiophen-2-yl)methyl)(methyl)amino)acetamide (C26)**

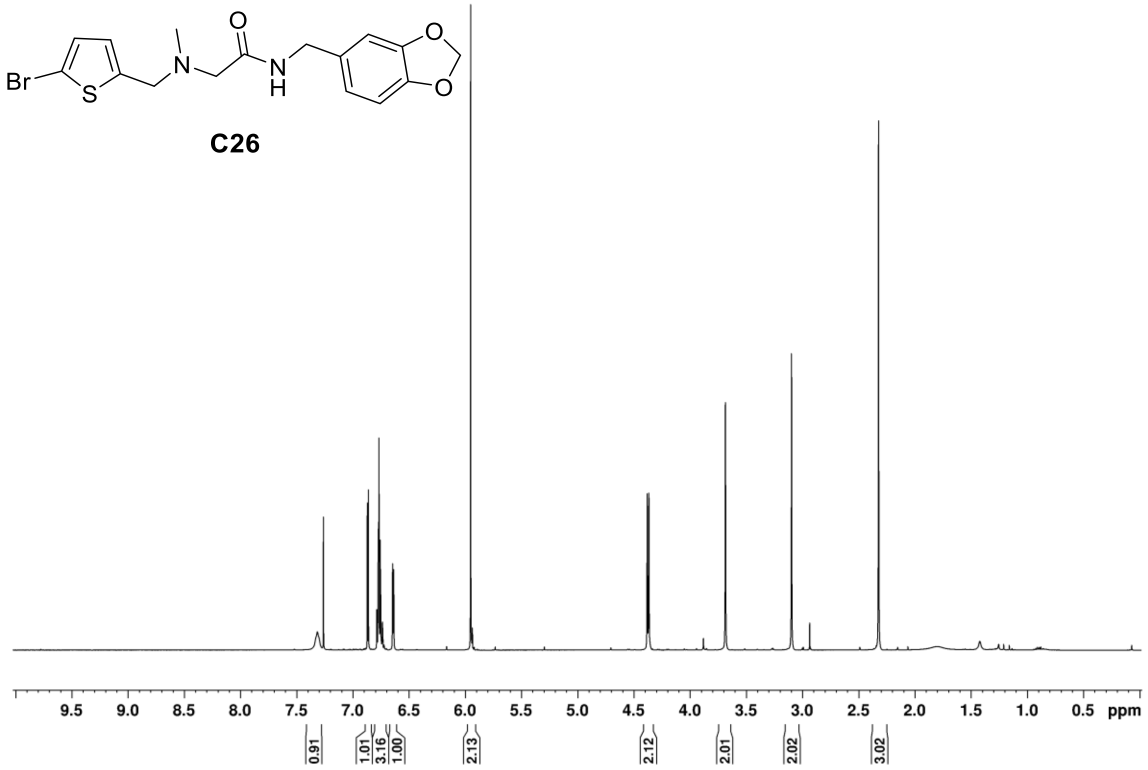

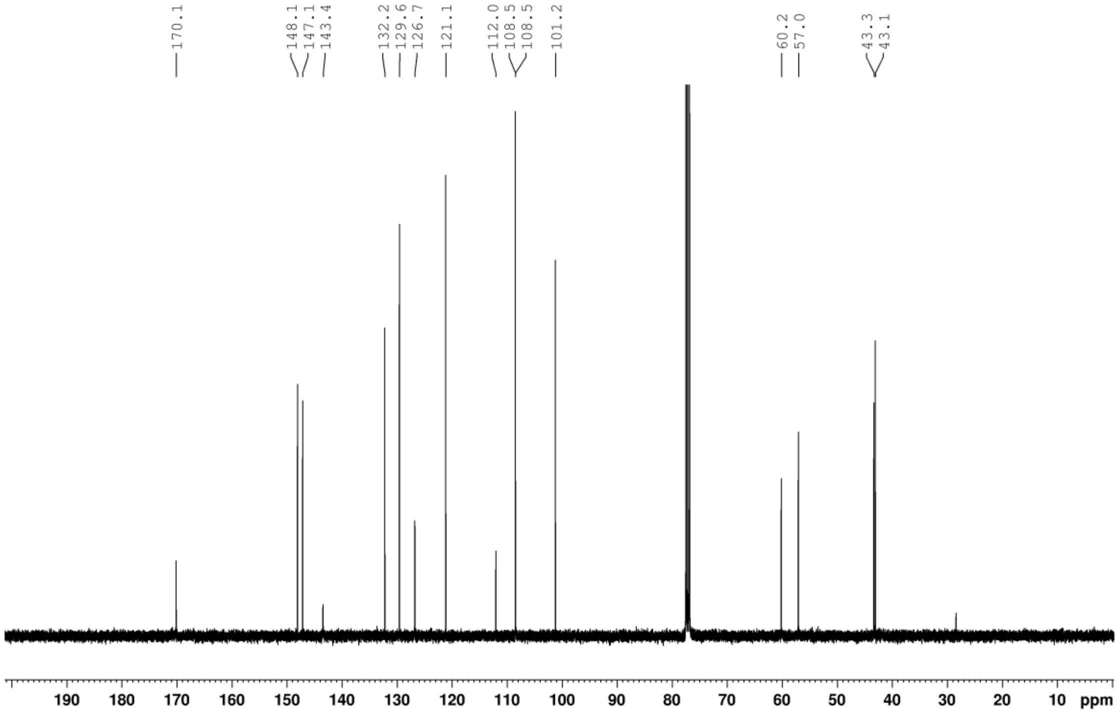

***N*-(benzo[d][1,3]dioxol-5-ylmethyl)-2-((2-bromobenzyl)(methyl)amino)acetamide (SW-C165)**

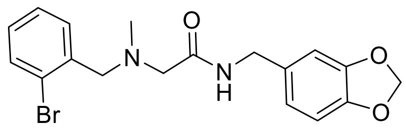

SW-C165

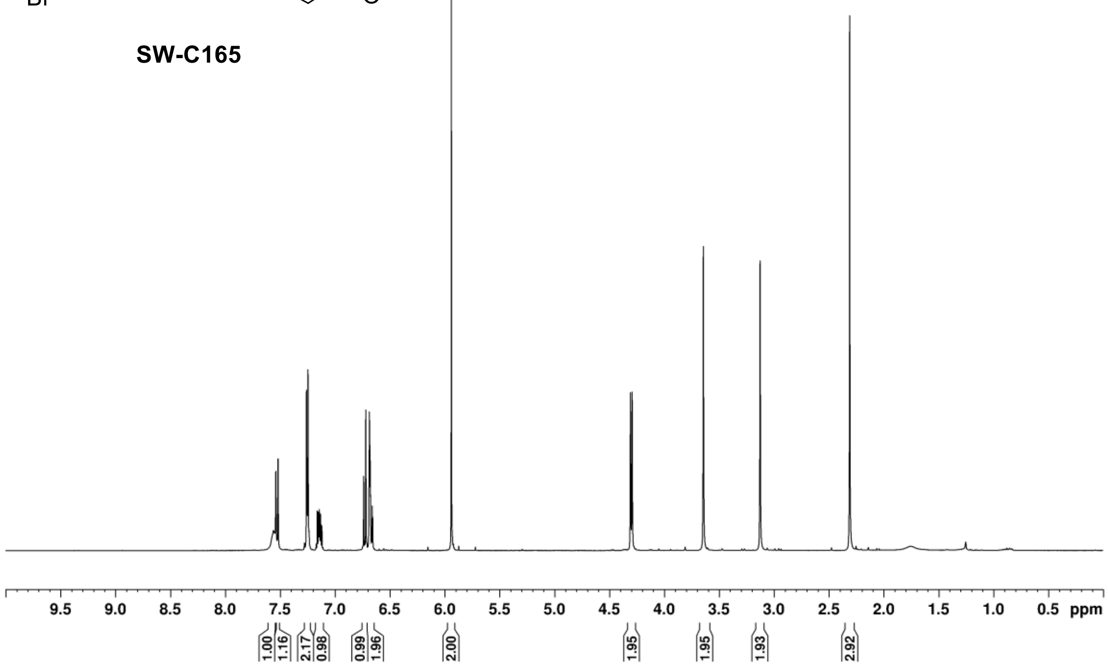

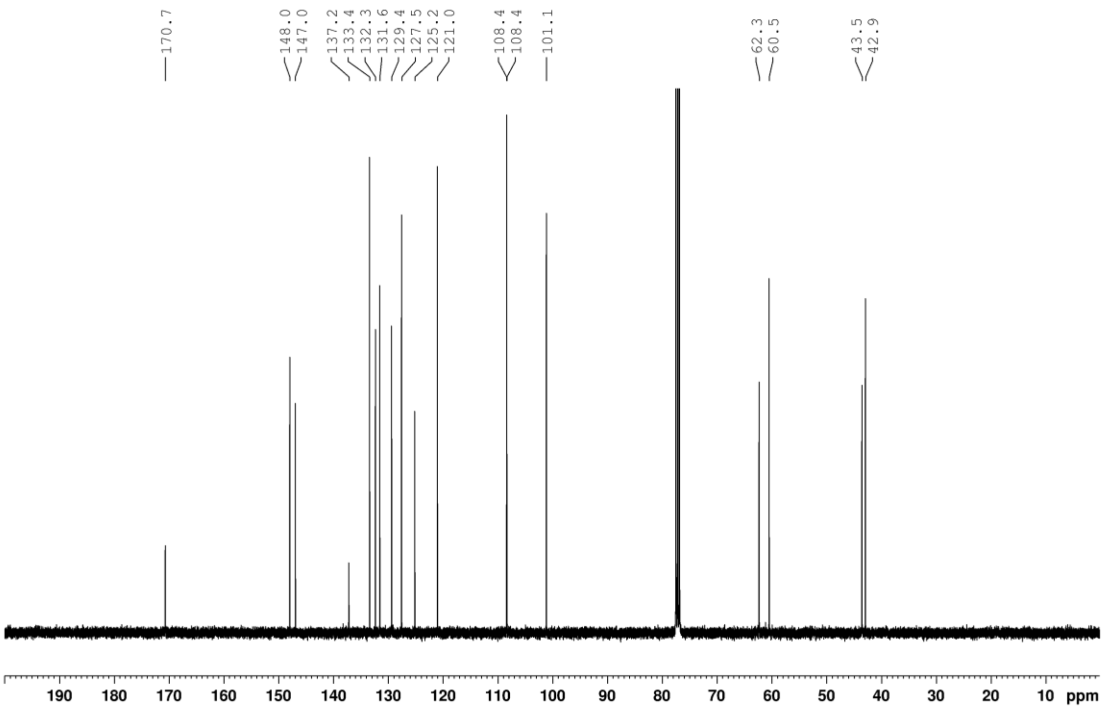

***N*-(benzo[d][1,3]dioxol-5-ylmethyl)-2-(((5-bromofuran-2-yl)methyl)(methyl)amino)acetamide (SW-C210)**

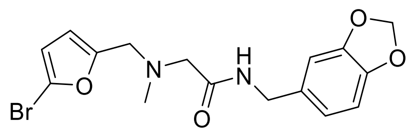

SW-C210

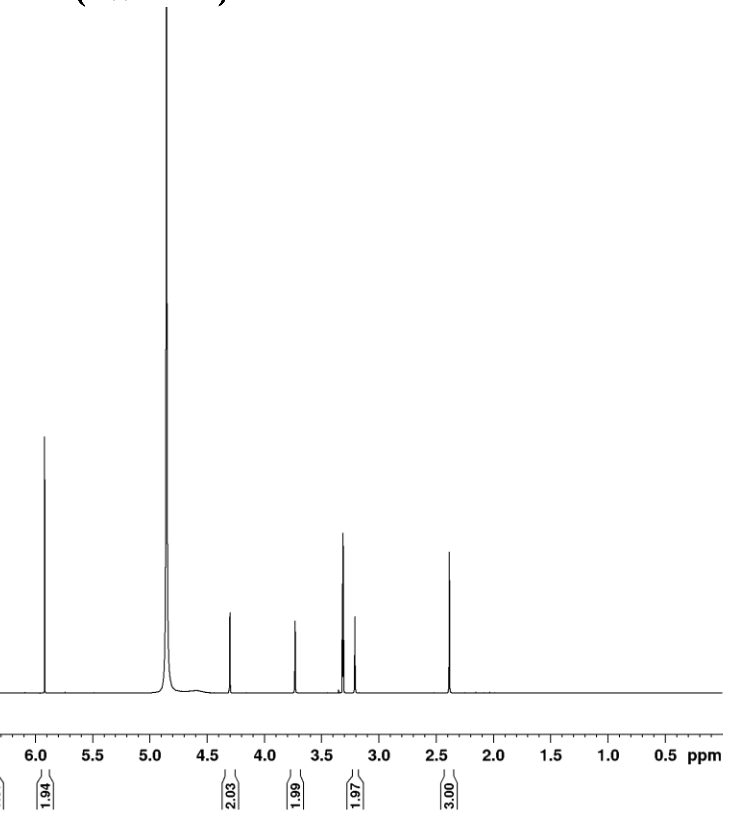

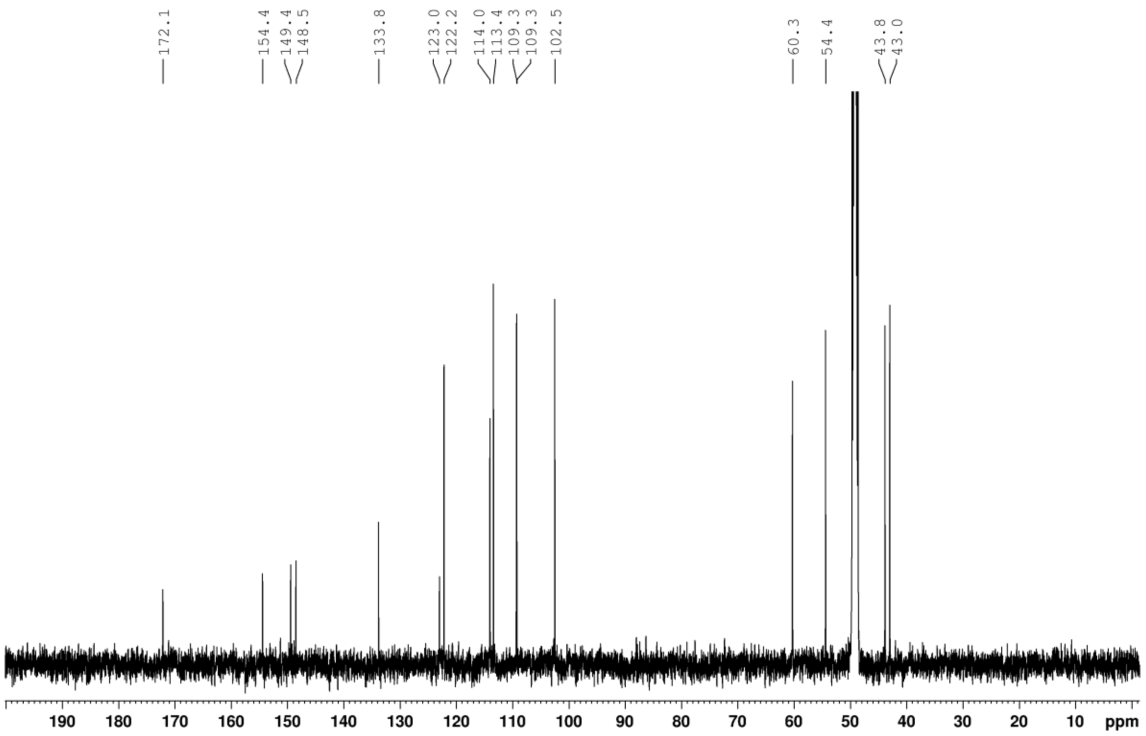

2-(((5-Bromothiophen-2-yl)methyl)(methyl)amino)-*N*-(3,5-dichlorobenzyl)acetamide (SW-C250)

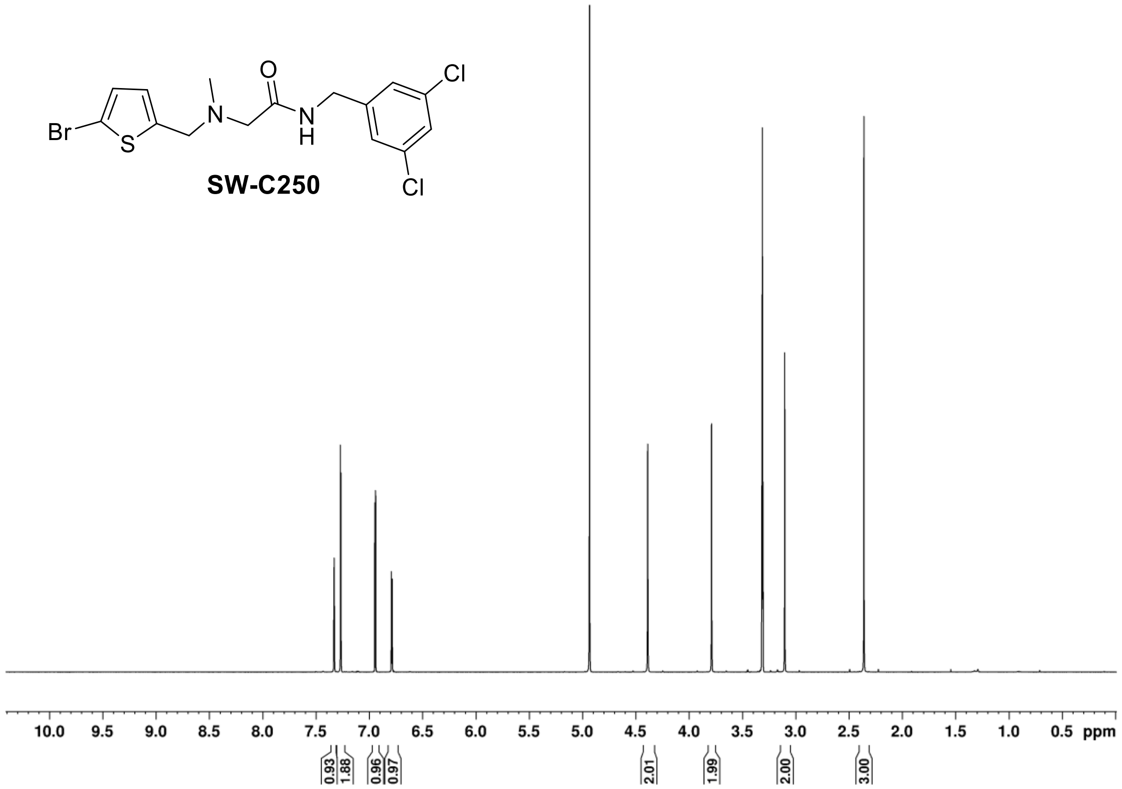

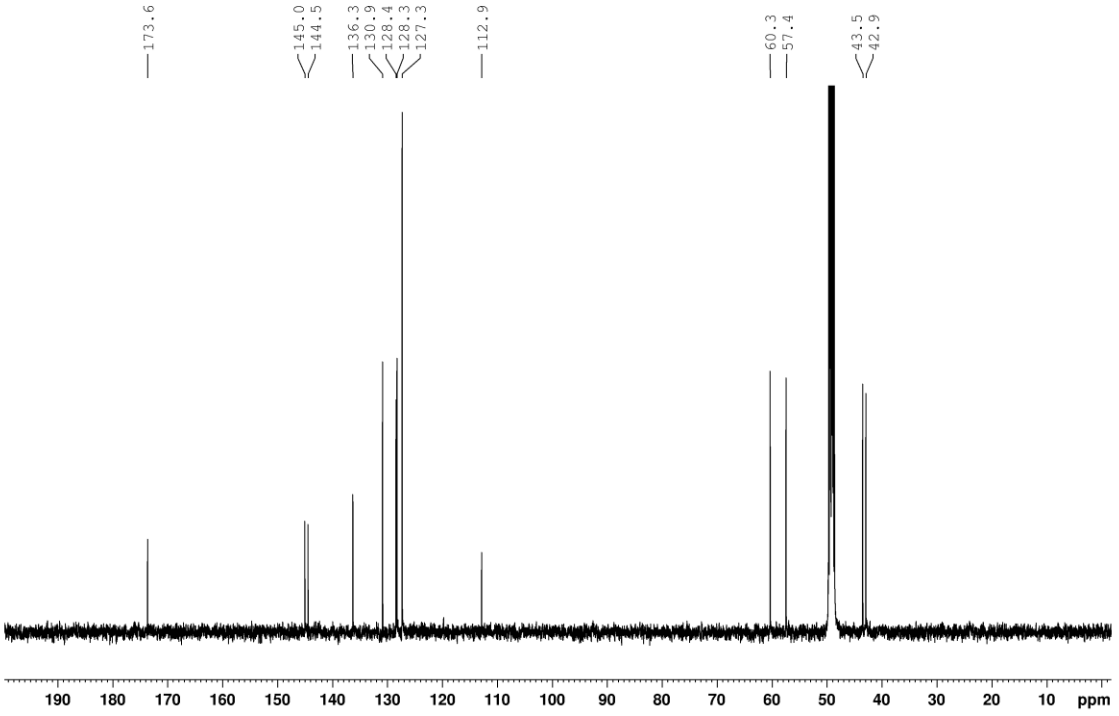

2-(((5-Bromothiophen-2-yl)methyl)(methyl)amino)-*N*-(4-chlorobenzyl)acetamide (SW-C202)

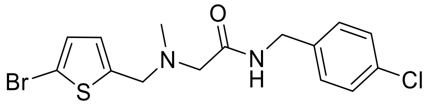

SW-C202

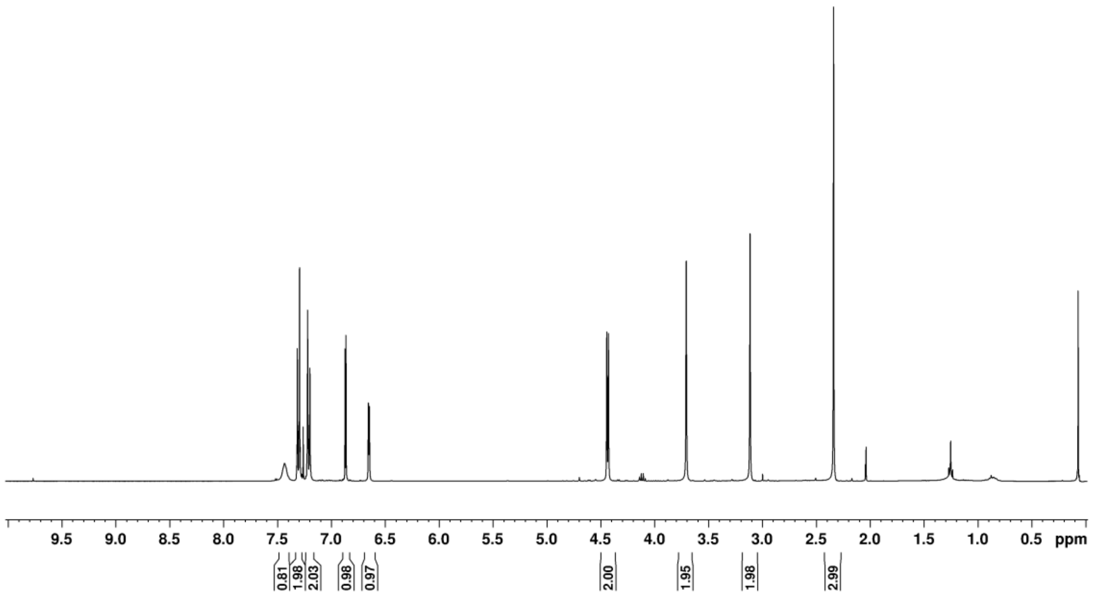

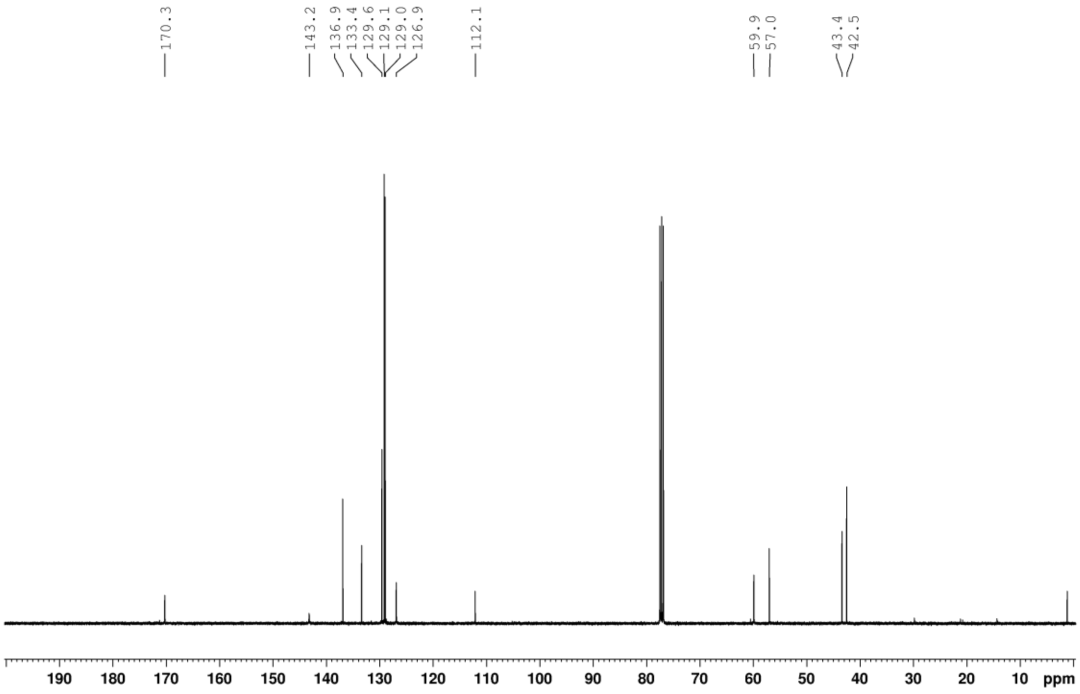

2-(((5-Bromothiophen-2-yl)methyl)(methyl)amino)-*N*-(3,4-dihydroxybenzyl)acetamide (SW-C170)

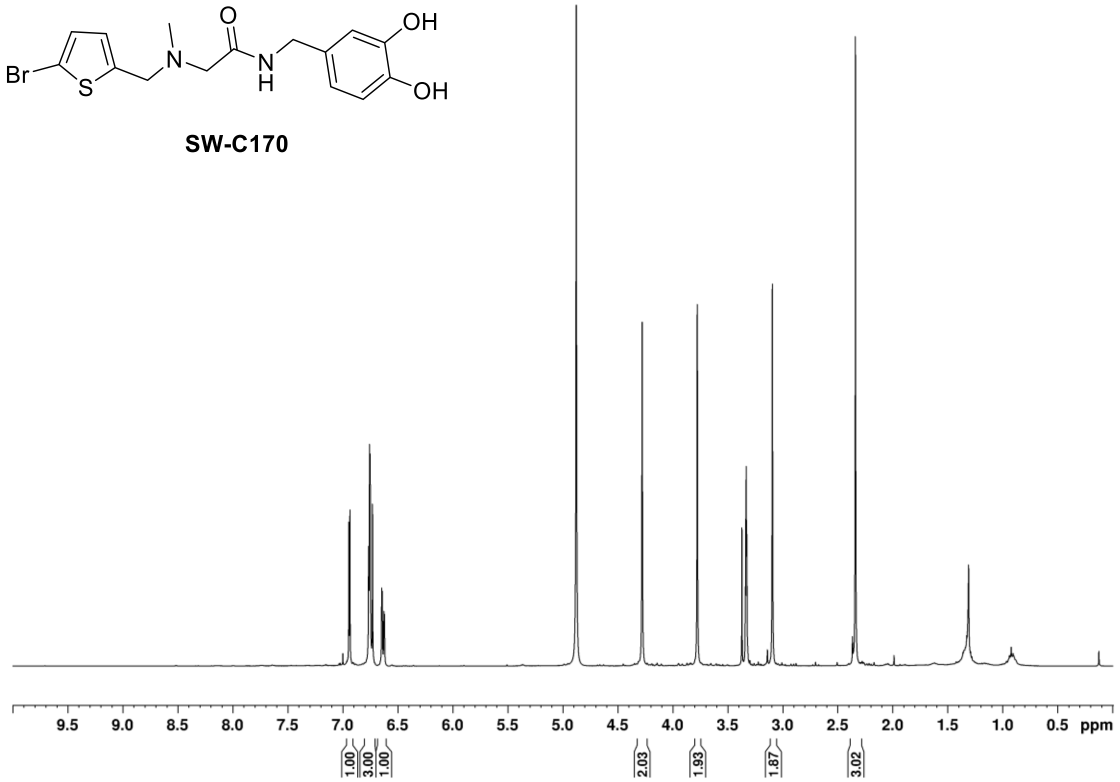

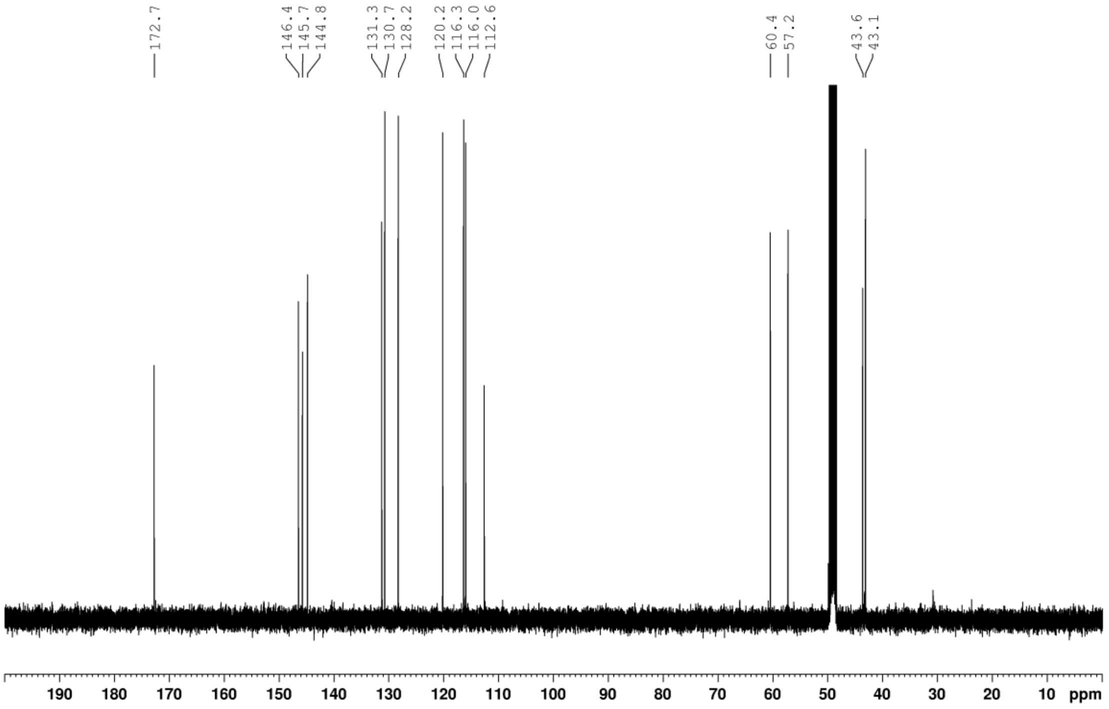

REFERENCES AND NOTES

1. Antimicrobial Resistance Collaborators, Global burden of bacterial antimicrobial resistance in 2019: A systematic analysis. *Lancet* **399**, 629–655 (2022).

2. S. W. Dickey, G. Y. C. Cheung, M. Otto, Different drugs for bad bugs: Antivirulence strategies in the age of antibiotic resistance. *Nat. Rev. Drug Discov.* **16**, 457–471 (2017).

3. J. H. Rex, H. Fernandez Lynch, I. G. Cohen, J. J. Darrow, K. Outterson, Designing development programs for non-traditional antibacterial agents. *Nat. Commun.* **10**, 3416 (2019).

4. U. Theuretzbacher, L. J. V. Piddock, Non-traditional antibacterial therapeutic options and challenges. *Cell Host Microbe* **26**, 61–72 (2019).

5. R. C. Allen, R. Popat, S. P. Diggle, S. P. Brown, Targeting virulence: Can we make evolution-proof drugs? *Nat. Rev. Microbiol.* **12**, 300–308 (2014).

6. M. E. Ohl, S. I. Miller, Salmonella: A model for bacterial pathogenesis. *Annu. Rev. Med.* **52**, 259–274 (2001).

7. A. Fàbrega, J. Vila, Salmonella enterica serovar Typhimurium skills to succeed in the host: Virulence and regulation. *Clin. Microbiol. Rev.* **26**, 308–341 (2013).

8. S. L. Marcus, J. H. Brumell, C. G. Pfeifer, B. B. Finlay, Salmonella pathogenicity islands: Big virulence in small packages. *Microbes Infect.* **2**, 145–156 (2000).

9. R. G. Gerlach, N. Cláudio, M. Rohde, D. Jäckel, C. Wagner, M. Hensel, Cooperation of Salmonella pathogenicity islands 1 and 4 is required to breach epithelial barriers. *Cell. Microbiol.* **10**, 2364–2376 (2008).

10. C. Wagner, B. Barlag, R. G. Gerlach, J. Deiwick, M. Hensel, The Salmonella enterica giant adhesin SiiE binds to polarized epithelial cells in a lectin-like manner. *Cell. Microbiol.* **16**, 962–975 (2014).

11. K. L. Main-Hester, K. M. Colpitts, G. A. Thomas, F. C. Fang, S. J. Libby, Coordinate regulation of Salmonella pathogenicity island 1 (SPI1) and SPI4 in Salmonella enterica serovar Typhimurium. *Infect. Immun.* **76**, 1024–1035 (2008).

12. V. Kuhle, M. Hensel, Cellular microbiology of intracellular Salmonella enterica: Functions of the type III secretion system encoded by Salmonella pathogenicity island 2. *Cell. Mol. Life Sci.* **61**, 2812–2826 (2004).

13. M. J. Worley, G. S. Nieman, K. Geddes, F. Heffron, *Salmonella typhimurium* disseminates within its host by manipulating the motility of infected cells. *Proc. Natl. Acad. Sci. U.S.A.* **103**, 17915–17920 (2006).

14. Y. A. Golubeva, A. Y. Sadik, J. R. Ellermeier, J. M. Slauch, Integrating global regulatory input into the Salmonella pathogenicity island 1 type III secretion system. *Genetics* **190**, 79–90 (2012).

15. I. N. Olekhnovich, R. J. Kadner, DNA-binding activities of the HilC and HilD virulence regulatory proteins of *Salmonella enterica* serovar Typhimurium. *J. Bacteriol.* **184**, 4148–4160 (2002).

16. I. N. Olekhnovich, R. J. Kadner, Role of nucleoid-associated proteins Hha and H-NS in expression of *Salmonella enterica* activators HilD, HilC, and RtsA required for cell invasion. *J. Bacteriol.* **189**, 6882–6890 (2007).

17. K.-E. Narm, M. Kalafatis, J. M. Slauch, HilD, HilC, and RtsA form homodimers and heterodimers to regulate expression of the Salmonella pathogenicity island I type III secretion system. *J. Bacteriol.* **202**, e00012-20 (2020).

18. C. D. Ellermeier, J. R. Ellermeier, J. M. Slauch, HilD, HilC and RtsA constitute a feed forward loop that controls expression of the SPI1 type three secretion system regulator *hilA* in *Salmonella enterica* serovar Typhimurium. *Mol. Microbiol.* **57**, 691–705 (2005).

19. V. Bajaj, C. Hwang, C. A. Lee, *hilA* is a novel *ompR/toxR* family member that activates the expression of *Salmonella typhimurium* invasion genes: hilA *of* Salmonella typhimurium. *Mol. Microbiol.* **18**, 715–727 (1995).

20. L. M. Schechter, C. A. Lee, AraC/XylS family members, HilC and HilD, directly bind and derepress the Salmonella typhimurium hilA promoter. *Mol. Microbiol.* **40**, 1289–1299 (2001).

21. M. M. Banda, C. Zavala-Alvarado, D. Pérez-Morales, V. H. Bustamante, SlyA and HilD counteract H-NS-mediated repression on the *ssrAB* virulence operon of *Salmonella enterica* serovar Typhimurium and thus promote its activation by OmpR. *J. Bacteriol.* **201**, e0053018 (2019).

22. C. Smith, A. M. Stringer, C. Mao, M. J. Palumbo, J. T. Wade, Mapping the regulatory network for *Salmonella enterica* serovar typhimurium invasion. *mBio* **7**, e0102416 (2016).

23. M. Diard, V. Garcia, L. Maier, M. N. P. Remus-Emsermann, R. R. Regoes, M. Ackermann, W.-D. Hardt, Stabilization of cooperative virulence by the expression of an avirulent phenotype. *Nature* **494**, 353–356 (2013).

24. J. T. Pico-Rodríguez, H. Martínez-Jarquín, J. D. J. Gómez-Chávez, M. Juárez-Ramírez, L. C. Martínez-Chavarría, Effect of *Salmonella* pathogenicity island 1 and 2 (SPI-1 and SPI-2) deletion on intestinal colonization and systemic dissemination in chickens. *Vet. Res. Commun.* **48**, 49–60 (2024).

25. D. L. Hudson, A. N. Layton, T. R. Field, A. J. Bowen, H. Wolf-Watz, M. Elofsson, M. P. Stevens, E. E. Galyov, Inhibition of type III secretion in *Salmonella enterica* serovar Typhimurium by small-molecule inhibitors. *Antimicrob. Agents Chemother.* **51**, 2631–2635 (2007).

26. H. B. Felise, H. V. Nguyen, R. A. Pfuetzner, K. C. Barry, S. R. Jackson, M.-P. Blanc, P. A. Bronstein, T. Kline, S. I. Miller, An inhibitor of gram-negative bacterial virulence protein secretion. *Cell Host Microbe* **4**, 325–336 (2008).

27. D. Aiello, J. D. Williams, H. Majgier-Baranowska, I. Patel, N. P. Peet, J. Huang, S. Lory, T. L. Bowlin, D. T. Moir, Discovery and characterization of inhibitors of *Pseudomonas aeruginosa* type III secretion. *Antimicrob. Agents Chemother.* **54**, 1988–1999 (2010).

28. H. N. Lam, T. Lau, A. Lentz, J. Sherry, A. Cabrera-Cortez, K. Hug, A. Lalljie, J. Engel, R. S. Lokey, V. Auerbuch, Developing cyclic peptomers as broad-spectrum Gram-negative bacterial type III secretion system inhibitors. *Antimicrob. Agents Chemother.* **65**, e0169020 (2021).

29. E. M. Bosire, C. R. Eade, C. J. Schiltz, A. J. Reid, J. Troutman, J. S. Chappie, C. Altier, Diffusible signal factors act through AraC-type transcriptional regulators as chemical cues to repress virulence of enteric pathogens. *Infect. Immun.* **88**, e0022620 (2020).

30. C. N. Tsai, C. R. MacNair, M. P. T. Cao, J. N. Perry, J. Magolan, E. D. Brown, B. K. Coombes, Targeting two-component systems uncovers a small-molecule inhibitor of *Salmonella* virulence. *Cell Chem. Biol.* **27**, 793–805.e7 (2020).

31. Y. Wu, X. Yang, D. Zhang, C. Lu, Myricanol inhibits the type III secretion system of *Salmonella enterica* serovar Typhimurium by interfering with the DNA-binding activity of HilD. *Front. Microbiol.* **11**, 571217 (2020).

32. R. Chowdhury, P. D. P. Bitar, I. Keresztes, A. M. Condo Jr., C. Altier, A diffusible signal factor of the intestine dictates *Salmonella* invasion through its direct control of the virulence activator HilD. *PLOS Pathog.* **17**, e1009357 (2021).

33. Y. Shi, Z. Sun, Y. Liu, J. Shu, Y. Zhang, Q. Lv, J. Wang, X. Deng, H. Liu, J. Qiu, Inhibition of the type III secretion system of *Salmonella enterica* serovar Typhimurium via treatment with fraxetin. *Microbiol. Spectr.* **10**, e0294922 (2022).

34. Y. Shi, X. Chen, J. Shu, Y. Liu, Y. Zhang, Q. Lv, J. Wang, X. Deng, H. Liu, J. Qiu, Harmine, an inhibitor of the type III secretion system of *Salmonella enterica* serovar Typhimurium. *Front. Cell. Infect. Microbiol.* **12**, 967149 (2022).

35. X. Yang, K. R. Stein, H. C. Hang, Anti-infective bile acids bind and inactivate a *Salmonella* virulence regulator. *Nat. Chem. Biol.* **19**, 91–100 (2023).

36. S. Li, H. Liu, J. Shu, Q. Li, Y. Liu, H. Feng, J. Wang, X. Deng, Y. Zhang, Z. Guo, J. Qiu, Fisetin inhibits *Salmonella* Typhimurium type III secretion system regulator HilD and reduces pathology *in vivo*. *Microbiol. Spectr.* **12**, e0240623 (2024).

37. S. Westerhausen, M. Nowak, C. E. Torres-Vargas, U. Bilitewski, E. Bohn, I. Grin, S. Wagner, A NanoLuc luciferase-based assay enabling the real-time analysis of protein secretion and injection by bacterial type III secretion systems. *Mol. Microbiol.* **113**, 1240–1254 (2020).

38. S. V. Pais, S. Westerhausen, E. Bohn, S. Wagner, "Analysis of SPI-1 dependent type III secretion and injection using a NanoLuc luciferase-based assay" in *Bacterial Virulence*, O. Gal-Mor, Ed. (Springer US, 2022), vol. 2427, pp. 57–71.

39. J. D. Joiner, W. Steinchen, N. Mozer, T. Kronenberger, G. Bange, A. Poso, S. Wagner, M. D. Hartmann, HilE represses the activity of the *Salmonella* virulence regulator HilD *via* a mechanism distinct from that of intestinal long-chain fatty acids. *J. Biol. Chem.* **299**, 105387 (2023).

40. H. N. B. Moseley, E. V. Curto, N. R. Krishna, Complete relaxation and conformational exchange matrix (CORCEMA) analysis of NOESY spectra of interacting systems; two-dimensional transferred NOESY. *J. Magn. Reson. B* **108**, 243–261 (1995).

41. V. Jayalakshmi, N. R. Krishna, Complete relaxation and conformational exchange matrix (CORCEMA) analysis of intermolecular saturation transfer effects in reversibly forming ligand–receptor complexes. *J. Magn. Reson.* **155**, 106–118 (2002).

42. N. Rama Krishna, V. Jayalakshmi, Complete relaxation and conformational exchange matrix analysis of STD-NMR spectra of ligand–receptor complexes. *Prog. Nucl. Magn. Reson. Spectrosc.* **49**, 1–25 (2006).

43. H. Prochnow, V. Fetz, S.-K. Hotop, M. A. García-Rivera, A. Heumann, M. Brönstrup, Subcellular quantification of uptake in gram-negative bacteria. *Anal. Chem.* **91**, 1863–1872 (2019).

44. C. A. Lipinski, F. Lombardo, B. W. Dominy, P. J. Feeney, Experimental and computational approaches to estimate solubility and permeability in drug discovery and development settings. *Adv. Drug Deliv. Rev.* **46**, 3–26 (2001).

45. Y. A. Golubeva, J. R. Ellermeier, J. E. Cott Chubiz, J. M. Slauch, Intestinal long-chain fatty acids act as a direct signal to modulate expression of the *Salmonella* pathogenicity island 1 type iii secretion system. *mBio* **7**, e0217015 (2016).

46. R. Chowdhury, P. D. Pavinski Bitar, M. C. Adams, J. S. Chappie, C. Altier, AraC-type regulators HilC and RtsA are directly controlled by an intestinal fatty acid to regulate *Salmonella* invasion. *Mol. Microbiol.* **116**, 1464–1475 (2021).

47. M. J. Lowden, K. Skorupski, M. Pellegrini, M. G. Chiorazzo, R. K. Taylor, F. J. Kull, Structure of *Vibrio cholerae* ToxT reveals a mechanism for fatty acid regulation of virulence genes. *Proc. Natl. Acad. Sci. U.S.A.* **107**, 2860–2865 (2010).

48. C. R. Midgett, K. M. Talbot, J. L. Day, G. P. Munson, F. J. Kull, Structure of the master regulator Rns reveals an inhibitor of enterotoxigenic *Escherichia coli* virulence regulons. *Sci. Rep.* **11**, 15663 (2021).

49. A. S. Reddy, S. Zhang, Polypharmacology: Drug discovery for the future. *Expert Rev. Clin. Pharmacol.* **6**, 41–47 (2013).

50. S. Carden, C. Okoro, G. Dougan, D. Monack, Non-typhoidal *Salmonella* Typhimurium ST313 isolates that cause bacteremia in humans stimulate less inflammasome activation than ST19 isolates associated with gastroenteritis. *Pathog. Dis.* **73**, ftu023 (2015).

51. C. V. Pulford, B. M. Perez-Sepulveda, R. Canals, J. A. Bevington, R. J. Bengtsson, N. Wenner, E. V. Rodwell, B. Kumwenda, X. Zhu, R. J. Bennett, G. E. Stenhouse, P. Malaka De Silva, H. J. Webster, J. A. Bengoechea, A. Dumigan, A. Tran-Dien, R. Prakash, H. C. Banda,

L. Alufandika, M. P. Mautanga, A. Bowers-Barnard, A. Y. Beliavskaia, A. V. Predeus, W. P. M. Rowe, A. C. Darby, N. Hall, F.-X. Weill, M. A. Gordon, N. A. Feasey, K. S. Baker, J. C. D. Hinton, Stepwise evolution of *Salmonella* Typhimurium ST313 causing bloodstream infection in Africa. *Nat. Microbiol.* **6**, 327–338 (2021).

52. S. Van Puyvelde, T. de Block, S. Sridhar, M. Bawn, R. A. Kingsley, B. Ingelbeen, M. A. Beale, B. Barbé, H. J. Jeon, L. Mbuyi-Kalonji, M.-F. Phoba, D. Falay, D. Martiny, O. Vandenberg, D. Affolabi, J. P. Rutanga, P.-J. Ceyssens, W. Mattheus, W. L. Cuypers, M. A. B. van der Sande, S. E. Park, S. Kariuki, K. Otieno, J. P. A. Lusingu, J. R. Mbwana, S. Adjei, A. Sarfo, S. O. Agyei, K. P. Asante, W. Otieno, L. Otieno, M. C. Tahita, P. Lompo, I. F. Hoffman, T. Mvalo, C. Msefula, F. Hassan-Hanga, S. Obaro, G. Mackenzie, S. Deborggraeve, N. Feasey, F. Marks, C. A. Mac Lennan, N. R. Thomson, J. Jacobs, G. Dougan, S. Kariuki, O. Lunguya, A genomic appraisal of invasive *Salmonella* Typhimurium and associated antibiotic resistance in sub-Saharan Africa. *Nat. Commun.* **14**, 6392 (2023).

53. S. K. Hoiseth, B. A. D. Stocker, Aromatic-dependent *Salmonella typhimurium* are non-virulent and effective as live vaccines. *Nature* **291**, 238–239 (1981).

54. J. Noster, M. Persicke, T.-C. Chao, L. Krone, B. Heppner, M. Hensel, N. Hansmeier, Impact of ROS-induced damage of TCA cycle enzymes on metabolism and virulence of *Salmonella enterica* serovar typhimurium. *Front. Microbiol.* **10**, 762 (2019).

55. L. J. Worrall, M. Vuckovic, N. C. J. Strynadka, Crystal structure of the C-terminal domain of the *Salmonella* type III secretion system export apparatus protein InvA. *Protein Sci.* **19**, 1091–1096 (2010).

56. J. C. Shelley, A. Cholleti, L. L. Frye, J. R. Greenwood, M. R. Timlin, M. Uchimaya, Epik: A software program for pK_a prediction and protonation state generation for drug-like molecules. *J. Comput. Aided Mol. Des.* **21**, 681–691 (2007).

57. C. Lu, C. Wu, D. Ghoreishi, W. Chen, L. Wang, W. Damm, G. A. Ross, M. K. Dahlgren, E. Russell, C. D. Von Bargen, R. Abel, R. A. Friesner, E. D. Harder, OPLS4: Improving force field accuracy on challenging regimes of chemical space. *J. Chem. Theory Comput.* **17**, 4291–4300 (2021).

58. R. A. Friesner, R. B. Murphy, M. P. Repasky, L. L. Frye, J. R. Greenwood, T. A. Halgren, P. C. Sanschagrin, D. T. Mainz, Extra precision glide: Docking and scoring incorporating a model of hydrophobic enclosure for protein−ligand complexes. *J. Med. Chem.* **49**, 6177–6196 (2006).

59. R. A. Friesner, J. L. Banks, R. B. Murphy, T. A. Halgren, J. J. Klicic, D. T. Mainz, M. P. Repasky, E. H. Knoll, M. Shelley, J. K. Perry, D. E. Shaw, P. Francis, P. S. Shenkin, Glide: A new approach for rapid, accurate docking and scoring. 1. Method and assessment of docking accuracy. *J. Med. Chem.* **47**, 1739–1749 (2004).

60. I.-M. A. Chen, K. Chu, K. Palaniappan, M. Pillay, A. Ratner, J. Huang, M. Huntemann, N. Varghese, J. R. White, R. Seshadri, T. Smirnova, E. Kirton, S. P. Jungbluth, T. Woyke, E. A. Eloe-Fadrosh, N. N. Ivanova, N. C. Kyrpides, IMG/M v.5.0: An integrated data management and comparative analysis system for microbial genomes and microbiomes. *Nucleic Acids Res.* **47**, D666–D677 (2019).

61. Y. Huang, B. Niu, Y. Gao, L. Fu, W. Li, CD-HIT Suite: A web server for clustering and comparing biological sequences. *Bioinformatics* **26**, 680–682 (2010).

62. R. C. Edgar, MUSCLE: A multiple sequence alignment method with reduced time and space complexity. *BMC Bioinformatics* **5**, 113 (2004).

63. S. Guindon, J.-F. Dufayard, V. Lefort, M. Anisimova, W. Hordijk, O. Gascuel, New algorithms and methods to estimate maximum-likelihood phylogenies: Assessing the performance of PhyML 3.0. *Syst. Biol.* **59**, 307–321 (2010).

64. D. Darriba, G. L. Taboada, R. Doallo, D. Posada, ProtTest 3: Fast selection of best-fit models of protein evolution. *Bioinformatics* **27**, 1164–1165 (2011).

65. S. Malmsheimer, I. Grin, E. Bohn, M. Franz-Wachtel, B. Macek, T. Sahr, F. Smollich, D. Chetrit, A. Meir, C. Roy, C. Buchrieser, S. Wagner, The T4bSS of *Legionella* features a two-step secretion pathway with an inner membrane intermediate for secretion of transmembrane effectors. *PLOS Pathog.* **20**, e1012118 (2024).

66. J. V. Monjarás Feria, M. D. Lefebre, Y.-D. Stierhof, J. E. Galán, S. Wagner, Role of autocleavage in the function of a type III secretion specificity switch protein in *Salmonella enterica* serovar typhimurium. *mBio* **6**, e01459–e01415 (2015).

67. S. Wagner, L. Königsmaier, M. Lara-Tejero, M. Lefebre, T. C. Marlovits, J. E. Galán, Organization and coordinated assembly of the type III secretion export apparatus. *Proc. Natl. Acad. Sci. U.S.A.* **107**, 17745–17750 (2010).

68. M. Varadi, S. Anyango, M. Deshpande, S. Nair, C. Natassia, G. Yordanova, D. Yuan, O. Stroe, G. Wood, A. Laydon, A. Žídek, T. Green, K. Tunyasuvunakool, S. Petersen, J. Jumper, E. Clancy, R. Green, A. Vora, M. Lutfi, M. Figurnov, A. Cowie, N. Hobbs, P. Kohli, G. Kleywegt, E. Birney, D. Hassabis, S. Velankar, AlphaFold protein structure database: Massively expanding the structural coverage of protein-sequence space with high-accuracy models. *Nucleic Acids Res.* **50**, D439–D444 (2022).

69. H. J. Kwon, M. H. Bennik, B. Demple, T. Ellenberger, Crystal structure of the *Escherichia coli* Rob transcription factor in complex with DNA. *Nat. Struct. Biol.* **7**, 424–430 (2000).

70. W. K. Gillette, R. G. Martin, J. L. Rosner, Probing the *Escherichia coli* transcriptional activator MarA using alanine-scanning mutagenesis: Residues important for DNA binding and activation. *J. Mol. Biol.* **299**, 1245–1255 (2000).

71. S. Rhee, R. G. Martin, J. L. Rosner, D. R. Davies, A novel DNA-binding motif in MarA: The first structure for an AraC family transcriptional activator. *Proc. Natl. Acad. Sci. U.S.A.* **95**, 10413–10418 (1998).

72. M. Corbella, Q. Liao, C. Moreira, A. Parracino, P. M. Kasson, S. C. L. Kamerlin, The N-terminal helix-turn-helix motif of transcription factors MarA and Rob drives DNA recognition. *J. Phys. Chem. B* **125**, 6791–6806 (2021).

73. T. A. Halgren, Identifying and characterizing binding sites and assessing druggability. *J. Chem. Inf. Model.* **49**, 377–389 (2009).

74. W. L. Jorgensen, J. Chandrasekhar, J. D. Madura, R. W. Impey, M. L. Klein, Comparison of simple potential functions for simulating liquid water. *J. Chem. Phys.* **79**, 926–935 (1983).

75. T. Darden, D. York, L. Pedersen, Particle mesh Ewald: An $N \cdot \log(N)$ method for Ewald sums in large systems. *J. Chem. Phys.* **98**, 10089–10092 (1993).

76. H. J. C. Berendsen, J. P. M. Postma, W. F. van Gunsteren, A. DiNola, J. R. Haak, Molecular dynamics with coupling to an external bath. *J. Chem. Phys.* **81**, 3684–3690 (1984).

77. G. J. Martyna, M. L. Klein, M. Tuckerman, Nosé–Hoover chains: The canonical ensemble via continuous dynamics. *J. Chem. Phys.* **97**, 2635–2643 (1992).

78. G. J. Martyna, M. E. Tuckerman, D. J. Tobias, M. L. Klein, Explicit reversible integrators for extended systems dynamics. *Mol. Phys.* **87**, 1117–1157 (1996).

79. E. S. Lohan, J. Torres-Sospedra, P. Richter, H. Leppäkoski, J. Huerta, A. Cramariuc, Crowdsourced WiFi database and benchmark software for indoor positioning, Zenodo (2017); https://doi.org/10.5281/ZENODO.889798.

80. M. P. Jacobson, D. L. Pincus, C. S. Rapp, T. J. F. Day, B. Honig, D. E. Shaw, R. A. Friesner, A hierarchical approach to all-atom protein loop prediction. *Proteins* **55**, 351–367 (2004).

81. B. Miroux, J. E. Walker, Over-production of proteins in *Escherichia coli*: Mutant hosts that allow synthesis of some membrane proteins and globular proteins at high levels. *J. Mol. Biol.* **260**, 289–298 (1996).

82. I. N. Olekhnovich, R. J. Kadner, Crucial roles of both flanking sequences in silencing of the *hilA* promoter in *Salmonella enterica*. *J. Mol. Biol.* **357**, 373–386 (2006).

83. M. Mayer, B. Meyer, Characterization of ligand binding by saturation transfer difference NMR spectroscopy. *Angew. Chem. Int. Ed. Engl.* **38**, 1784–1788 (1999).

84. M. ElGamacy, M. Riss, H. Zhu, V. Truffault, M. Coles, Mapping local conformational landscapes of proteins in solution. *Structure* **27**, 853–865.e5 (2019).

85. B. Han, Y. Liu, S. W. Ginzinger, D. S. Wishart, SHIFTX2: Significantly improved protein chemical shift prediction. *J. Biomol. NMR* **50**, 43–57 (2011).

86. T. E. Wales, K. E. Fadgen, G. C. Gerhardt, J. R. Engen, High-speed and high-resolution UPLC separation at zero degrees celsius. *Anal. Chem.* **80**, 6815–6820 (2008).

87. S. J. Geromanos, J. P. C. Vissers, J. C. Silva, C. A. Dorschel, G. Li, M. V. Gorenstein, R. H. Bateman, J. I. Langridge, The detection, correlation, and comparison of peptide precursor and product ions from data independent LC-MS with data dependant LC-MS/MS. *Proteomics* **9**, 1683–1695 (2009).

88. G. Li, J. P. C. Vissers, J. C. Silva, D. Golick, M. V. Gorenstein, S. J. Geromanos, Database searching and accounting of multiplexed precursor and product ion spectra from the data independent analysis of simple and complex peptide mixtures. *Proteomics* **9**, 1696–1719 (2009).

89. D. F. Veber, S. R. Johnson, H.-Y. Cheng, B. R. Smith, K. W. Ward, K. D. Kopple, Molecular properties that influence the oral bioavailability of drug candidates. *J. Med. Chem.* **45**, 2615–2623 (2002).

90. M. Feldgarden, V. Brover, N. Gonzalez-Escalona, J. G. Frye, J. Haendiges, D. H. Haft, M. Hoffmann, J. B. Pettengill, A. B. Prasad, G. E. Tillman, G. H. Tyson, W. Klimke, AMRFinderPlus and the reference gene catalog facilitate examination of the genomic links among antimicrobial resistance, stress response, and virulence. *Sci. Rep.* **11**, 12728 (2021).

91. R. Platt, C. Drescher, S.-K. Park, G. J. Phillips, Genetic system for reversible integration of DNA constructs and *lacZ* gene fusions into the *Escherichia coli* chromosome. *Plasmid* **43**, 12–23 (2000).

92. G. Demarre, A.-M. Guérout, C. Matsumoto-Mashimo, D. A. Rowe-Magnus, P. Marlière, D. Mazel, A new family of mobilizable suicide plasmids based on broad host range R388 plasmid (IncW) and RP4 plasmid (IncPα) conjugative machineries and their cognate Escherichia coli host strains. *Res. Microbiol.* **156**, 245–255 (2005).

93. K. Kaniga, J. C. Bossio, J. E. Galán, The *Salmonella typhimurium* invasion genes *invF* and *invG* encode homologues of the AraC and PulD family of proteins. *Mol. Microbiol.* **13**, 555–568 (1994).

94. L. Krampen, S. Malmsheimer, I. Grin, T. Trunk, A. Lührmann, J.-W. de Gier, S. Wagner, Revealing the mechanisms of membrane protein export by virulence-associated bacterial secretion systems. *Nat. Commun.* **9**, 3467 (2018).
